# Genotypic variation and root traits associated with hypoxia tolerance in quinoa (*Chenopodium quinoa* Willd.)

**DOI:** 10.1371/journal.pone.0361654

**Published:** 2026-11-03

**Authors:** Van Loc Nguyen, Thi Hong Nhung Phan, Viet Long Nguyen, Thai Hoang Dinh, Thi Ngoc Dinh Nguyen, Thi Thu Ha Phung, Trung Duc Nguyen, Duc Ha Chu, Ramiro Curti, Daniel Bertero

**Affiliations:** 1 Faculty of Agronomy, Vietnam National University of Agriculture, Hanoi, Vietnam; 2 Institute of Biology and Agricultural Technology, Vietnam National University of Agriculture, Hanoi, Vietnam; 3 Faculty of Agricultural Technology, University of Engineering and Technology, Vietnam National University Hanoi, Hanoi, Vietnam; 4 Escuela de Agronomía, National University of Salta, Salta, Argentina; 5 Depto. de Producción Vegetal, Facultad de Agronomía, Universidad de Buenos Aires and IFEVA-Conicet, Buenos Aires, Argentina; Brigham Young University, UNITED STATES OF AMERICA

## Abstract

Waterlogging-induced hypoxia is becoming an increasingly important constraint to crop production under climate change. Although quinoa (*Chenopodium quinoa* Willd.) is recognized for its tolerance to drought and salinity, its response to hypoxia remains poorly understood. This study evaluated the effects of hypoxia on growth, root development, biomass accumulation, and individual seed yield in 28 quinoa genotypes. Hypoxia reduced plant height by 31%, leaf area by 47%, root length by 39%, and shoot dry weight by 37% (mean hypoxia tolerance index 0.69, 0.53, 0.61, and 0.63); shoot dry weight remained 44% lower after recovery, whereas root diameter increased by 28% under hypoxia. Correlation analysis revealed that root length and root surface area were positively associated with individual seed yield, whereas root diameter was negatively related to several growth traits, highlighting the importance of maintaining root development under hypoxia. Permutational multivariate analysis of variance across 19 traits attributed 37.1% of overall phenotypic variation to genotype, 30.0% to oxygen regime, and 18.8% to their interaction (each p = 0.001), and hierarchical clustering on hypoxia tolerance indices resolved three genotype groups with contrasting trait profiles. Under hypoxia, Amachuha and Isluga produced the highest individual yields, followed by EDK4 and Chadmo. Under genotype-level validation (leave-one-genotype-out, leave-one-cluster-out, and highland-versus-lowland cross-ecotype splits), no model predicted the individual yield of genotypes excluded from training better than the mean yield of the training genotypes under the same oxygen regime, so trait-based yield prediction does not transfer to unseen quinoa genotypes. These findings provide valuable insights into the mechanisms of hypoxia tolerance and identify promising genetic resources for breeding quinoa cultivars adapted to waterlogging-prone environments.

## Introduction

Climate change has intensified the exposure of crop production systems to drought, salinity, and waterlogging [1]. These constraints are particularly important in lowland and coastal agricultural regions where unstable rainfall, sea-level rise, and poor drainage increasingly threaten crop establishment and yield stability [2]. Under waterlogged conditions, oxygen diffusion in soil declines sharply, and root-zone hypoxia develops within a short period [3,4]. Oxygen deficiency (hypoxia) restricts aerobic respiration, reduces adenosine triphosphate (ATP) production, disrupts root growth, and limits water and nutrient uptake [5]. It also promotes anaerobic metabolism and the accumulation of toxic fermentation products that impair plant development. Consequently, hypoxia can reduce leaf expansion, root elongation, biomass accumulation, and yield [6,7]. Previous studies have demonstrated substantial genetic variation in hypoxia tolerance across crop species such as rice [8], maize [9], soybean [10], wheat [11], and barley [12], with tolerant genotypes generally maintaining better root growth, photosynthetic capacity, biomass accumulation, and post-stress recovery under oxygen-deficient conditions. In quinoa, research has primarily focused on drought and salinity tolerance, whereas studies on hypoxia remain limited despite evidence of significant genotypic variation in growth and physiological responses to oxygen deficiency [13]. However, comprehensive evaluations that simultaneously assess plant performance during hypoxic stress and recovery across diverse quinoa germplasm remain scarce.

Quinoa (*Chenopodium quinoa* Willd.) has attracted considerable attention as a climate-resilient crop due to its superior nutritional value, wide genetic diversity, and adaptability to harsh environments [14]. Compared with many traditional cereals, quinoa exhibits superior tolerance to drought, salinity, and nutrient-poor soils, making it a promising candidate for diversification of agricultural systems under current climate [15,16]. In Vietnam, quinoa has been proposed for cultivation in the northern coastal regions, the Central Highlands, and the Mekong Delta, where agricultural production is increasingly challenged by environmental stresses [13,17-18]. Despite its reputation as a stress-tolerant crop, quinoa remains vulnerable to waterlogging, a condition that rapidly induces hypoxia in the root zone and restricts plant growth and productivity [19]. Previous studies have shown that oxygen deficiency reduces biomass accumulation, root development, and yield in quinoa [13,20]; however, these investigations were generally limited to a small number of genotypes [20]. As a result, the extent of genetic variation for hypoxia tolerance remains largely unknown. Furthermore, although root traits are among the first plant characteristics affected by oxygen deficiency, their contribution to yield maintenance and adaptation under hypoxia has not been systematically evaluated. Therefore, identifying root traits associated with hypoxia tolerance and determining genotypes that maintain performance under hypoxia are important prerequisites for the development of quinoa cultivars adapted to waterlogging-prone environments.

In addition, the large genetic diversity present in quinoa suggests that substantial variation may exist in the response to contrasting oxygen regimes, yet comprehensive evaluations of genotype × oxygen-regime interactions are still scarce. Recent advances in multivariate analyses and machine-learning techniques provide powerful opportunities to identify superior genotypes, characterize adaptation patterns, and uncover complex trait-yield relationships that are difficult to detect using conventional statistical approaches [21–23]. Therefore, this study aimed to evaluate the responses of a diverse quinoa genotypes to hypoxia, identify key root and growth traits associated with tolerance, characterize genotype × oxygen-regime effects across correlated traits using multivariate analysis of variance and hierarchical clustering, and compare machine-learning algorithms for predicting individual seed yield performance under hypoxia. By integrating morphological, physiological, and biomass-related trait assessments during both hypoxic stress and post-stress recovery with multivariate analyses and machine learning-based prediction, this study provides a comprehensive framework for identifying hypoxia-tolerant quinoa genotypes and supporting breeding strategies for improved waterlogging tolerance.

## Materials and methods

### Plant materials

Twenty-eight genotypes with name and passport data were used in this study (Table 1). Of these, Q1, Q4, Q4bias, and Q5 were selected by Dr. Redouane Choukrallah at the International Center for Biosaline Agriculture (ICBA, UAE) based on their superior performance under high-temperature conditions [24]. G13, G18, and Black quinoa were derived from interspecific crosses between *Chenopodium quinoa* and *Chenopodium berlandieri*, with G13 and G18 provided by Dr. Eric Jellen (Brigham Young University, USA) [19]. Cahuil and Isluga are Chilean germplasm originating from central and northern Chile, respectively, whereas NL-6 was selected from the humid lowlands of Chile and Sayaña originated from the Bolivian Highlands. Atlas is a breeding line developed for northwestern European environments, while 2-Want is regarded as a natural hybrid between Andean and Lowland quinoa [17,25–27]. In addition, “Real1 x Ruy937” and “Surami x Ruy937” are stable F8 recombinant inbred lines (RILs) derived from their respective parental crosses and have been used in previous study investigating quinoa responses to abiotic stresses [13]. All quinoa genotypes were provided through a project funded by the Vietnamese Ministry of Science and Technology (Grant No. HNQT/SPĐP/07.17) under the bilateral cooperation program between the Governments of Vietnam and Argentina for the evaluation of quinoa under different ecological conditions in Vietnam.

**Table 1:** Name and passport data of quinoa genotype.

No.	Genotype	Origin	Ecotype
1	23-GR	Chile x Bolivia	Lowland
2	2-Want	Bolivia	Lowland
3	37-Test	Chile x Bolivia	highland
4	42-Test	Chile x Bolivia	Lowland
5	59-ALC	Chile x Bolivia	highland
6	Amachuha	Bolivia	highland
7	Atlas	Netherland	Lowland
8	Black quinoa	Bolivia	Lowland
9	Cahuil	Chile	highland
10	Chadmo	Chile	highland
11	EDK-4	Netherland	Lowland
12	G13	Argentina	Lowland
13	G18	Argentina	Lowland
14	Isluga	Chile	highland
15	Leucaboldo	Chile	highland
16	NL-6	Netherland	Lowland
17	Puno	Denmark	highland
18	Q1	Argentina	Lowland
19	Q4	Argentina	Lowland
20	Q4bias	Argentina	Lowland
21	Q5	Argentina	Lowland
22	Real1*Ruy 937	US	Lowland
23	Red	Argentina	highland
24	Riobamba	Netherland	highland
25	Ru-2	United Kingdom	highland
26	Sayaña	Bolivia	highland
27	Surami*Ruy937	US	Lowland
28	Unknown	Argentina	highland

### Experimental method

To ensure uniform germination, 100 seeds of each genotype were soaked in distilled water for 3 h and then germinated on moist tissue paper for 2 days at room temperature (~28°C). Uniformly germinated seeds with radicles ~1.5 cm in length were selected for the experiment.The germinations were inserted through holes punched in a 7-mm-thick polystyrene board and held upright with plugs of silicone rubber. Each board, holding 96 seedlings, were placed over an opaque plastic container (386 mm × 256 mm × 135 mm), with the roots in deionized distilled water continuously aerated at 1.0 L min^−1^ by air pumps. Then, 14-day-old seedlings were grown for an additional 14 days under either hypoxia or normoxia under hydroponic culture (Fig 1). The nutrient solution in hydroponic culture was prepared by adding 2 mL L^−1^ each of A and B stock solutions to distilled water. A stock solution contained 37.51 g L^−^^1^ NO_3_− -N, 43.54 g L^−1^ Ca, 39.99 g L^−1^ K_2_O, and 0.98 g L^−1^ Fe-EDTA, whereas stock solution B contained 10.50 g L^−1^ P_2_O_5_, 2.00 g L^−1^ NH_4_⁺ -N, 6.68 g L^−1^ S, 5.00 g L^−1^ Mg, 394 mg L^−1^ Mn, 140 mg L^−1^ B, 100 mg L^−1^ Zn, 24 mg L^−1^ Cu, and 20 mg L^−1^ Mo. Under normoxia, seedlings were aerated by two air pumps at opposite ends of the container to maintain dissolved oxygen (DO) concentration of >7.0 mg L^−1^. Under hypoxia, seedlings were grown in 0.1% (w/v) agar solution in deionized distilled water that is deoxygenated with a continuous flow of nitrogen to maintain a DO concentration of <1.0 mg L^−1^. The experiment was arranged as a randomized complete block design (RCBD) with three replicates. Each replicate (block) comprised a pair of hydroponic containers placed side by side, one maintained under normoxia and the other under hypoxia. Genotypes were randomly allocated to experimental units within each container, and each experimental unit consisted of six plants. Samples were collected at the end of the hypoxia treatment and again 14 days after recovery. The remaining plants were continuously maintained under normoxia in plastic containers supplied with nutrient solution until physiological maturity, when the plants had completely senesced, and grain yield was determined at harvest. At each sampling time, five plants per replication were harvested for trait measurements.

**Fig 1:**
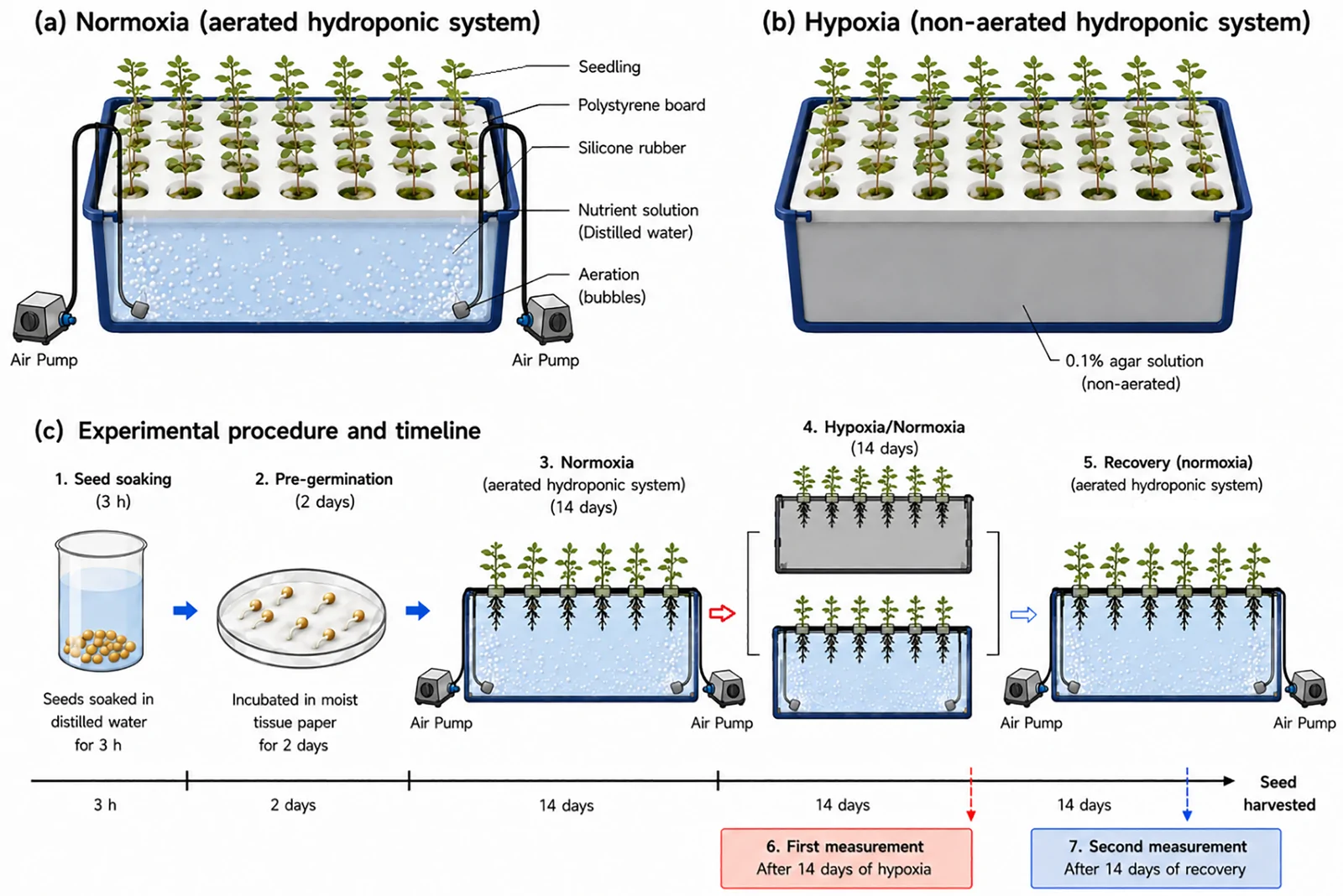
Experimental design: (a) Aerated hydroponic system (normoxia); (b) Non-aerated hydroponic system containing 0.1% agar solution (hypoxia); and (c) Experimental procedure and sampling timeline, including seed soaking (3 h), pre-germination (2 days), normoxia acclimation (14 days), hypoxia or normoxia treatment (14 days), recovery under normoxia (14 days), and measurements after treatment and recovery.

### Measurements

Root traits were measured at the end of hypoxia treatments and the 14^th^ day of recovery. The root systems of individual plants were scanned with an image scanner (GTX-970 scanner, Epson, Nagano, Japan) under the control of WinRhizo software (Regent Instruments, Inc., Quebec, Canada). Root length (RL, cm) was measured on the so called skeleton images of root systems. Roots were classified into ten groups with unequal width (±, mm): 0 > ± ≦ 0.5, 0.5 > ± ≦ 1.0, 1.0 > ± ≦ 1.5, 1.5> and ≦2.0, 2.0 > ± ≦ 2.5, 2.5 > ±≦3.0, 3.0 > ± ≦ 3.5, 3.5 > ± ≦ 4.0, 4.0 > ± ≦ 4.5, ± > 4.5) for evaluation of root diameter (RD, mm). Root surface area (RSA, cm^2^) was automatically calculated by software based on RL and RD of root in each class. Growth traits, including plant heigh (PH, cm), number of leave of main stem (NoL, leaves main-stem^-1^), number of branches (NoB, branches plant^-1^) were determined at the end of hypoxia treatments and the 14^th^ day of recovery. PH is the height of the main plant from the surface of the soil pot to the top of the main plant stem. Stem diameter (SD) was measured at a 2 cm height. We used 1 cm length and axillary bud as thresholds for counting NoL and NoB, respectively. Physiologcial traits including leaf area (LA, cm^2^) and SPAD values were measured. LA as determined using a leaf area meter (CID202, USA). SPAD value was recorded by using SPAD 502 (Konica Minolta, Japan). Roots and shoots were dried in oven at 80 °C until constant weight. Then root dry weight (RDW, g plant^-1^), shoot dry weight (SDW, g plant^-1^) and total dry weight (TDW, g plant^-1^) were calculated. The individual seed yield (IY, g plant^-1^) was determined at the end of the experiment.

### Data analysis

#### Hyoxia tolerance index-HTI analysis.

HTI was calculated as the ratio of the trait value under hypoxia to that under normoxia (HTI = X_H_/X_N_), where X_H_ and X_N_ represent the average values of the measured trait under hypoxia and normoxia, respectively.

#### Statistical analyses.

The environment (E) refers to the two oxygen regimes to which seedlings were exposed, normoxia and hypoxia, rather than multiple field locations or growing seasons as in a conventional multi-environment trial. Statistical analyses were performed using R version 4.5.1 to assess the effects of genotype, oxygen regime, and their interaction using the following model:


$$\begin{array}{c} Y_{ijk}=\mu +{Rep}_{k}+G_{i}+E_{j}+{\left(GE\right)}_{ij}+\varepsilon_{ijk} \end{array}$$ (1)

where *Yijk* is the observed value of the trait for genotype i in oxygen regime j and replicate k; *µ* is the grand mean; *Rep*_*k*_ is the effect of replicate *k*; *G*_*i*_ and *E*_*j*_ are the main effects of genotype i and oxygen regime j, respectively; (GE)_*ij*_ is the genotype × oxygen-regime interaction effect; and *ε*_*ijk*_ is the random residual error. An analysis of variance (ANOVA) based on this model was conducted to determine the significance of genotype, oxygen regime, and genotype × oxygen-regime effects on the measured traits. When significant effects were detected, mean comparisons were made using the Least Significant Difference (LSD) test at a 5% significance level with the “agricolae” package (v1.3.7). Agreement between genotype rankings under the two oxygen regimes was assessed with Spearman’s rank correlation, and the association between cluster membership and ecotype with Fisher’s exact test.

#### Hierarchical clustering and principal component analysis.

Principal component analysis (PCA) was performed on the genotype × oxygen-regime mean values of 20 traits (56 observations), standardized to zero mean and unit variance, with the FactoMineR package (v2.16) and drawn as a biplot of trait loadings and observation scores with ggplot2 (v4.0.3). Hierarchical clustering on principal components (HCPC; FactoMineR v2.16, factoextra v2.2.0) was applied to the per-genotype hypoxia tolerance indices of the 22 measured traits: the hierarchical tree (Ward’s criterion) was cut into three clusters and the partition was consolidated by k-means.

#### Multivariate analysis of variance.

To account for correlations among the measured traits, a permutational multivariate analysis of variance (PERMANOVA; Anderson 2001) was fitted to the 19 traits in Table 2 with the vegan package (v2.7.5; adonis2; Euclidean distance on standardized traits; terms added sequentially as replicate + genotype × oxygen regime; 999 permutations). A classical MANOVA (Pillai’s trace) on the same model was computed for comparison.

**Table 2:** Mean squares for genotype, oxygen regime, and their interaction for all variables evaluated in the present study.

Trait	Mean squares		
	*Genotype (G)*	*Oxygen regime (E)*	*G × E*
Root length after 2 weeks of hypoxia treatment (RL1, cm)	216863.74***	4859399.91***	114230.77***
Root length after 2 weeks of recovery (RL2, cm)	314903.15***	7233875.80***	120195.86***
Root surface area after 2 weeks of hypoxia treatment (RSA1, cm²)	671.97***	6108.15***	320.82***
Root surface area after 2 weeks of recovery (RSA2, cm²)	493.08***	14522.96***	299.68***
Root diameter after 2 weeks of hypoxia treatment (RD1, mm)	8.65 × 10 ^−3^***	0.08***	2.24 × 10 ^−3^***
Root diameter after 2 weeks of recovery (RD2, mm)	1.50 × 10 ^−3^***	4.82 × 10 ^−5^ ns	7.71 × 10 ^− 4^***
Plant height after 2 weeks of hypoxia treatment (PH1, cm)	93.09***	2664.05***	21.08***
Plant height after 2 weeks of recovery (PH2, cm)	297.01***	7189.29***	100.50***
Number of leaves after 2 weeks of hypoxia treatment (NoL1, leaves plant ^−1^)	15.01***	141.17***	11.14***
Number of leaves after 2 weeks of recovery (NoL2, leaves plant ^−1^)	34.33***	1005.48***	21.10***
Leaf area after 2 weeks of hypoxia treatment (LA1, cm²)	2031.39***	105251.65***	685.00***
Leaf area after 2 weeks of recovery (LA2, cm²)	4824.01***	195714.39***	2133.18***
SPAD after 2 weeks of hypoxia treatment (SPAD1)	38.12***	688.10***	20.30**
SPAD after 2 weeks of recovery (SPAD2)	41.29***	293.62***	29.64***
Root dry weight after 2 weeks of hypoxia treatment (RDW1, g plant ^−1^)	6.34 × 10^−4^***	0.02***	3.59 × 10^−4^***
Root dry weight after 2 weeks of recovery (RDW2, g plant ^−1^)	3.67 × 10 ^−3^***	0.07***	3.08 × 10 ^−3^***
Shoot dry weight after 2 weeks of hypoxia treatment (SDW1, g plant ^−1^)	0.05***	1.33***	0.03***
Shoot dry weight after 2 weeks of recovery (SDW2, g plant ^−1^)	0.36***	17.32***	0.25***
Individual seed yield (IY, g plant ^−1^)	3.30***	47.09***	1.66***

The asterisks indicate significance at ***p < 0.001, **p < 0.01, *p < 0.05. ns: Non-significant.

#### Predictive model analysis.

To identify the most suitable predictive model for estimating IY, nine regression algorithms representing linear, regularized linear, kernel-based, tree-based, instance-based, and neural-network approaches were evaluated. These included Ordinary Least Squares (OLS), Ridge Regression, Least Absolute Shrinkage and Selection Operator (LASSO), Elastic Net, k-Nearest Neighbors (KNN), Support Vector Machine with a linear kernel (SVM-Linear), Random Forest (RF), Gradient Boosting Machine (GBM), and Multi-Layer Perceptron (MLP).

Prior to model training, all predictor variables were screened for missing values and standardized to zero mean and unit variance to ensure comparability among features and to avoid scale-related bias. The OLS model was used as the baseline linear regression approach. Ridge, LASSO, and Elastic Net were implemented to evaluate the effects of L2 regularization, L1 regularization, and combined regularization, respectively. RF and GBM were employed to capture complex nonlinear interactions among predictor variables through ensemble tree-learning strategies. KNN was used as a distance-based non-parametric method, while SVM-Linear was adopted to evaluate linear margin-based regression performance. The MLP model was implemented to assess the ability of artificial neural networks to learn nonlinear relationships between predictors and IY.

The models were used to test whether the measured seedling traits could predict the individual yield of genotypes not used in training, within the same oxygen regime. Because each genotype contributed six observations (two oxygen regimes × three replicates), models were evaluated at the genotype level in three ways: (i) leave-one-genotype-out, in which, for each of the 28 genotypes in turn, all of its observations were withheld, the model was trained on the remaining 27 genotypes, and yield was predicted for the withheld genotype; (ii) leave-one-cluster-out, in which each of the three HCPC genotype clusters (see Results) was withheld in turn; and (iii) cross-ecotype, in which models were trained on the 13 highland genotypes and tested on the 15 lowland genotypes, and vice versa. Hyperparameters were selected within each training set by grid search with five-fold cross-validation in which folds were grouped by genotype, so that model selection and performance estimation never shared data (Varma and Simon 2006). Each design was compared with a reference predictor that used no trait information (the mean yield of the training observations under the same oxygen regime) and with a random 80/20 split of individual observations.

Model performance was assessed on the withheld observations of each validation design using four complementary metrics: Pearson’s correlation coefficient (r), coefficient of determination (R^2^), root mean square relative error (RMSRE), and statistical significance (p-value). Pearson’s correlation coefficient quantified the strength of the linear association between observed and predicted values. The coefficient of determination (R^2^) measured the proportion of variance explained by each model. RMSRE was used to evaluate prediction accuracy while accounting for relative error magnitude. Scatter plots of observed versus predicted values were generated for all models. A 1:1 reference line was included to visually assess prediction agreement and systematic bias. Models exhibiting higher r and R^2^ values together with lower RMSRE values were considered to have superior predictive performance. All analyses were performed in Python (version 3.12.10) using the scikit-learn (v1.9.0), pandas (v2.2.3), NumPy (v2.4.6), and SciPy (v1.18.0) libraries.

## Results

### Analysis for variance

The results of ANOVA showed that genotype and oxygen-regime effects were highly significant (p < 0.001) for all measured traits except RD after 2 weeks of recovery (RD2), for which the oxygen-regime effect was not significant (Table 2). Significant genotype × oxygen-regime interactions were detected for all traits. Replicate effects were not significant for any trait except RDW1 (p < 0.001).

### Effects of hypoxia stresses on measured traits of quinoa genotypes

Hypoxia reduced root growth, but the response varied among root traits (Table 3; S1 Table). RL declined during hypoxia (mean HTI = 0.61, a 39% reduction) and remained 30% lower after recovery (HTI = 0.70). RSA was reduced less during hypoxia (HTI = 0.75) and to a similar extent after recovery (HTI = 0.69), whereas RD increased under hypoxia (HTI = 1.28) and returned to the normoxia level after recovery (HTI = 1.01).

**Table 3:** Panel means of 28 quinoa genotypes under normoxia and hypoxia and mean hypoxia tolerance index (HTI) for 18 traits.

Trait	Normoxia	Hypoxia	Mean HTI	Change (%)
RL1 (cm)	790.4	450.2	0.607	−39.3
RSA1 (cm²)	41.14	29.08	0.753	−24.7
RD1 (mm)	0.172	0.216	1.281	+28.1
RL2 (cm)	1287.0	872.0	0.700	−30.0
RSA2 (cm²)	55.96	37.36	0.695	−30.5
RD2 (mm)	0.141	0.140	1.006	+0.6
PH1 (cm)	25.49	17.52	0.694	−30.6
PH2 (cm)	47.04	33.95	0.728	−27.2
NoL1	16.48	14.64	0.901	−9.9
NoL2	25.07	20.18	0.817	−18.3
LA1 (cm²)	106.3	56.28	0.534	−46.6
LA2 (cm²)	183.7	115.4	0.642	−35.8
SPAD1	48.17	44.12	0.918	−8.2
SPAD2	49.23	46.59	0.949	−5.1
SDW1 (g plant ^−1^)	0.452	0.274	0.632	−36.8
RDW1 (g plant ^−1^)	0.055	0.033	0.642	−35.8
SDW2 (g plant ^−1^)	1.375	0.733	0.564	−43.6
RDW2 (g plant ^−1^)	0.119	0.078	0.741	−25.9

*Suffix 1, end of the 14-day hypoxia treatment (30 days after sowing, DAS); suffix 2, after 14 days of recovery (44 DAS). Normoxia and hypoxia columns are means of the 28 genotype means (three replicates each). HTI is the mean over genotypes of each genotype’s hypoxia/normoxia ratio (**S1**–**S4 Tables**) and differs from the ratio of the two panel means (e.g., RL1: 450.2/790.4 = 0.570 versus mean HTI 0.607). Change (%) = (HTI − 1) × 100. Individual seed yield is shown in* Fig 2.

Hypoxia also suppressed shoot growth (Table 3; S2 Table). PH decreased during hypoxia (HTI = 0.69, a 31% reduction) and remained 27% lower after recovery (HTI = 0.73), while NoL decreased by 10% and 18% under hypoxia and after recovery, respectively (HTI = 0.90 and 0.82). Despite the overall reduction, the extent of growth inhibition varied among genotypes (S2 Table), reflecting substantial genetic variation in shoot tolerance.

LA was more sensitive to hypoxia than SPAD value (Table 3; S3 Table). Mean LA declined by 47% during hypoxia (HTI = 0.53) and remained 36% lower after recovery (HTI = 0.64). In contrast, SPAD values showed only minor reductions, with mean HTI values of 0.92 and 0.95 for SPAD1 and SPAD2, respectively, indicating that chlorophyll content was largely maintained despite restricted leaf development.

Biomass accumulation was also strongly affected by hypoxia (Table 3; S4 Table). SDW decreased by 37% during hypoxia (HTI = 0.63) and remained 44% lower after recovery (HTI = 0.56). RDW showed reductions of 36% and 26% (HTI = 0.64 and 0.74 before and after recovery, respectively). Nevertheless, biomass responses differed considerably among genotypes (S4 Table), suggesting variation in the ability to maintain carbon accumulation under hypoxia.

IY differed significantly among quinoa genotypes under both normoxia and hypoxia (Fig 2). Hypoxia lowered IY in 26 of the 28 genotypes (Isluga and Riobamba were higher under hypoxia) and reordered them: genotype rankings under normoxia and hypoxia were weakly correlated (Spearman’s ρ = 0.25, p = 0.21), with a median shift of six rank positions. Amachuha was the highest-yielding genotype under hypoxia (5.26 g plant ^−1^) and second under normoxia (6.20 g), whereas Chadmo, the highest under normoxia (6.62 g), ranked fourth under hypoxia (4.55 g), behind Isluga (4.89 g) and EDK4 (4.57 g). Q4, Atlas, Sayaña, Cahuil, and Red ranked lowest under hypoxia. These results demonstrate substantial genotypic variation in yield performance under hypoxia.

**Fig 2:**
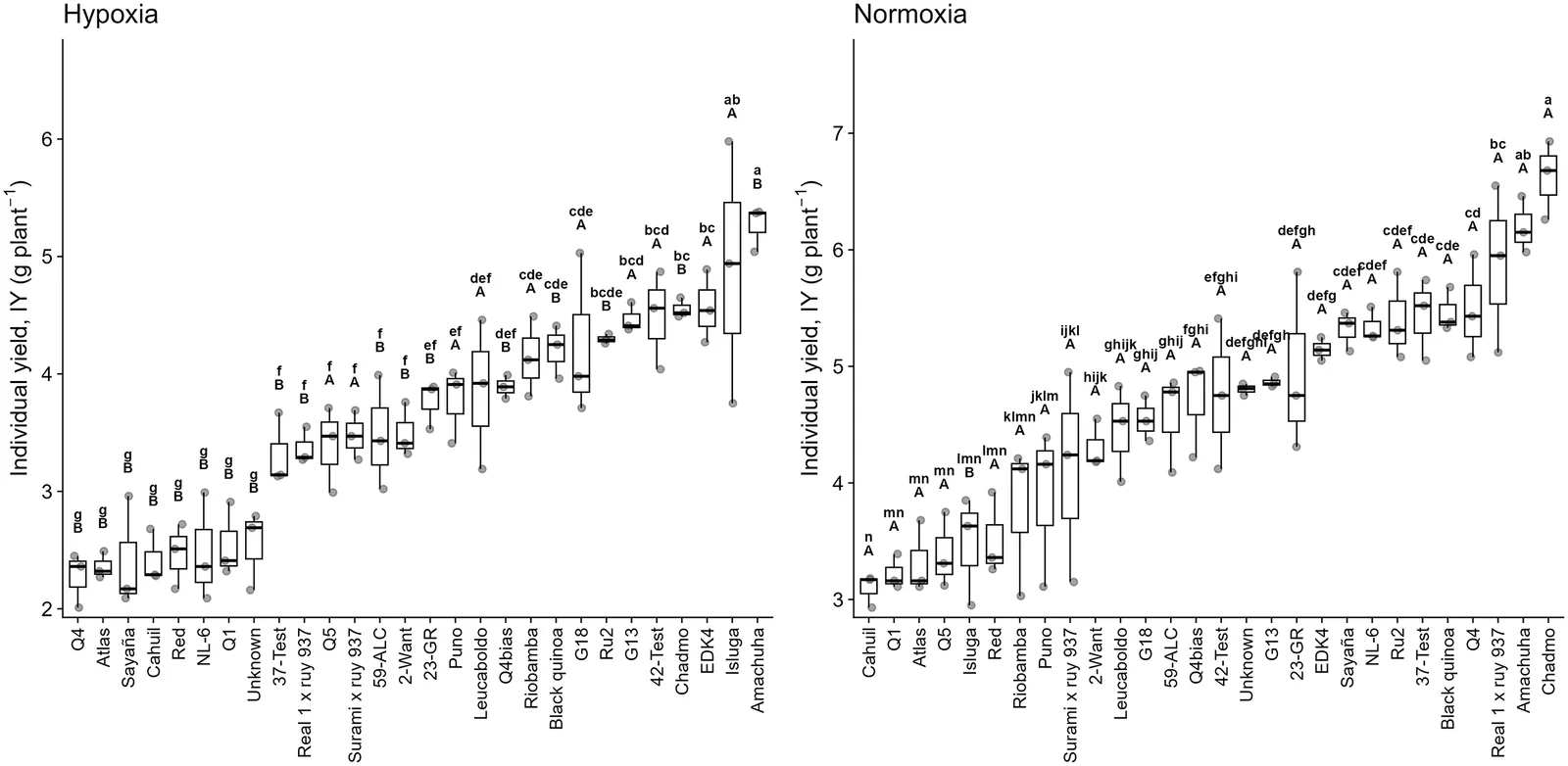
Individual seed yield (IY, g plant ^−1^) of 28 quinoa genotypes under hypoxia (left panel) and normoxia (right panel). Genotypes are ordered from lowest to highest mean IY within each panel, so the order differs between panels; the y-axis ranges also differ. Each gray point is one replicate value (three replicate blocks per genotype and oxygen regime, n = 3). Boxes span the interquartile range of the three values, the thick horizontal line is the median, and whiskers extend to the minimum and maximum. Letters above each box are from least significant difference (LSD) tests at α = 0.05. Lowercase letters compare genotypes within the same oxygen regime (one-way ANOVA of IY on genotype within each regime); genotypes sharing a letter do not differ significantly. Uppercase letters compare hypoxia with normoxia within each genotype, using the pooled residual mean square of the ANOVA model of Eq. 1 (replicate + genotype × oxygen regime): A marks the higher regime mean and B the significantly lower one, and both regimes are labeled A when they do not differ. The uppercase letter of a genotype therefore appears in both panels.

### Multivariate analysis of variance

Across the 19 traits jointly, PERMANOVA attributed 37.1% of total phenotypic variation to genotype, 30.0% to oxygen regime, and 18.8% to the genotype × oxygen-regime interaction (each p = 0.001); the replicate term accounted for 0.4% (p = 0.11) and the residual for 13.7% (Table 4). The classical MANOVA confirmed all three effects (Pillai’s trace: oxygen regime 0.991, genotype 11.22, interaction 9.51; all p < 0.001; Table 5) and also detected a replicate effect (p = 0.007).

**Table 4:** Permutational multivariate analysis of variance (PERMANOVA) of 19 traits of 28 quinoa genotypes under two oxygen regimes.

Source	Df	Sum of squares	R²	F	P
Replicate	2	12.2	0.004	1.54	0.106
Genotype	27	1176.6	0.371	11.00	0.001
Oxygen regime	1	953.1	0.300	240.62	0.001
Genotype × oxygen regime	27	595.4	0.188	5.57	0.001
Residual	110	435.7	0.137		
Total	167	3173.0	1.000		

*The 19 traits of* Table 2*, standardized to zero mean and unit variance; Euclidean distance; terms added sequentially (replicate + genotype × oxygen regime); 999 permutations (vegan, adonis2). R^2^ is the share of the total multivariate sum of squares.*

**Table 5:** Multivariate analysis of variance (MANOVA, Pillai’s trace) of the 19 traits of Table 2 for the same model as Table 4.

Source	df	Pillai’s trace	Approximate F	num df	den df	P
Replicate	2	0.531	1.77	38	186	0.007
Genotype	27	11.216	5.87	513	2090	< 0.001
Oxygen regime	1	0.991	560.70	19	92	< 0.001
Genotype × oxygen regime	27	9.511	4.08	513	2090	< 0.001
Residual	110					

*Pillai’s trace with its approximate F statistic and numerator and denominator degrees of freedom; terms as in* Table 4.

### Pearson’s correlation analysis

Pearson’s correlation analysis revealed several strong and significant relationships among the measured traits (Fig 3 and Fig 4). IY was positively correlated with RL (RL1, r = 0.31***), RSA (RSA1, r = 0.23**), LA (LA1, r = 0.49***), and SDW after recovery (SDW2, r = 0.40***), and negatively with RD under hypoxia (RD1, r = −0.30***). RSA was also positively associated with LA and biomass traits (RSA1–LA1, r = 0.49***; RSA1–SDW1, r = 0.45***), whereas RD1 showed negative correlations with LA1, SDW1, and TDW1 (r = −0.38***, −0.40***, and −0.41***). As expected, SDW and TDW were strongly correlated at both sampling times (r = 0.995*** before recovery and 0.997*** after recovery). In the PCA of the 56 genotype × regime means (Fig 4), PC1 (48.55% of the variance) largely separated the hypoxia means (negative scores) from the normoxia means (positive scores); all traits except RD1 (negative loading) and RD2 (loading near zero) loaded positively on PC1, whereas PC2 (9.80%) contrasted the root traits at the end of the treatment (RSA1, RL1) with the biomass traits after recovery (SDW2, RDW2, TDW2).

**Fig 3:**
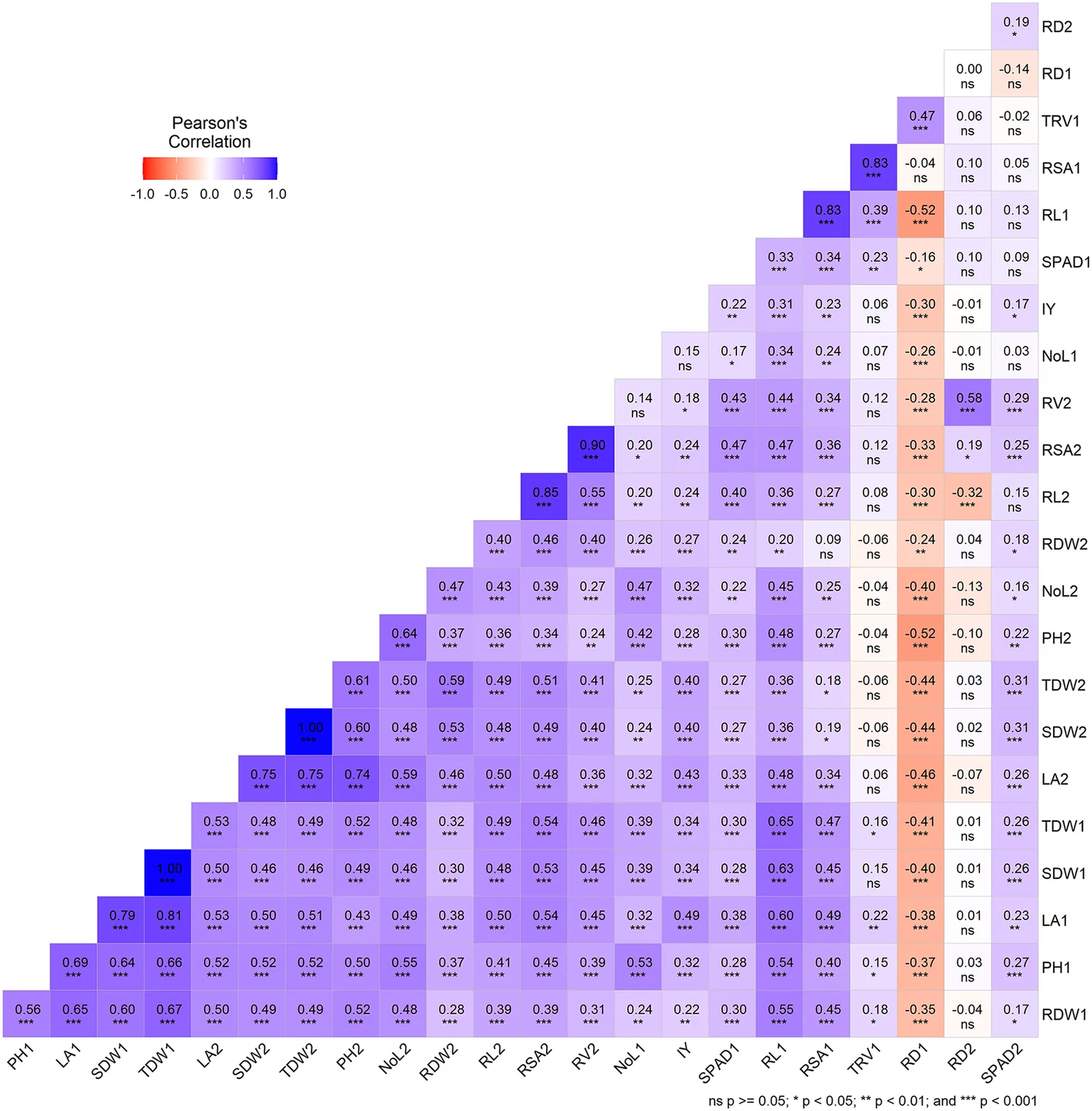
Pearson correlation matrix of 22 root, growth, physiological, and biomass traits and individual seed yield (IY) of 28 quinoa genotypes, computed over the 168 observations of both oxygen regimes (28 genotypes × 2 regimes × 3 replicates). Each cell gives the correlation coefficient (r) and its significance (ns, p ≥ 0.05; *, p < 0.05; **, p < 0.01; ***, p < 0.001); the fill color scales from blue (r = 1) through white (r = 0) to red (r = −1). Trait codes: RL, root length; RSA, root surface area; TRV1 and RV2, root volume; RD, root diameter; PH, plant height; NoL, number of leaves on the main stem; LA, leaf area; SPAD, SPAD chlorophyll value; SDW, RDW, and TDW, shoot, root, and total dry weight; suffix 1, end of the 14-day hypoxia treatment; suffix 2, after 14 days of recovery.

**Fig 4:**
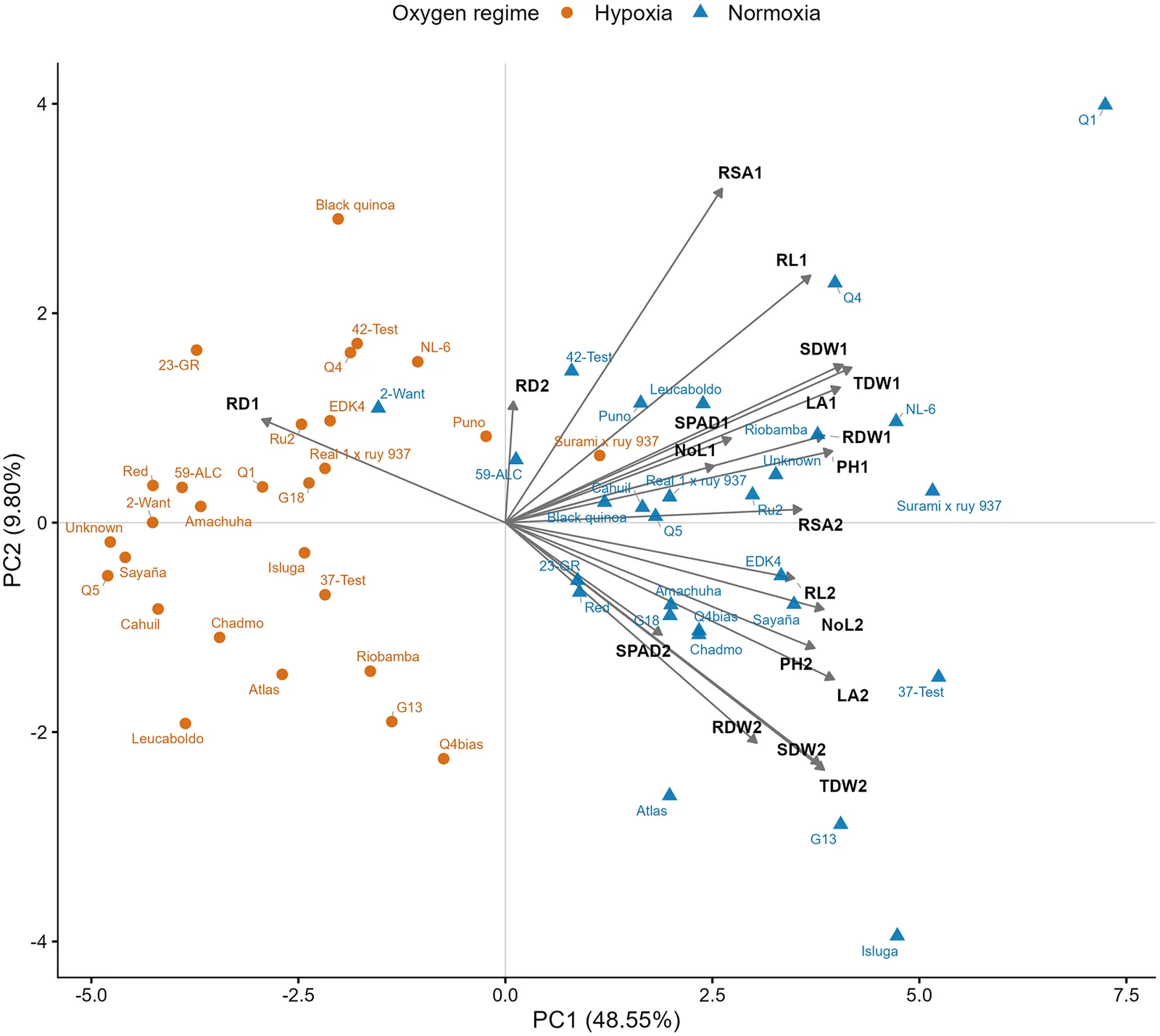
Principal component analysis (PCA) biplot of 20 root, growth, physiological, and biomass traits of 28 quinoa genotypes under hypoxia and normoxia. The PCA was computed on genotype × oxygen-regime mean values (28 genotypes × 2 regimes = 56 observations) after standardizing each trait to zero mean and unit variance. Axes are the first two principal components with the percentage of total variance explained in parentheses (PC1 = 48.55%, PC2 = 9.80%). Gray arrows with bold black labels are the trait loadings: arrow direction shows how the trait relates to PC1 and PC2, arrow length is proportional to the trait’s contribution to the two components, arrows in similar directions indicate positively correlated traits, and arrows in opposite directions indicate negatively correlated traits. Points are the genotype × regime mean scores: vermilion circles are hypoxia and blue triangles are normoxia, each labeled with the genotype name in the same color, so every genotype appears once per regime. Trait codes: RL, root length; RSA, root surface area; RD, root diameter; PH, plant height; NoL, number of leaves on the main stem; LA, leaf area; SPAD, SPAD chlorophyll value; SDW, RDW, and TDW, shoot, root, and total dry weight; suffix 1, end of the 14-day hypoxia treatment; suffix 2, after 14 days of recovery.

### Hierarchical cluster analysis

The hierarchical cluster analysis grouped the 28 quinoa genotypes into three distinct clusters (Fig 5). Cluster I consisted of Red, 59-ALC, Leucaboldo, Chadmo, Riobamba, 37-Test, Cahuil, Q1, Sayaña, Amachuha, Unknown, and Q5. Cluster II included G13, 2-Want, Q4bias, Surami × ruy 937, 42-Test, and Puno. Cluster III comprised 23-GR, Isluga, NL-6, Q4, Ru2, EDK4, G18, Atlas, Black quinoa, and Real 1 × ruy 937. The separation of genotypes into three major clusters indicates substantial variation in multi-trait response profiles among the evaluated germplasm.

**Fig 5:**
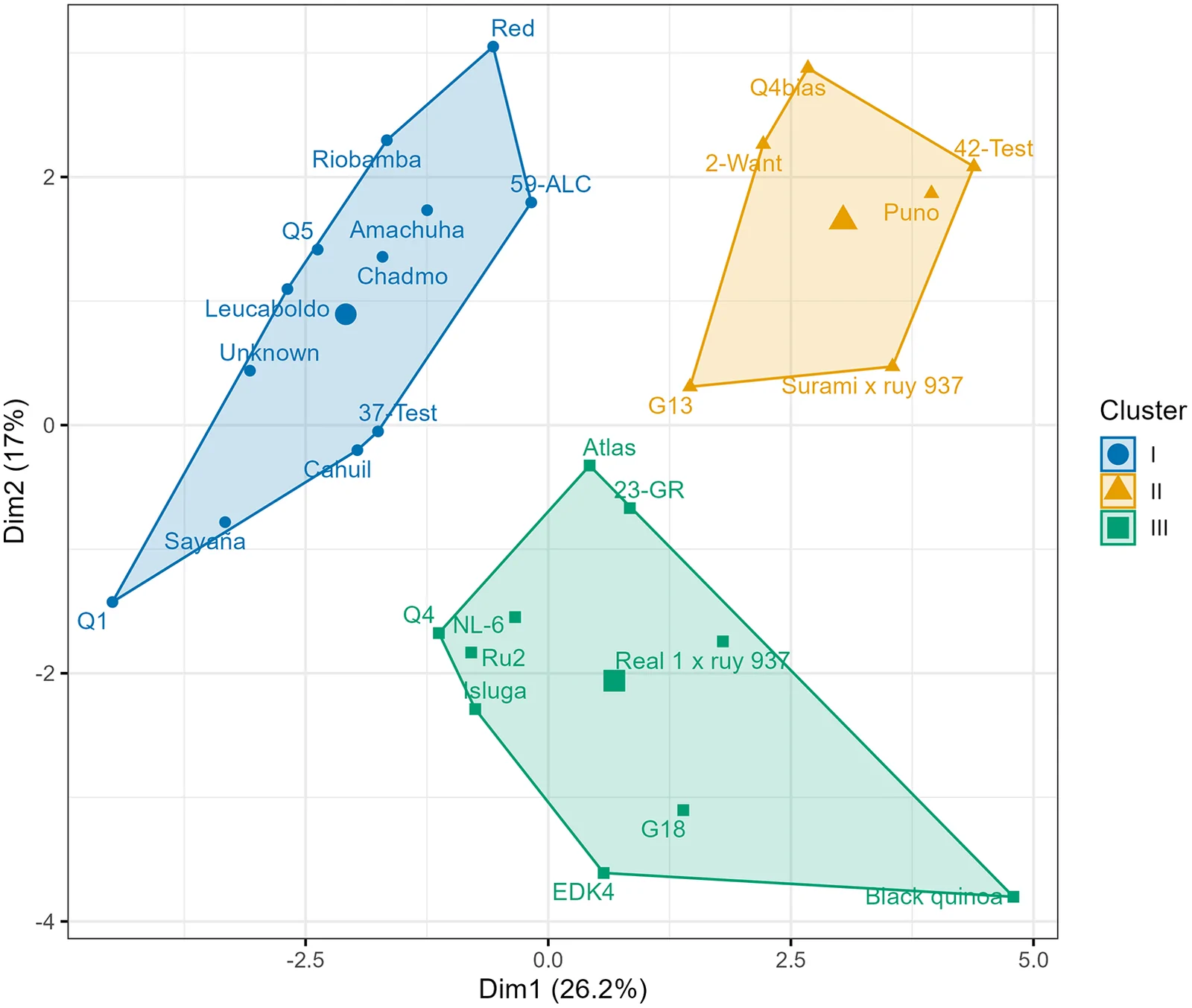
Hierarchical clustering on principal components (HCPC) of the 28 quinoa genotypes based on their hypoxia tolerance indices (HTI = hypoxia mean / normoxia mean of each genotype) for the 22 measured traits. Points are the genotype scores on the first two principal components of the standardized HTI matrix (Dim1 = 26.2%, Dim2 = 17.0% of the variance); color, symbol, and shaded convex hull mark the three clusters (I, blue circles; II, orange triangles; III, green squares), and the large symbol is the cluster centroid. The clusters are those obtained by cutting the hierarchical tree (Ward’s criterion on the principal components) into three groups, followed by the k-means consolidation step of HCPC; cluster membership is listed in the Results.

### Modelings of quinoa individual seed yield trait

Predictive accuracy for genotypes excluded from training was low for all nine models, and none exceeded the regime-mean reference (Fig 6; Table 6). Under leave-one-genotype-out validation the best model, LASSO, reached R^2^ = 0.125 (r = 0.36) against 0.185 for the reference; RF reached 0.105. Withholding whole genotype clusters gave R^2^ ≤ 0.09 (reference 0.19), and training on one ecotype and testing on the other gave pooled R^2^ ≤ 0.05 (reference 0.23). Within a regime, the correlation between observed and predicted genotype-mean yields of withheld genotypes was negative for every model in each of the three designs (highest r = −0.01). By contrast, a random split of individual observations, in which every test-set genotype also appeared in the training set, gave R^2^ = 0.32 for RF and 0.25 for KNN.

**Fig 6:**
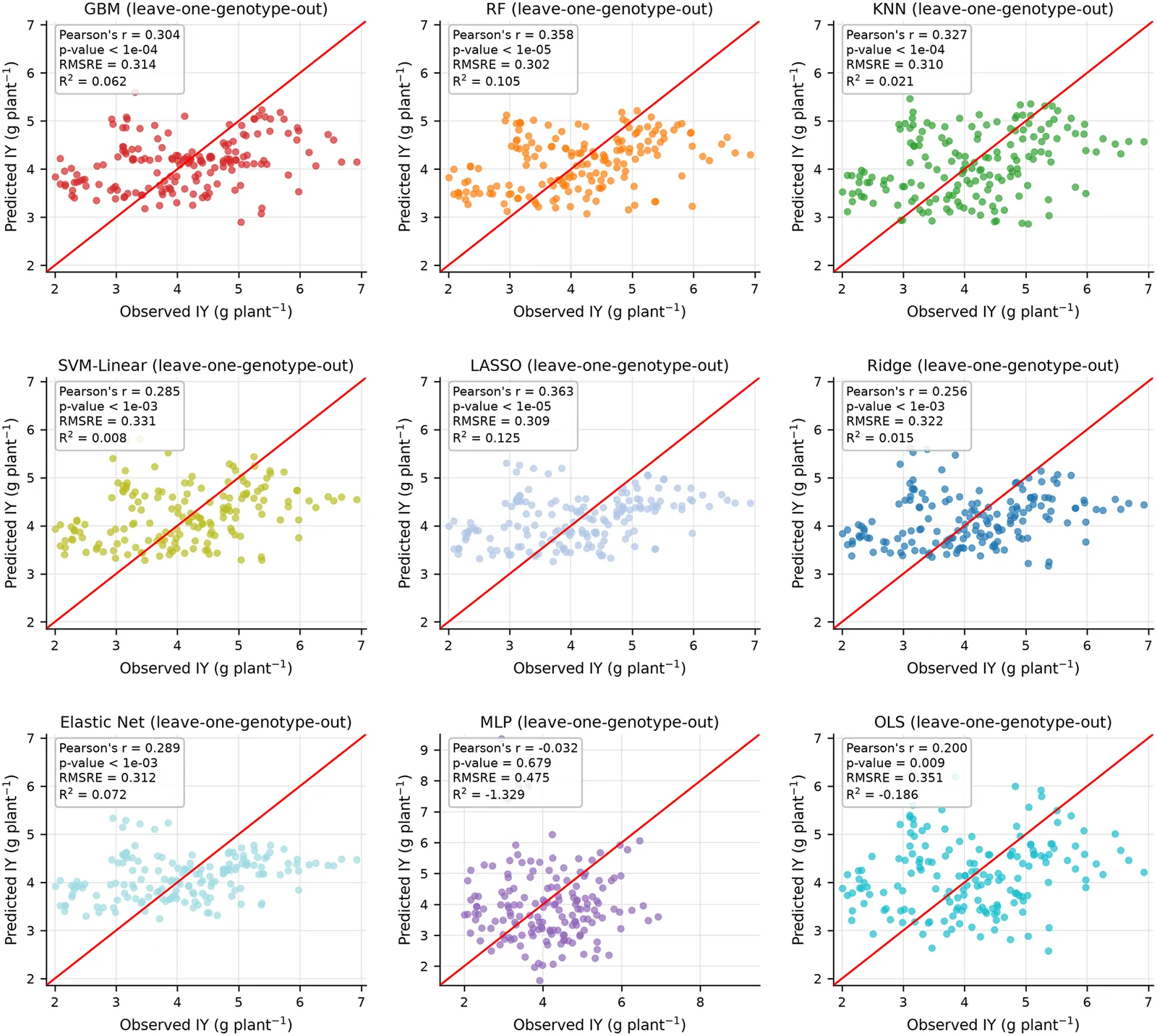
Observed versus predicted individual seed yield (IY, g plant ^−1^) of withheld genotypes for nine machine-learning models under leave-one-genotype-out validation. Each panel shows the 168 held-out predictions (28 genotypes × 2 oxygen regimes × 3 replicates) of one model, each obtained from a model trained on the other 27 genotypes; the red line is the 1:1 line, and the inset gives Pearson’s r with its p-value, the root mean square relative error (RMSRE), and R^2^ of the held-out predictions. Model abbreviations: GBM, gradient boosting machine; RF, random forest; KNN, k-nearest neighbors; SVM-Linear, support vector machine with a linear kernel; LASSO, least absolute shrinkage and selection operator; MLP, multilayer perceptron; OLS, ordinary least squares.

**Table 6:** Predictive accuracy of nine machine-learning models for individual seed yield under four validation designs, compared with a no-trait reference predictor.

Model	Leave-one-genotype-out R² (r)	Leave-one-cluster-out R²	Cross-ecotype R² (r)	Random observation split R²
Regime mean of the training observations (no traits)	0.185 (0.433)	0.189	0.231 (0.481)	0.156
LASSO	0.125 (0.363)	0.088	−0.003 (−0.032)	0.159
RF	0.105 (0.358)	0.063	0.036 (0.273)	0.324
Elastic Net	0.072 (0.289)	0.070	0.033 (0.220)	0.062
GBM	0.062 (0.304)	0.088	−0.089 (0.094)	0.175
KNN	0.021 (0.327)	−0.040	−0.103 (0.199)	0.250
Ridge	0.015 (0.256)	0.030	0.031 (0.280)	0.161
SVM-Linear	0.008 (0.285)	−0.020	0.047 (0.311)	0.119
OLS	−0.186 (0.200)	−0.243	−0.404 (0.258)	0.082
MLP	−1.329 (−0.032)	−1.191	−1.143 (0.083)	−0.322

*Values are R^2^ of the held-out predictions, with Pearson’s r in parentheses where given. Leave-one-genotype-out: each of the 28 genotypes withheld in turn (168 predictions); leave-one-cluster-out: each of the three clusters of* Fig 5 *withheld in turn (168 predictions); cross-ecotype: models trained on the 13 highland genotypes and tested on the 15 lowland genotypes and vice versa, pooled (168 predictions); random observation split: 80/20 split of the 168 observations, in which every test genotype is also present in training (34 predictions). The reference predictor is the mean yield of the training observations under the same oxygen regime; models are ordered by leave-one-genotype-out R^2^. Model abbreviations as in Methods.*

## Discussion

Under waterlogging conditions, hypoxia develops as O_2_ in the root zone is rapidly depleted due to restricted gas diffusion and continuous consumption by roots and soil microorganisms [28]. O_2_ deficiency limits aerobic respiration, resulting in reduced ATP production and a shift toward anaerobic metabolism [29]. Although this metabolic adjustment supports short-term survival, prolonged hypoxia leads to the accumulation of toxic fermentation products such as ethanol and acetaldehyde, which impair root function and plant growth [30]. Consequently, hypoxia is widely recognized as a major constraint to crop growth, biomass accumulation, and yield [29, 31]. The results of this study are consistent with these observations and demonstrate that hypoxia markedly affected the growth and development of quinoa. Across genotypes, hypoxia reduced RL, PH, NoL, LA, RDW, and SDW (Table 3) and IY (Fig 2). In contrast, SPAD values were only slightly affected, whereas RD increased under hypoxia, suggesting a morphological adjustment to hypoxia (Table 3). Similar responses have been reported in quinoa, where significant reductions in biomass and chlorophyll content were observed in the cultivar ‘Sajama’ under waterlogged conditions [32], while reduced leaf growth was reported for the cultivar ‘BRS Piabiru’ under excessive soil moisture [33].

Root traits were sensitive to hypoxia (Table 3; S1 Table). RL showed the greatest reduction among root characteristics at the end of the treatment (mean HTI = 0.61) and, like RSA, remained about 30% lower after recovery (HTI = 0.70). RD, by contrast, increased under hypoxia. Similar responses have been documented in quinoa and other crop species exposed to hypoxia, where inhibition of root elongation and branching is often accompanied by root thickening [9,10,34–36]. The increase in RD observed in this study may represent a morphological response to stress; however, correlation analysis revealed negative associations between RD and LA as well as biomass-related traits, indicating that root thickening did not contribute to improved plant performance under hypoxia (Fig 3 and Fig 4). In contrast, maintenance of RL and RSA was closely associated with superior growth and productivity, suggesting that these traits may serve as reliable indicators of hypoxia tolerance in quinoa.

Previous studies have reported substantial genotypic variation in root responses of upland crops such as soybean [10], wheat [11], barley [12] and sweet potato [37] to hypoxia. Similarly, the present study revealed considerable variation among quinoa genotypes in their adaptive responses to hypoxia. Genotypes such as G18, EDK4, and Puno maintained relatively high RL under stress (RL1 HTI = 0.75–0.94), whereas Puno, NL-6, and 2-Want retained RDW almost fully (RDW1 HTI = 0.92–0.96; S1 and S4 Tables). More importantly, Isluga, EDK4, and G18 (RL1 HTI = 0.75, 0.75, and 0.94) combined favorable root characteristics with high individual yield under hypoxia (Fig 2), indicating that the maintenance of root growth contributes to productivity under hypoxia. In addition, Amachuha, Isluga, EDK4, Chadmo, and 42-Test achieved the highest individual yields under hypoxia, followed by G13, Ru2, and G18, whereas Q4, Atlas, Sayaña, Cahuil, and Red yielded least (Fig 2). These findings demonstrate substantial genetic variation for hypoxia tolerance in quinoa and identify several promising genotypes that could serve as valuable genetic resources for breeding programs targeting waterlogging-prone environments.

The clustering pattern also reflected the genetic background and stress-adaptive characteristics of the evaluated genotypes. The clusters followed ecotype: Cluster I contained 10 of the 13 highland genotypes, and Clusters II and III 13 of the 15 lowland genotypes (Fisher’s exact test, p = 0.002), consistent with the eco-geographical differentiation of quinoa populations [26,27,38,39]. Similar clustering patterns have been reported under salinity and drought, where tolerant genotypes such as Atlas and 2-Want maintained superior root growth and stress performance [17,25], while our previous study under contrasting water regimes identified G18, Q1, and Q4 as stable, high-performing genotypes across drought and waterlogging environments [13,19]. The repeated identification of G18 as a superior genotype under drought, waterlogging, and hypoxia suggests broad tolerance to multiple abiotic stresses. Accordingly, G18 in this study, together with Isluga, EDK4, 42-Test, and G13, which rank among the eight highest-yielding genotypes under hypoxia (Fig 2) and fall in Clusters II and III of the trait-response clustering (Fig 5), are promising genetic resources for breeding quinoa cultivars with improved hypoxia tolerance.

Trait-based prediction of individual yield did not transfer to genotypes excluded from training (Fig 6; Table 6). Under leave-one-genotype-out, leave-one-cluster-out, and cross-ecotype validation, no model predicted the yield of a withheld genotype better than the mean yield of the training genotypes under the same oxygen regime, and within a regime the predictions did not reproduce the genotype ranking. The pooled correlations under leave-one-genotype-out validation (r = 0.20–0.36 for every model except MLP; Table 6) therefore reflect the hypoxia-versus-normoxia difference in yield, which the traits also reflect, and the higher accuracy of the random split of observations is consistent with models recognizing genotypes seen in training. With 28 genotypes, the models learned genotype-specific trait combinations. The pooled trait–yield correlations (Fig 3), which are dominated by the regime effect, already carry the transferable signal (root length, root surface area, leaf area, and biomass positively, root diameter negatively associated with yield). Because no genetic marker information was included, these models cannot by themselves resolve the specific physiological pathways or loci underlying variation in individual yield under hypoxia. These results do not support using the present models to rank untested genotypes. They indicate that a transferable phenotype-based yield predictor for quinoa under hypoxia would require a larger and genetically broader training set.

## Limitations

This study evaluated 28 genotypes under two researcher-imposed oxygen regimes (normoxia and hypoxia) rather than a multi-environment trial; the genotype × oxygen-regime results reported here should therefore be read as genotype-by-treatment responses under these two conditions, not as evidence of broader environmental adaptation. Genotypes were assigned to the highland or the lowland ecotype from their origin rather than from molecular markers, so genetic relatedness among genotypes could be controlled only at the level of ecotype and of the trait-response clusters. The machine-learning results are exploratory: with 28 genotypes, no model predicted the yield of genotypes outside the training set, or across ecotypes, better than the mean yield of the training genotypes under the same oxygen regime, and the models should not be used to rank untested germplasm.

## Conclusion

Hypoxia significantly affected the growth, root development, biomass accumulation, and yield of quinoa, with substantial variation among genotypes. RL and RSA were positively associated with IY, whereas RD showed negative relationships with several growth traits, highlighting the importance of maintaining root development under hypoxia. Genotype, oxygen regime, and their interaction each explained a substantial share of multivariate phenotypic variation, and hierarchical clustering on hypoxia tolerance indices distinguished three trait-profile groups; Amachuha and Isluga produced the highest individual yields under hypoxia. Under leave-one-genotype-out, leave-one-cluster-out, and cross-ecotype validation, none of the nine machine-learning models predicted the yield of genotypes excluded from training better than the regime mean, so the trait-based models remain exploratory and do not yet transfer to untested germplasm. These findings describe genotype responses to two controlled oxygen regimes rather than to a sampled population of field environments; validating the promising genotypes identified here under field waterlogging conditions across multiple seasons or locations, and investigating the physiological, anatomical, and genetic mechanisms underlying hypoxia tolerance, are necessary next steps.

## Supporting information

S1 File Analysis code and data (S1_Analysis_Code). Corrected phenotypic dataset, the dataset as originally submitted, the cell-by-cell changelog, the ecotype assignment, and all R and Python scripts used to generate the tables and figures.
(ZIP)

S1 Table Root traits of 28 quinoa genotypes under normoxia and hypoxia and their hypoxia tolerance index (HTI). RL1, RSA1, and RD1: root length (cm), root surface area (cm^2^), and root diameter (mm) at the end of the 14-day hypoxia treatment (30 days after sowing, DAS); RL2, RSA2, and RD2: the same traits after 14 days of recovery (44 DAS). Values are means of three replicates; HTI = hypoxia mean / normoxia mean of each genotype.
(DOCX)

S2 Table Plant height (PH, cm) and number of leaves on the main stem (NoL) of 28 quinoa genotypes under normoxia and hypoxia and their hypoxia tolerance index (HTI). PH1 and NoL1: end of the 14-day hypoxia treatment (30 DAS); PH2 and NoL2: after 14 days of recovery (44 DAS). Values are means of three replicates; HTI = hypoxia mean / normoxia mean of each genotype.
(DOCX)

S3 Table Leaf area (LA, cm²) and SPAD value of 28 quinoa genotypes under normoxia and hypoxia and their hypoxia tolerance index (HTI). LA1 and SPAD1: end of the 14-day hypoxia treatment (30 DAS); LA2 and SPAD2: after 14 days of recovery (44 DAS). Values are means of three replicates; HTI = hypoxia mean / normoxia mean of each genotype.
(DOCX)

S4 Table Shoot dry weight (SDW, g plant ^−1^) and root dry weight (RDW, g plant ^−^^1^) of 28 quinoa genotypes under normoxia and hypoxia and their hypoxia tolerance index (HTI). SDW1 and RDW1: end of the 14-day hypoxia treatment (30 DAS); SDW2 and RDW2: after 14 days of recovery (44 DAS). Values are means of three replicates; HTI = hypoxia mean / normoxia mean of each genotype.
(DOCX)
