# Supplementary material for: Genotypic variation and root traits associated with hypoxia tolerance in quinoa (Chenopodium quinoa Willd.)
Source: PLoS One. 2026 Nov 3;21(11):e0361654. doi: 10.1371/journal.pone.0361654 (PMC13730327; doi:10.1371/journal.pone.0361654)
Supplement: Supplementary file 1 [file pone.0361654.s006.pdf]

Integrative Phenotyping, Multivariate Analyses, and Machine Learning Prediction for Quinoa (*Chenopodium quinoa* Willd.) under Hypoxia

The manuscript addresses an important topic and includes a valuable dataset with potential implications for quinoa breeding under hypoxia stress. However, substantial revisions are required to improve methodological transparency, strengthen the organization of the Results and Discussion sections, clarify the experimental design, and enhance the reproducibility of the statistical and machine-learning analyses. These improvements would considerably increase the scientific quality and impact of the manuscript.

Abstract

1. The abbreviation RSA is introduced in the sentence "Correlation analysis revealed that RL and RSA were positively associated with IY" without being previously defined. Please provide the full term at its first occurrence before using the abbreviation.

2. In the phrase "machine-learning approaches, particularly KNN, RF, SVM, and GBM," the abbreviations are introduced without first stating their full names. Please spell out the full names of these algorithms at their first mention, followed by the corresponding abbreviations in parentheses.

3. The abstract should present the main findings using quantitative results rather than only qualitative statements. Please include key numerical values (e.g., percentage reductions, correlation coefficients, model performance metrics, or explained variance) to highlight the principal outcomes of the study.

4. Please revise the keywords. They should be arranged in alphabetical order and should not duplicate terms already included in the title. Consider selecting complementary indexing terms to improve the discoverability of the manuscript.

Introduction

1. The abbreviation GGE is introduced without being defined. Please spell out the full term at its first occurrence. In addition, the submitted manuscript does not include continuous line numbering, which makes the review process unnecessarily difficult. Please include line numbers throughout the manuscript.

2. The following sentence should be completely rewritten for clarity, grammar, and scientific style:

"By integrating physiological, agronomic, and data-driven approaches, this study provides a comprehensive framework for identifying hypoxia-tolerant quinoa genotypes and improving selection strategies of breeding program for waterlogging tolerance in quinoa plants."

The current wording is based using AI.

Materials and Methods

Plant material

1. The Plant Material section requires substantially more detail. Please describe the origin of the germplasm, relevant characteristics of each genotype, the rationale for their selection, and whether any genotype was included as a reference or check. The current description is insufficient.

Experimental method

2. Please verify the expression "moist tessue." It appears to contain a typographical error and should probably read "moist tissue paper."

3. The sentence: "Then, the 14-old-seedlings were grown for 14 days..."

contains grammatical errors. It should be revised (e.g., "Then, 14-day-old seedlings were grown for an additional 14 days...").

4. Figure 1. The figure panels should follow the chronological order of the experimental procedure described in the manuscript. Based on the methodology, the logical sequence appears to be panel C, followed by B, and finally A. Furthermore, the figure legend is too brief and should provide a detailed explanation of each panel so that the figure can be understood independently of the main text.

5. Please clearly define normoxia and hypoxia within the methodology when these treatments are first introduced. The experimental conditions should be explicitly described for readers unfamiliar with these terms.

6. Experimental design. It is unclear how the experiment was arranged as a randomized complete block design (RCBD) given that two contrasting oxygen conditions (normoxia and hypoxia) were imposed. Please clarify how the experimental units were randomized and blocked under these conditions. In addition, define DO at its first occurrence and explain how oxigen concentration was monitored and maintained throughout the experiment.

7. The Experimental method section should be reorganized into clearer subsections with more detailed methodological descriptions. Several procedures are currently described too briefly to ensure reproducibility.

8. Please report the measurement units for all evaluated variables. In addition, the authors are encouraged to indicate the crop developmental stage at which the experiment was initiated using the BBCH scale.

9. The sentence: "SD was measured at a 2 cm height..."introduces SD without definition. Please verify all abbreviations throughout the manuscript and ensure that each term is written in full before its abbreviation is used.

Data analysis

10. Please indicate the version of R used in the manuscript. In addition, provide the version number for every R package used and cite the software appropriately.

11. The Data analysis section should be reorganized into shorter paragraphs with clearer subsectioning. The current presentation consists of excessively long paragraphs that reduce readability. Furthermore, please include appropriate methodological references for the statistical indices and machine-learning algorithms employed.

12. Please clarify what constitutes the environments in the genotype × environment (G×E) analysis. The statistical model used for the G×E analysis should also be explicitly presented within the methodology.

13. It is strongly recommended that the complete analysis scripts (e.g., R and Python scripts) be provided as supplementary material. Given the use of multiple statistical models and machine-learning approaches, sharing the analytical workflow would substantially improve the transparency and reproducibility of the study.

Results

1. The Results section should be reorganized according to the objectives of the study. Since each objective addresses a specific scientific question, the presentation of results should follow the same logical structure. In addition, each subsection should begin with a brief introductory sentence explaining the purpose of the analyses presented.

2. Figure 2. The figure should distinguish between comparisons among treatments and comparisons among genotypes by using two different lettering systems (e.g., uppercase and lowercase letters). Please clarify this in both the figure and its legend.

3. The Materials and Methods section does not specify which software package or R package was used to perform the correlation analysis. Please include this information to improve reproducibility.

4. Pearson's correlation analysis. Given the large number of variables included, the Pearson correlation matrix is difficult to interpret and provides limited biological insight. The authors may consider complementing this analysis with a Principal Component Analysis (PCA), which would provide a more integrated visualization of the relationships among genotypes and traits while reducing redundancy among the figures.

5. AMMI and GGE biplot analysis. Because only two environments were evaluated, the first two principal components explain all G+GE variation. This point should be explicitly clarified in the manuscript to avoid confusion among readers regarding the reported 100% explained variation.

6. The statement: "These results demonstrate a strong genotype × environment interaction and indicate that Isluga is a promising genetic resource for breeding quinoa with improved tolerance to hypoxia." is interpretative rather than descriptive. Such biological interpretation should be presented in the Discussion, whereas the Results section should focus on objectively reporting the findings.

7. Hierarchical cluster analysis. The hierarchical clustering analysis provides limited additional biological insight beyond the GGE analysis. The authors may consider integrating a Principal Component Analysis (PCA) to summarize genotype and trait relationships more effectively and reduce redundancy among Figures 3–6.

8. Modeling of quinoa individual seed yield trait. The statement: "Overall, KNN, RF, SVM-Linear, and GBM were the most effective models for predicting IY, indicating that non-linear approaches better captured the variation in the dataset than traditional linear models." is interpretative rather than descriptive. The Results section should report the model performance objectively, whereas the interpretation of why certain algorithms outperformed others should be moved to the Discussion.

9. In general, the Results section is insufficiently developed despite the large number of figures and tables presented. The manuscript currently emphasizes graphical outputs rather than answering the scientific objectives of the study. The section should be reorganized so that each subsection directly addresses one research objective, with figures and tables included only when they meaningfully support the scientific narrative.

Discussion

1. Please include explicit cross-references to the relevant figures and tables throughout the Discussion. Referring to the corresponding results will strengthen the interpretation and improve coherence between the Results and Discussion sections.

2. The Discussion should be reorganized into subsections that correspond to the specific objectives of the study. This structure would improve readability and provide a more logical interpretation of the findings.

3. The manuscript should include a subsection discussing the limitations of the study. A balanced discussion of methodological limitations and potential sources of uncertainty would strengthen the scientific rigor of the manuscript.

Conclusions

1. I am not convinced that this study should be presented as a classical genotype × environment (G×E) interaction study. Based on the experimental design described, the work appears to correspond to a two-factor factorial experiment involving genotype and oxygen treatment rather than a conventional multi-environment trial. The authors should clearly define what they consider to be the "environments" in this context, justify the application of G×E terminology, and provide a more detailed description of the implementation of the randomized complete block design (RCBD), particularly regarding the arrangement of experimental units under the two oxygen conditions.
