# Supplementary material for: Genotypic variation and root traits associated with hypoxia tolerance in quinoa (Chenopodium quinoa Willd.)
Source: PLoS One. 2026 Nov 3;21(11):e0361654. doi: 10.1371/journal.pone.0361654 (PMC13730327; doi:10.1371/journal.pone.0361654)
Supplement: Supplementary file 2 [file pone.0361654.s001.zip › S1_Analysis_Code/outputs/Correlation/Correlation_matrix.pdf]

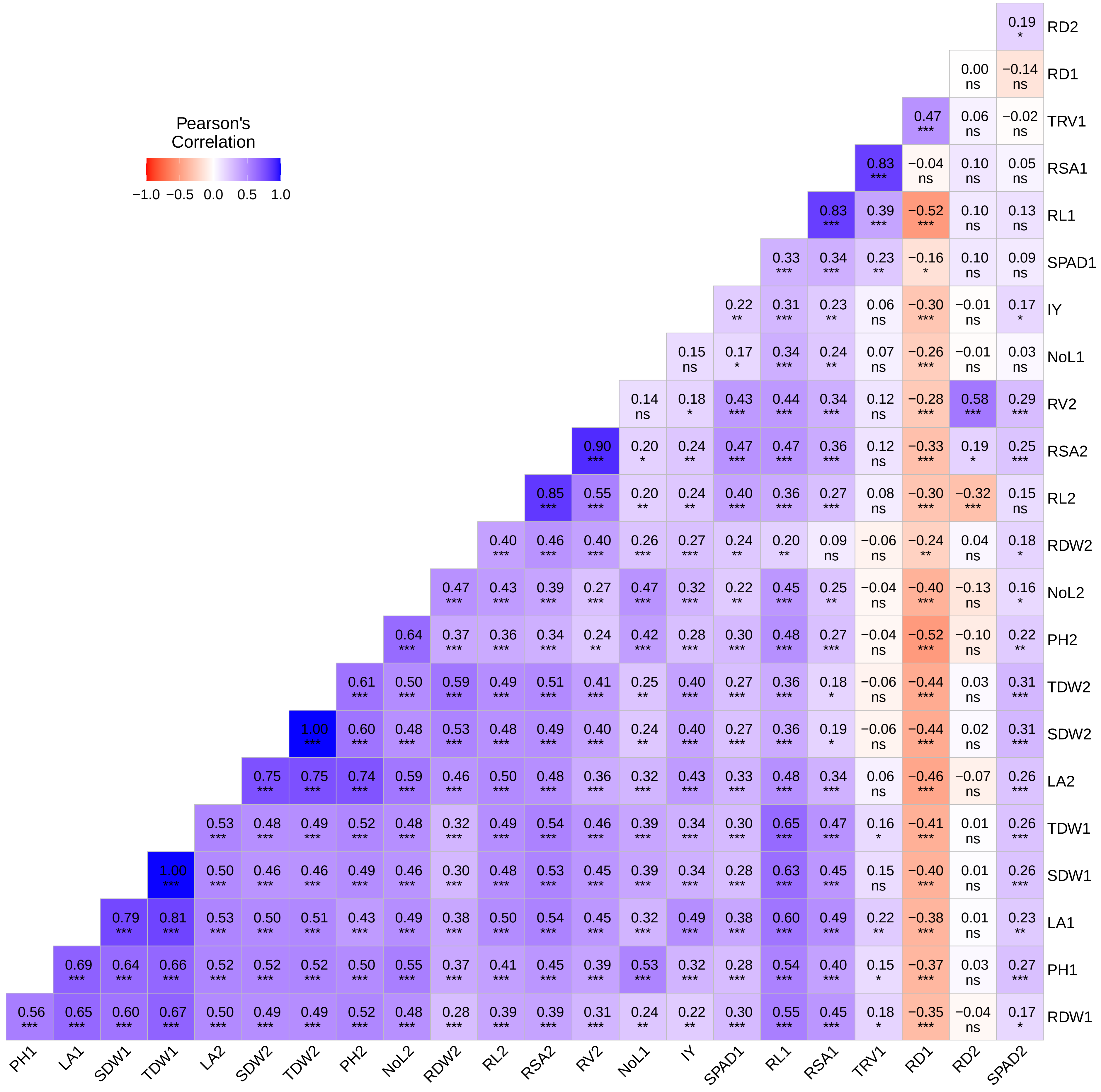

ns p >= 0.05; * p < 0.05; ** p < 0.01; and *** p < 0.001
