# Supplementary material for: Genotypic variation and root traits associated with hypoxia tolerance in quinoa (Chenopodium quinoa Willd.)
Source: PLoS One. 2026 Nov 3;21(11):e0361654. doi: 10.1371/journal.pone.0361654 (PMC13730327; doi:10.1371/journal.pone.0361654)
Supplement: Supplementary file 2 [file pone.0361654.s001.zip › S1_Analysis_Code/outputs/HCPC/HCPC_cluster_map.pdf]

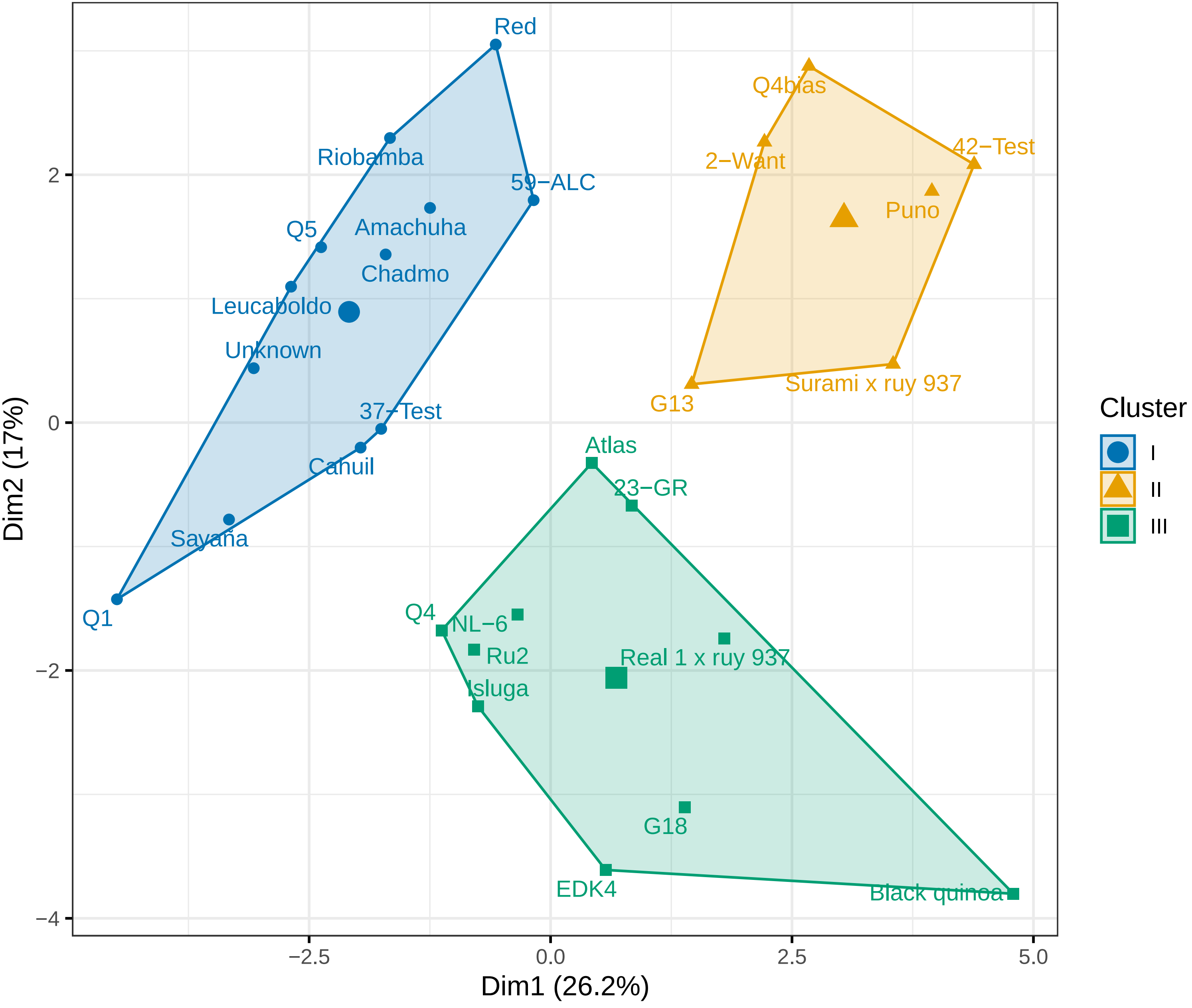
Red
Riobamba
59−ALC
Q5
Amachuha
Chadmo
Leucaboldo
Unknown
37−Test
Cahuil
Sayaña
Q1
Q4bias
2−Want
42−Test
Puno
G13
Surami x ruy 937
Atlas
23−GR
Q4
NL−6
Ru2
Real 1 x ruy 937
Isluga
G18
EDK4
Black quinoa
Dim2 (17%)
Dim1 (26.2%)
2
0
−2
−4
−2.5
0.0
2.5
5.0
Cluster
I
II
III
