# Supplementary material for: Genotypic variation and root traits associated with hypoxia tolerance in quinoa (Chenopodium quinoa Willd.)
Source: PLoS One. 2026 Nov 3;21(11):e0361654. doi: 10.1371/journal.pone.0361654 (PMC13730327; doi:10.1371/journal.pone.0361654)
Supplement: Supplementary file 2 [file pone.0361654.s001.zip › S1_Analysis_Code/outputs/HCPC/HCPC_dendrogram.pdf]

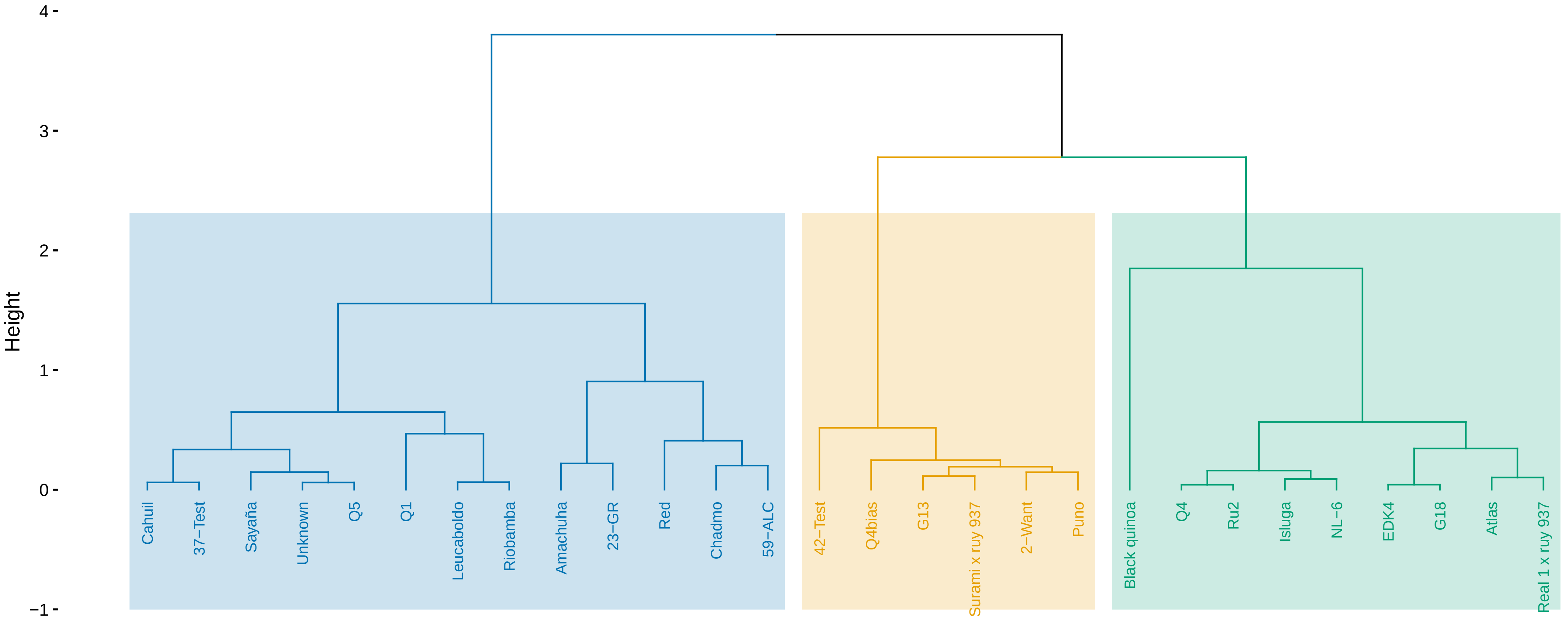

Height
4
3
2
1
0
−1
Cahuil
37−Test
Sayaña
Unknown
Q5
Q1
Leucaboldo
Riobamba
Amachuha
23−GR
Red
Chadmo
59−ALC
42−Test
Q4bias
G13
Surami x ruy 937
2−Want
Puno
Black quinoa
Q4
Ru2
Isluga
NL−6
EDK4
G18
Atlas
Real 1 x ruy 937
