# Supplementary material for: Genotypic variation and root traits associated with hypoxia tolerance in quinoa (Chenopodium quinoa Willd.)
Source: PLoS One. 2026 Nov 3;21(11):e0361654. doi: 10.1371/journal.pone.0361654 (PMC13730327; doi:10.1371/journal.pone.0361654)
Supplement: Supplementary file 2 [file pone.0361654.s001.zip › S1_Analysis_Code/outputs/IY_boxplot/IY_boxplot.pdf]

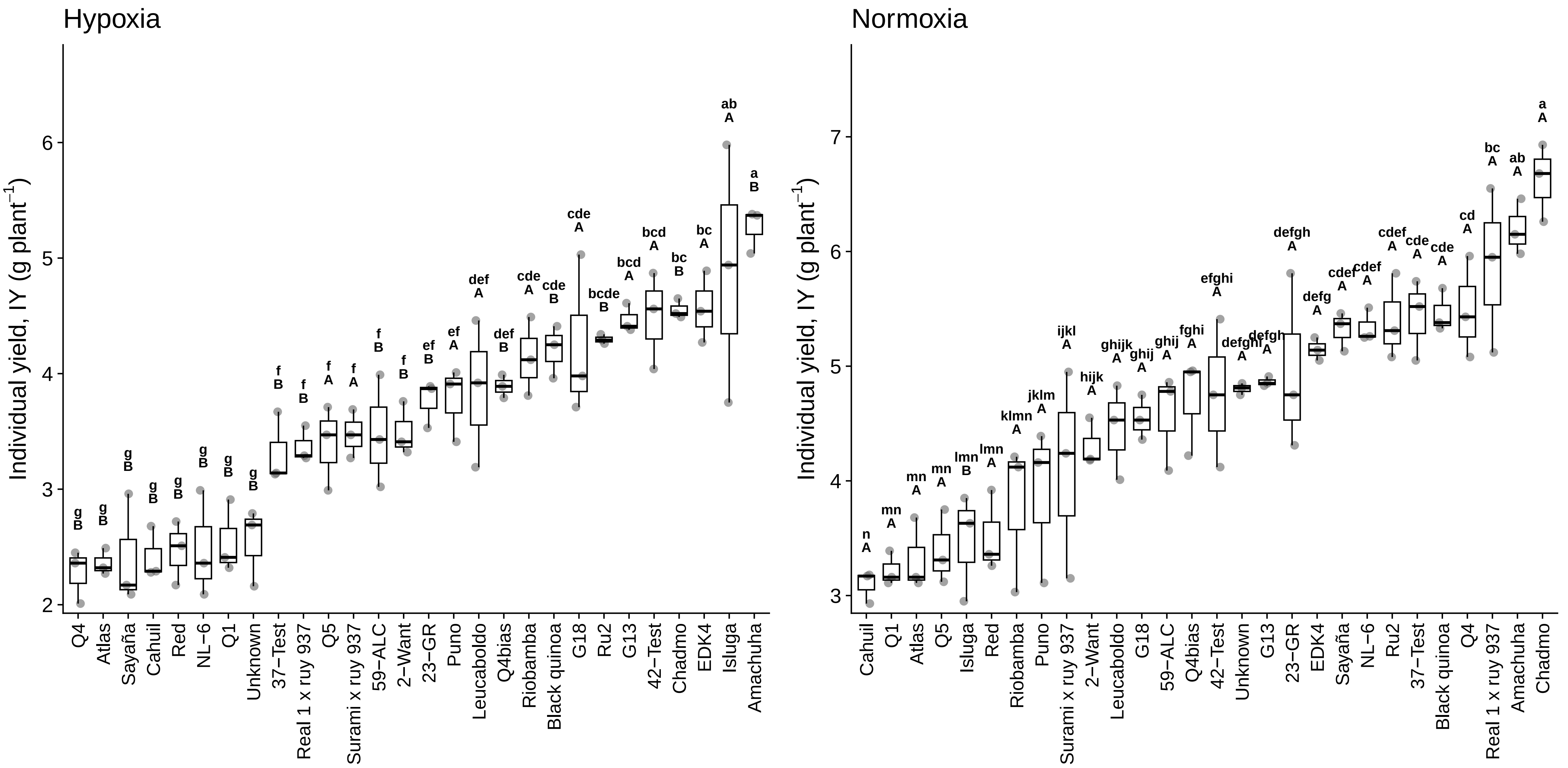

Hypoxia
Normoxia
Individual yield, IY (g plant⁻¹)
Q4
Atlas
Sayaña
Cahuil
Red
NL−6
Q1
Unknown
37−Test
Real 1 x ruy 937
Q5
Surami x ruy 937
59−ALC
2−Want
23−GR
Puno
Leucaboldo
Q4bias
Riobamba
Black quinoa
G18
Ru2
G13
42−Test
Chadmo
EDK4
Isluga
Amachuha
