# Supplementary material for: Genotypic variation and root traits associated with hypoxia tolerance in quinoa (Chenopodium quinoa Willd.)
Source: PLoS One. 2026 Nov 3;21(11):e0361654. doi: 10.1371/journal.pone.0361654 (PMC13730327; doi:10.1371/journal.pone.0361654)
Supplement: Supplementary file 2 [file pone.0361654.s001.zip › S1_Analysis_Code/outputs/MGIDI/MGIDI_bootstrap_selection_frequency.pdf]

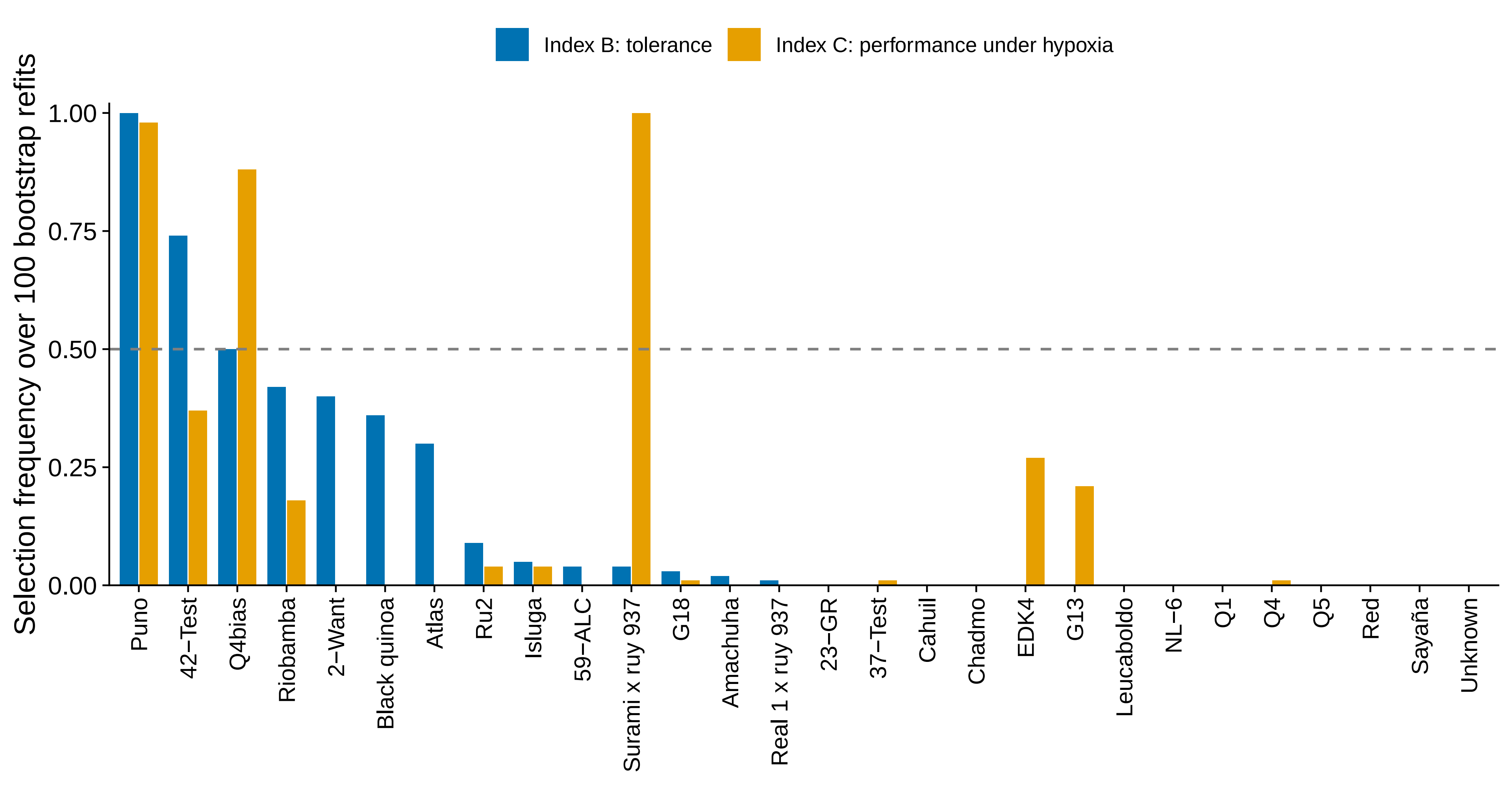

Index B: tolerance
Index C: performance under hypoxia
Selection frequency over 100 bootstrap refits
1.00
0.75
0.50
0.25
0.00
Puno
42−Test
Q4bias
Riobamba
2−Want
Black quinoa
Atlas
Ru2
Isluga
59−ALC
Surami x ruy 937
G18
Amachuha
Real 1 x ruy 937
23−GR
37−Test
Cahuil
Chadmo
EDK4
G13
Leucaboldo
NL−6
Q1
Q4
Q5
Red
Sayaña
Unknown
