# Supplementary material for: Genotypic variation and root traits associated with hypoxia tolerance in quinoa (Chenopodium quinoa Willd.)
Source: PLoS One. 2026 Nov 3;21(11):e0361654. doi: 10.1371/journal.pone.0361654 (PMC13730327; doi:10.1371/journal.pone.0361654)
Supplement: Supplementary file 2 [file pone.0361654.s001.zip › S1_Analysis_Code/outputs/MGIDI/MGIDI_indexB_metan_plot.pdf]

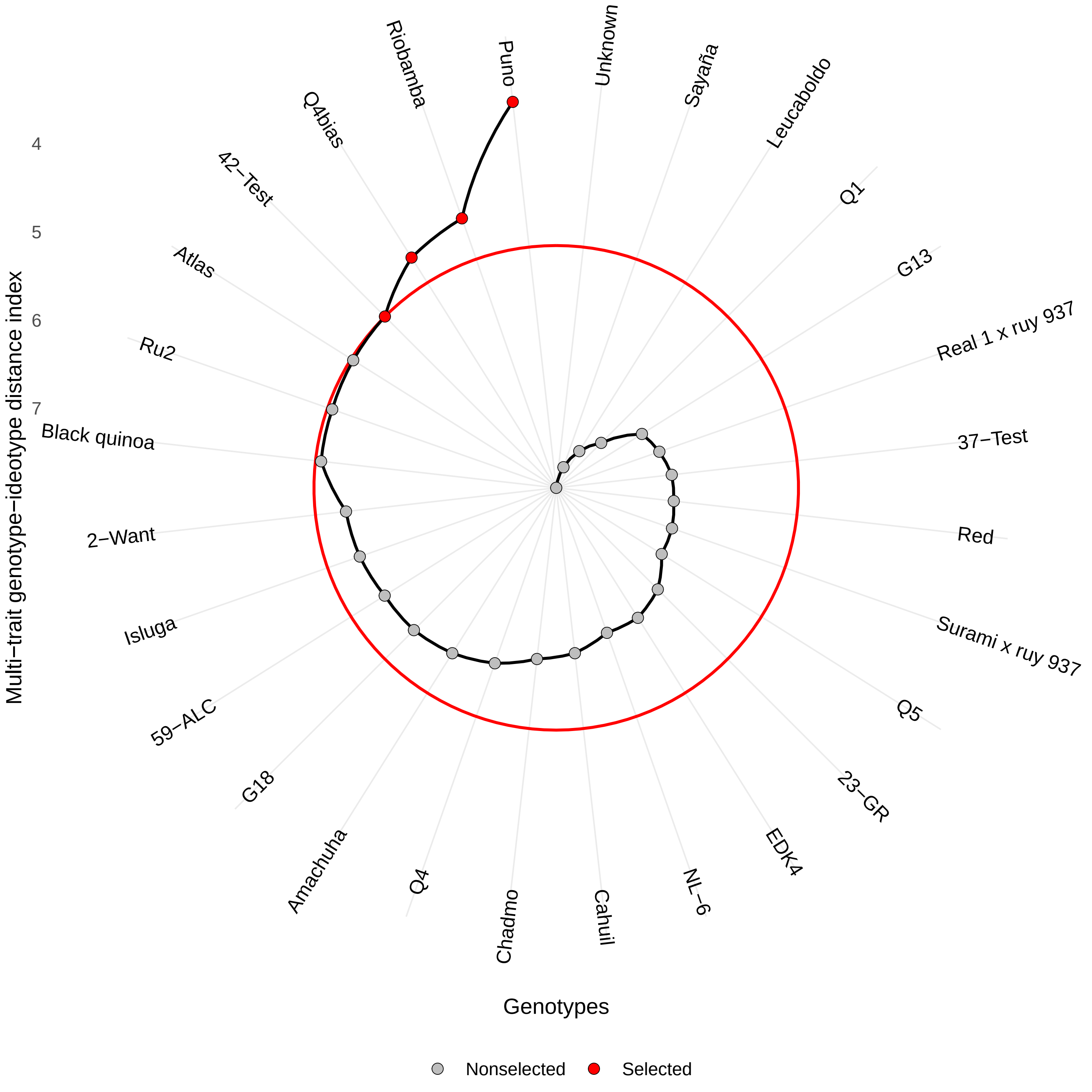

Multi−trait genotype−ideotype distance index
4
5
6
7
Puno
Unknown
Sayaña
Leucaboldo
Q1
G13
Real 1 x ruy 937
37−Test
Red
Surami x ruy 937
Q5
23−GR
EDK4
NL−6
Cahuil
Chadmo
Q4
Amachuha
G18
59−ALC
Isluga
2−Want
Black quinoa
Ru2
Atlas
42−Test
Q4bias
Riobamba
Genotypes
Nonselected
Selected
