# Supplementary material for: Genotypic variation and root traits associated with hypoxia tolerance in quinoa (Chenopodium quinoa Willd.)
Source: PLoS One. 2026 Nov 3;21(11):e0361654. doi: 10.1371/journal.pone.0361654 (PMC13730327; doi:10.1371/journal.pone.0361654)
Supplement: Supplementary file 2 [file pone.0361654.s001.zip › S1_Analysis_Code/outputs/MGIDI/MGIDI_rank_vs_yield_rank.pdf]

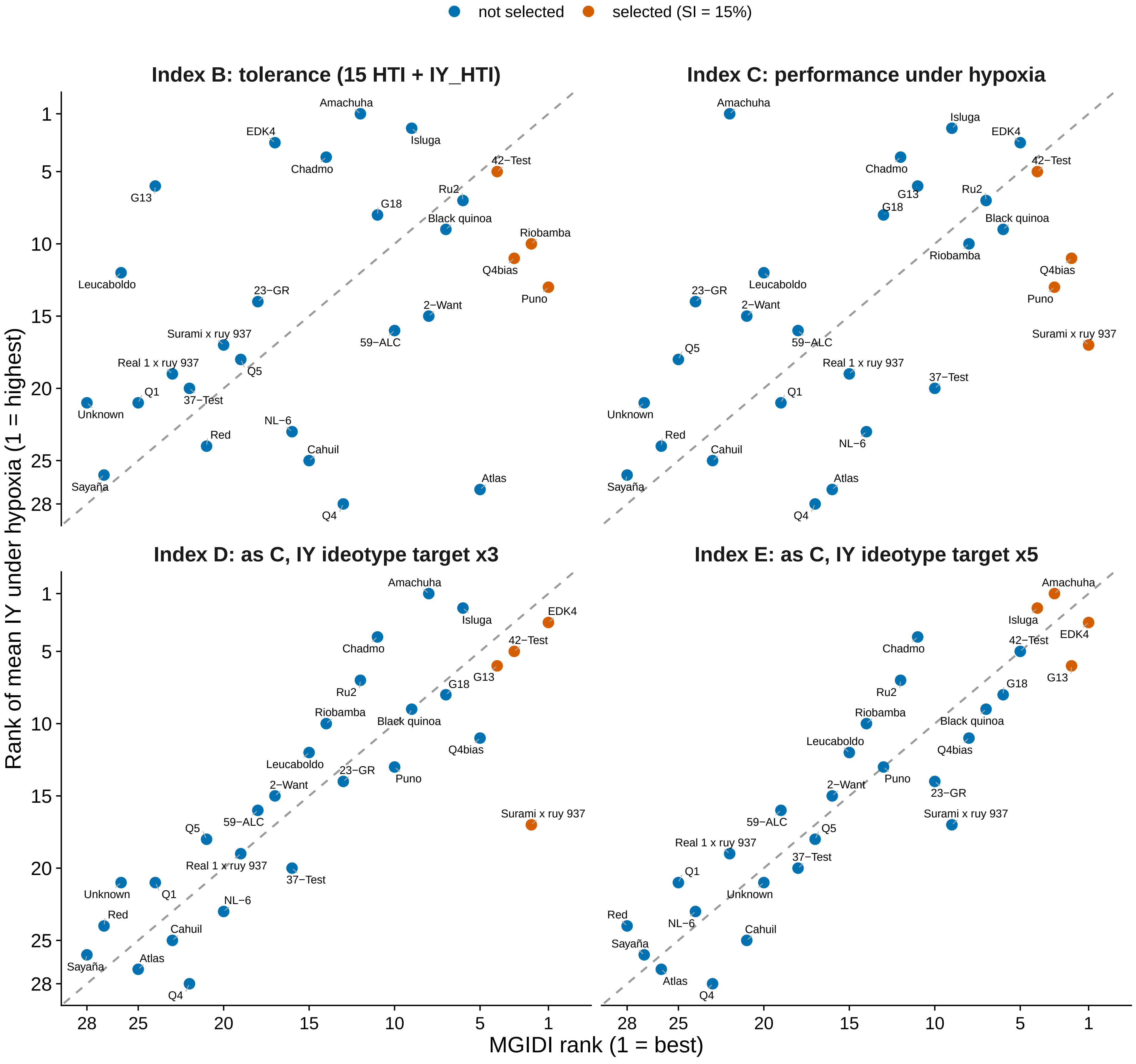

not selected
selected (SI = 15%)
Index B: tolerance (15 HTI + IY_HTI)
Index C: performance under hypoxia
Index D: as C, IY ideotype target x3
Index E: as C, IY ideotype target x5
Rank of mean IY under hypoxia (1 = highest)
MGIDI rank (1 = best)
Amachuha
EDK4
Isluga
Chadmo
42−Test
G13
Ru2
G18
Black quinoa
Riobamba
Q4bias
Puno
Leucaboldo
23−GR
2−Want
59−ALC
Surami x ruy 937
Q5
Real 1 x ruy 937
37−Test
Q1
Unknown
NL−6
Red
Cahuil
Sayaña
Atlas
Q4
