# Supplementary material for: Genotypic variation and root traits associated with hypoxia tolerance in quinoa (Chenopodium quinoa Willd.)
Source: PLoS One. 2026 Nov 3;21(11):e0361654. doi: 10.1371/journal.pone.0361654 (PMC13730327; doi:10.1371/journal.pone.0361654)
Supplement: Supplementary file 2 [file pone.0361654.s001.zip › S1_Analysis_Code/outputs/MGIDI/MGIDI_variance_components_MET.pdf]

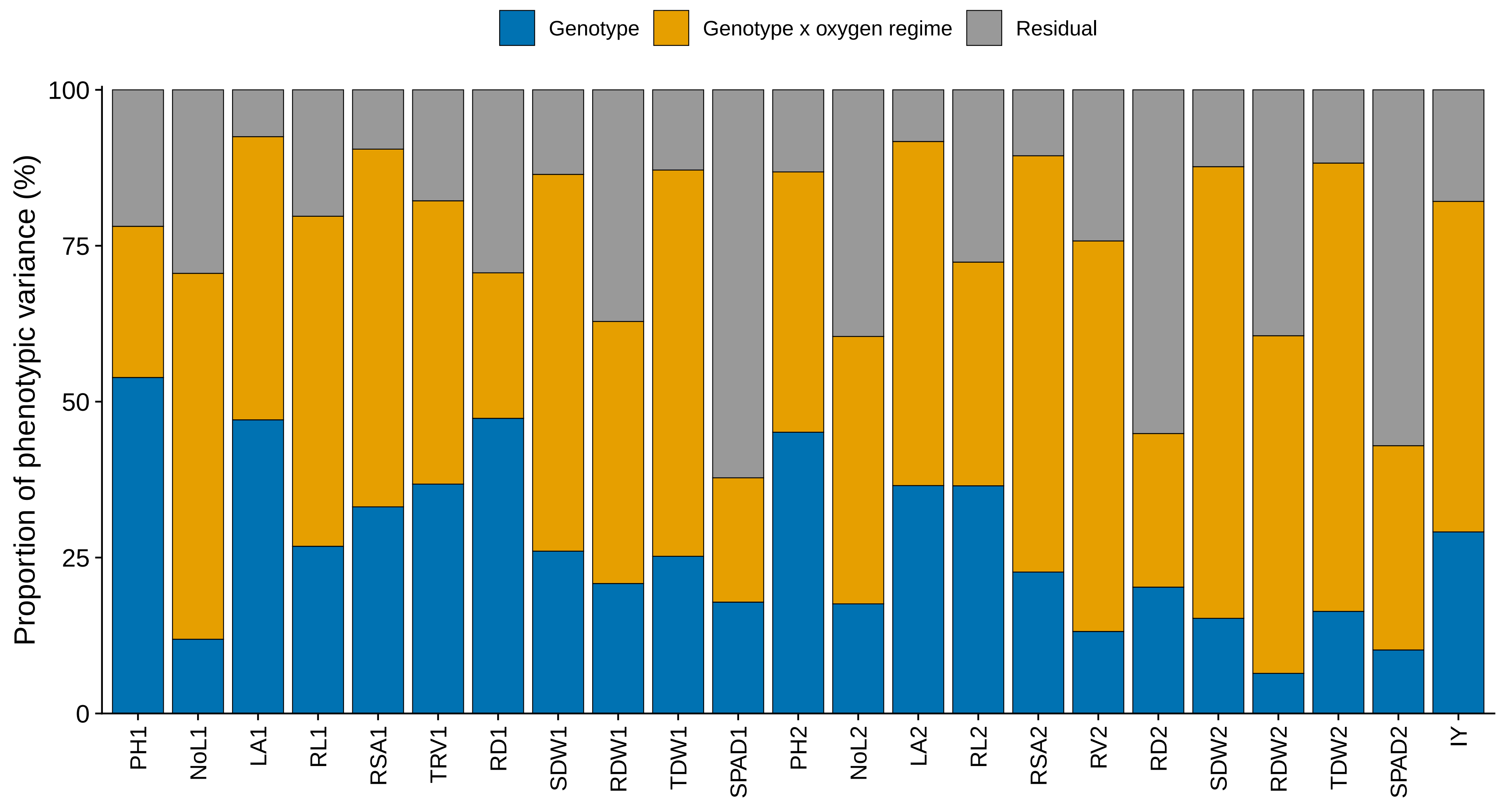
Genotype
Genotype x oxygen regime
Residual
Proportion of phenotypic variance (%)
0
25
50
75
100
PH1
NoL1
LA1
RL1
RSA1
TRV1
RD1
SDW1
RDW1
TDW1
SPAD1
PH2
NoL2
LA2
RL2
RSA2
RV2
RD2
SDW2
RDW2
TDW2
SPAD2
IY
