# Supplementary material for: Genotypic variation and root traits associated with hypoxia tolerance in quinoa (Chenopodium quinoa Willd.)
Source: PLoS One. 2026 Nov 3;21(11):e0361654. doi: 10.1371/journal.pone.0361654 (PMC13730327; doi:10.1371/journal.pone.0361654)
Supplement: Supplementary file 2 [file pone.0361654.s001.zip › S1_Analysis_Code/outputs/ML_leave_one_genotype_out/ML_LOGO_observed_vs_predicted.pdf]

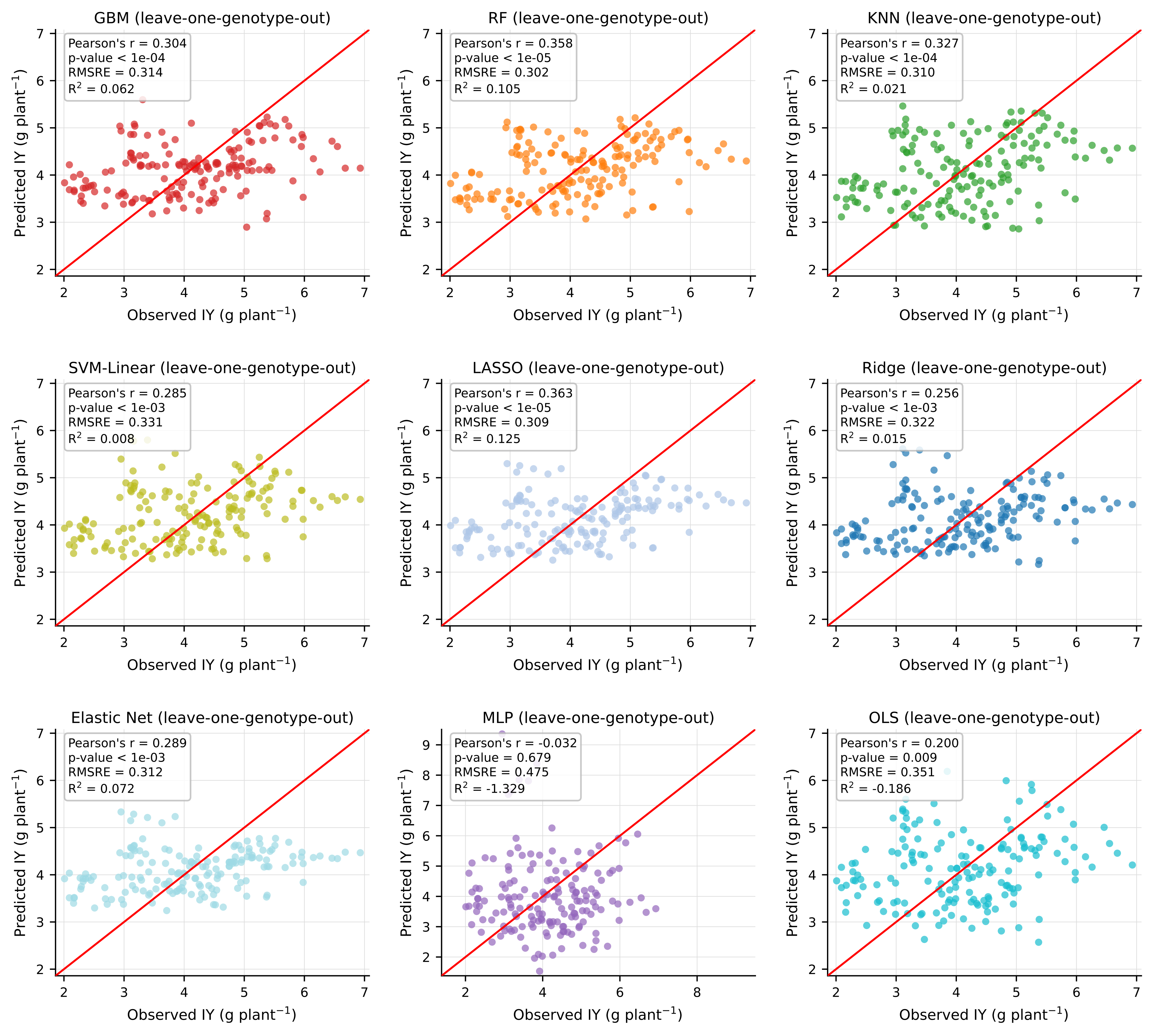
GBM (leave-one-genotype-out)
Pearson's r = 0.304
p-value < 1e-04
RMSRE = 0.314
R^2 = 0.062
RF (leave-one-genotype-out)
Pearson's r = 0.358
p-value < 1e-05
RMSRE = 0.302
R^2 = 0.105
KNN (leave-one-genotype-out)
Pearson's r = 0.327
p-value < 1e-04
RMSRE = 0.310
R^2 = 0.021
SVM-Linear (leave-one-genotype-out)
Pearson's r = 0.285
p-value < 1e-03
RMSRE = 0.331
R^2 = 0.008
LASSO (leave-one-genotype-out)
Pearson's r = 0.363
p-value < 1e-05
RMSRE = 0.309
R^2 = 0.125
Ridge (leave-one-genotype-out)
Pearson's r = 0.256
p-value < 1e-03
RMSRE = 0.322
R^2 = 0.015
Elastic Net (leave-one-genotype-out)
Pearson's r = 0.289
p-value < 1e-03
RMSRE = 0.312
R^2 = 0.072
MLP (leave-one-genotype-out)
Pearson's r = -0.032
p-value = 0.679
RMSRE = 0.475
R^2 = -1.329
OLS (leave-one-genotype-out)
Pearson's r = 0.200
p-value = 0.009
RMSRE = 0.351
R^2 = -0.186
Predicted IY (g plant^{-1})
Observed IY (g plant^{-1})
