# Supplementary material for: Genotypic variation and root traits associated with hypoxia tolerance in quinoa (Chenopodium quinoa Willd.)
Source: PLoS One. 2026 Nov 3;21(11):e0361654. doi: 10.1371/journal.pone.0361654 (PMC13730327; doi:10.1371/journal.pone.0361654)
Supplement: Supplementary file 2 [file pone.0361654.s001.zip › S1_Analysis_Code/outputs/ML_leave_one_genotype_out/ML_LOGO_RF_only.pdf]

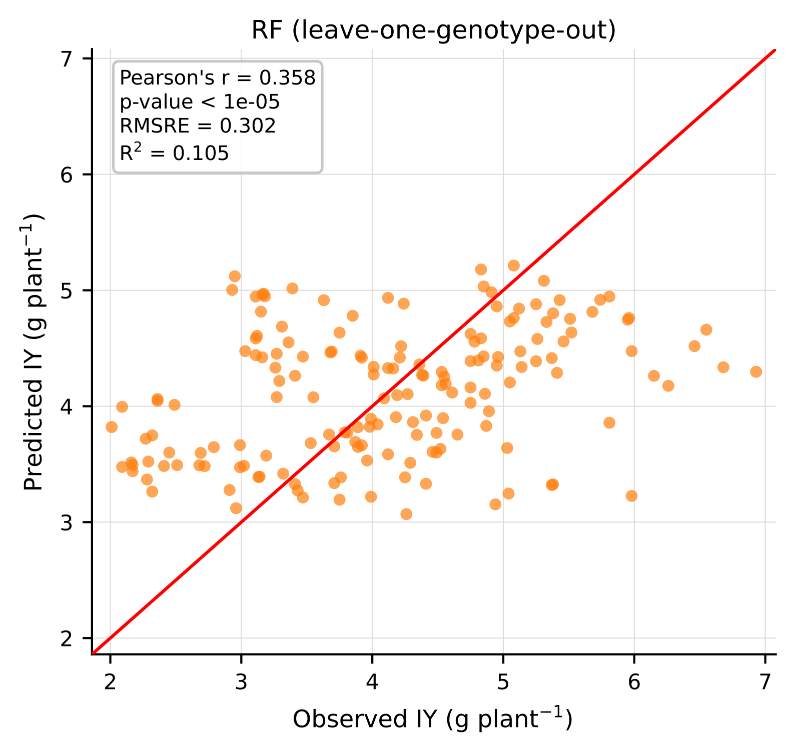

RF (leave-one-genotype-out)
Pearson's r = 0.358
p-value < 1e-05
RMSRE = 0.302
R^2 = 0.105
Predicted IY (g plant^{-1})
Observed IY (g plant^{-1})
2
3
4
5
6
7
