# Supplementary material for: Genotypic variation and root traits associated with hypoxia tolerance in quinoa (Chenopodium quinoa Willd.)
Source: PLoS One. 2026 Nov 3;21(11):e0361654. doi: 10.1371/journal.pone.0361654 (PMC13730327; doi:10.1371/journal.pone.0361654)
Supplement: Supplementary file 2 [file pone.0361654.s001.zip › S1_Analysis_Code/outputs/ML_random_split/ML_random_split.pdf]

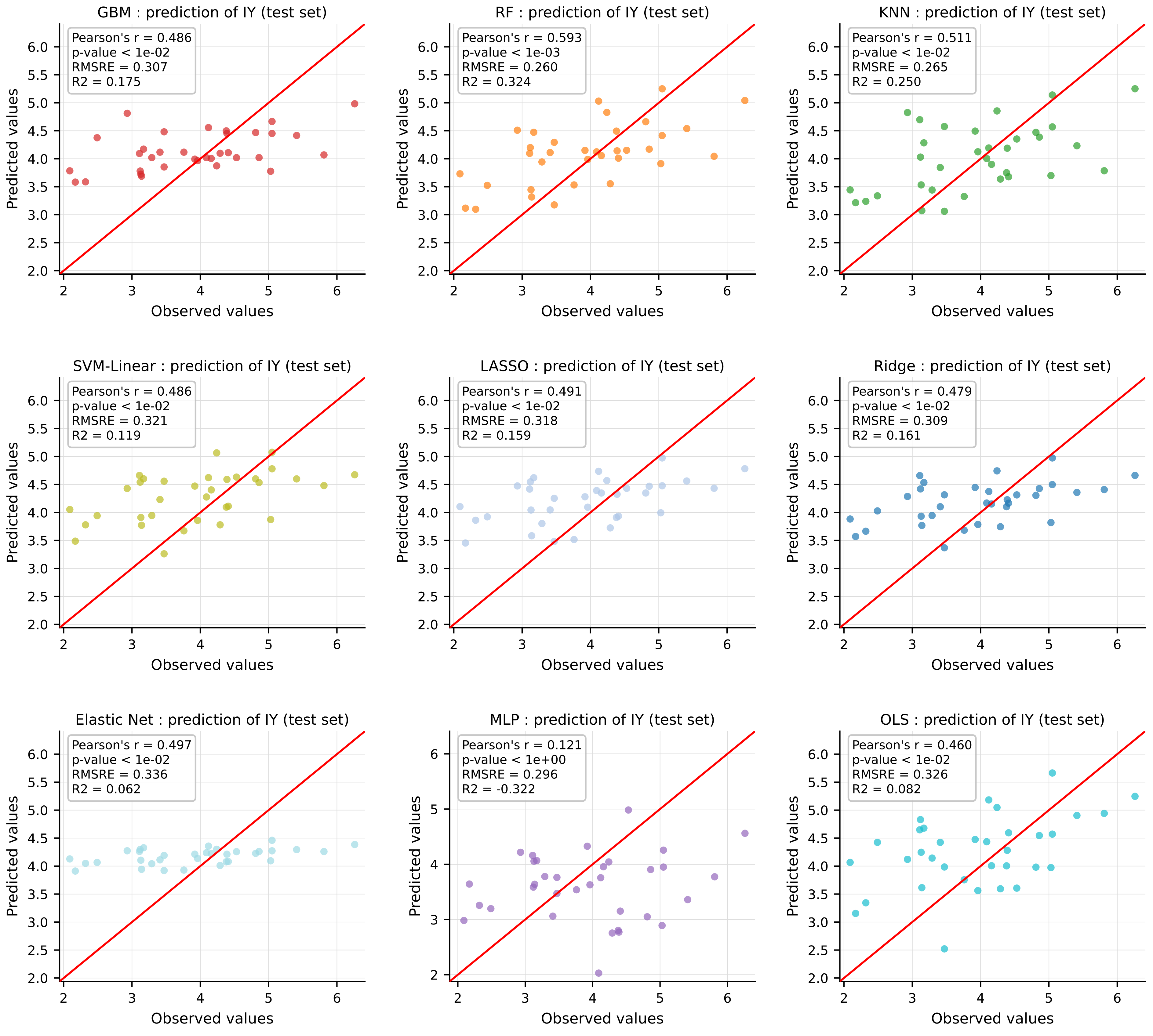
GBM : prediction of IY (test set)
Pearson's r = 0.486
p-value < 1e-02
RMSRE = 0.307
R2 = 0.175
RF : prediction of IY (test set)
Pearson's r = 0.593
p-value < 1e-03
RMSRE = 0.260
R2 = 0.324
KNN : prediction of IY (test set)
Pearson's r = 0.511
p-value < 1e-02
RMSRE = 0.265
R2 = 0.250
SVM-Linear : prediction of IY (test set)
Pearson's r = 0.486
p-value < 1e-02
RMSRE = 0.321
R2 = 0.119
LASSO : prediction of IY (test set)
Pearson's r = 0.491
p-value < 1e-02
RMSRE = 0.318
R2 = 0.159
Ridge : prediction of IY (test set)
Pearson's r = 0.479
p-value < 1e-02
RMSRE = 0.309
R2 = 0.161
Elastic Net : prediction of IY (test set)
Pearson's r = 0.497
p-value < 1e-02
RMSRE = 0.336
R2 = 0.062
MLP : prediction of IY (test set)
Pearson's r = 0.121
p-value < 1e+00
RMSRE = 0.296
R2 = -0.322
OLS : prediction of IY (test set)
Pearson's r = 0.460
p-value < 1e-02
RMSRE = 0.326
R2 = 0.082
Predicted values
Observed values
