# Supplementary material for: Genotypic variation and root traits associated with hypoxia tolerance in quinoa (Chenopodium quinoa Willd.)
Source: PLoS One. 2026 Nov 3;21(11):e0361654. doi: 10.1371/journal.pone.0361654 (PMC13730327; doi:10.1371/journal.pone.0361654)
Supplement: Supplementary file 2 [file pone.0361654.s001.zip › S1_Analysis_Code/outputs/PCA/PCA_biplot.pdf]

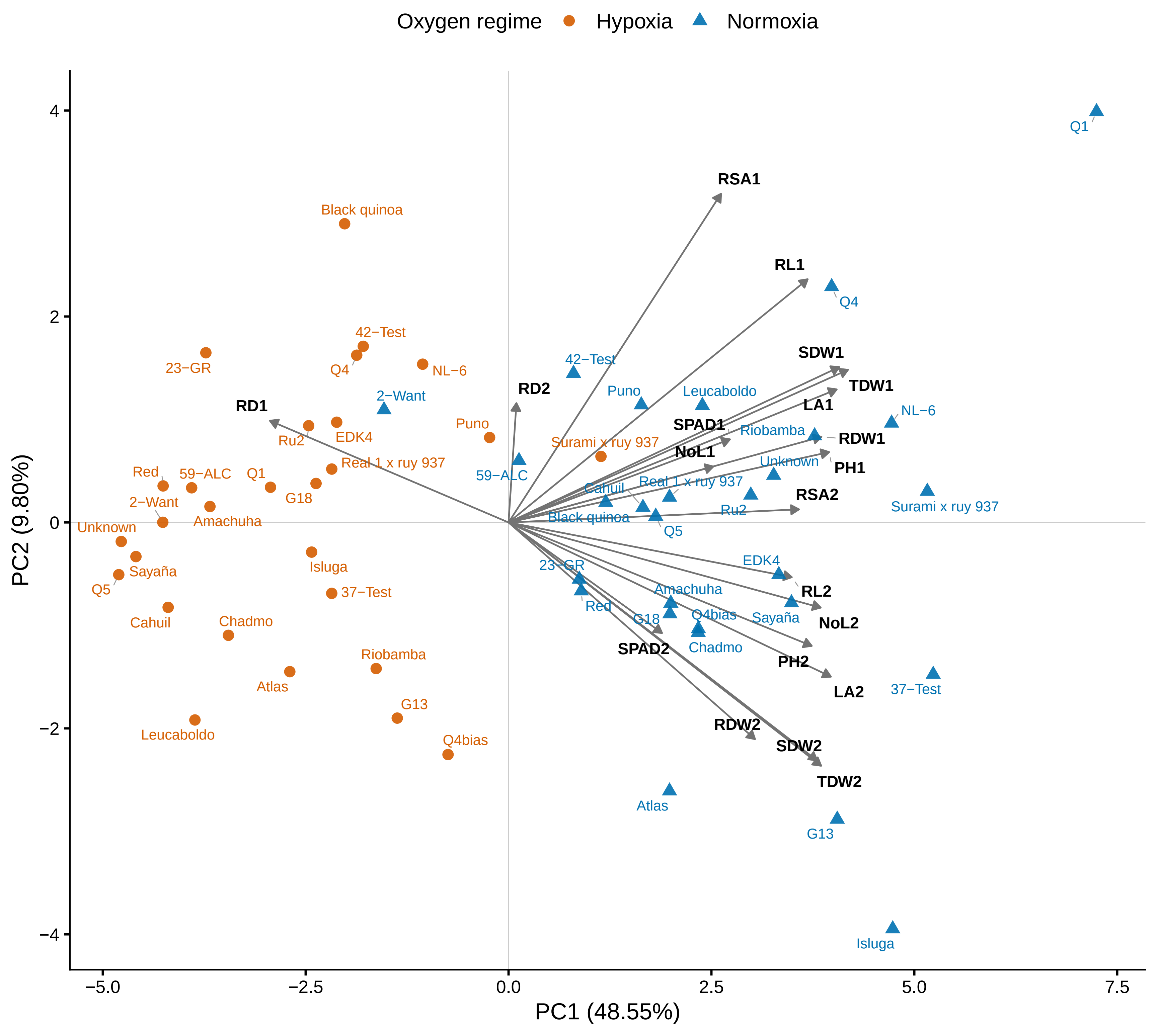

Oxygen regime
Hypoxia
Normoxia
PC1 (48.55%)
PC2 (9.80%)
−5.0
−2.5
0.0
2.5
5.0
7.5
4
2
0
−2
−4
RSA1
RL1
SDW1
TDW1
LA1
RD2
RD1
SPAD1
RDW1
NoL1
PH1
RSA2
RL2
NoL2
SPAD2
PH2
LA2
RDW2
SDW2
TDW2
Black quinoa
42−Test
23−GR
Q4
NL−6
2−Want
Ru2
EDK4
Puno
Surami x ruy 937
Red
59−ALC
Q1
Real 1 x ruy 937
G18
Amachuha
Unknown
Sayaña
Q5
Isluga
37−Test
Cahuil
Chadmo
Riobamba
Atlas
G13
Leucaboldo
Q4bias
