# Supplementary material for: Genotypic variation and root traits associated with hypoxia tolerance in quinoa (Chenopodium quinoa Willd.)
Source: PLoS One. 2026 Nov 3;21(11):e0361654. doi: 10.1371/journal.pone.0361654 (PMC13730327; doi:10.1371/journal.pone.0361654)
Supplement: Supplementary file 2 [file pone.0361654.s001.zip › S1_Analysis_Code/outputs/Schematic/experimental_schematic.pdf]

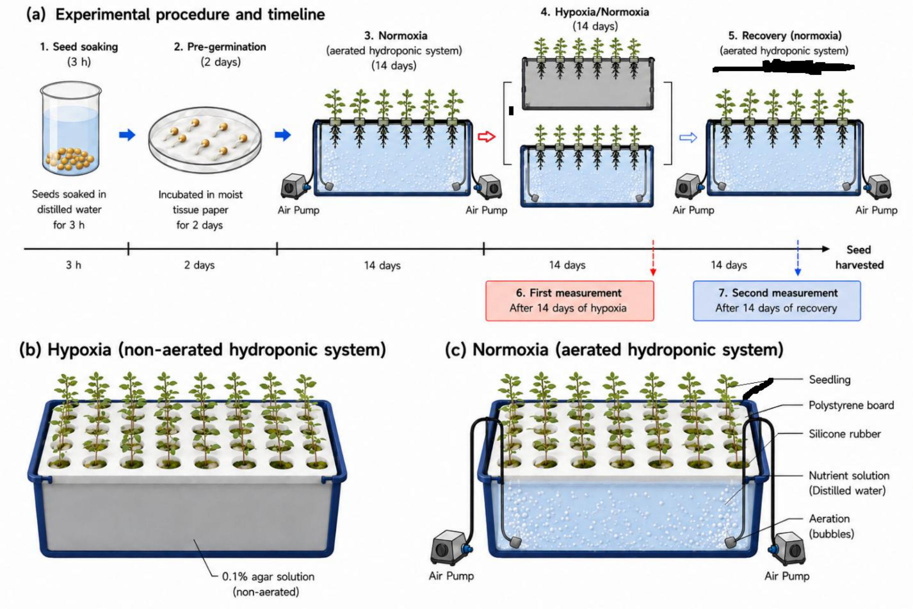

(a) Experimental procedure and timeline
1. Seed soaking
(3 h)
2. Pre-germination
(2 days)
3. Normoxia
(aerated hydroponic system)
(14 days)
4. Hypoxia/Normoxia
(14 days)
5. Recovery (normoxia)
(aerated hydroponic system)
[redacted]
Seeds soaked in distilled water for 3 h
Incubated in moist tissue paper for 2 days
Air Pump
Air Pump
Air Pump
Air Pump
3 h
2 days
14 days
14 days
14 days
Seed harvested
6. First measurement
After 14 days of hypoxia
7. Second measurement
After 14 days of recovery
(b) Hypoxia (non-aerated hydroponic system)
0.1% agar solution
(non-aerated)
(c) Normoxia (aerated hydroponic system)
Seedling
Polystyrene board
Silicone rubber
Nutrient solution
(Distilled water)
Aeration
(bubbles)
Air Pump
Air Pump
