# Supplementary material for: Genotypic variation and root traits associated with hypoxia tolerance in quinoa (Chenopodium quinoa Willd.)
Source: PLoS One. 2026 Nov 3;21(11):e0361654. doi: 10.1371/journal.pone.0361654 (PMC13730327; doi:10.1371/journal.pone.0361654)
Supplement: Supplementary file 2 [file pone.0361654.s001.zip › S1_Analysis_Code/outputs/withdrawn_GGE/GGE_mean_vs_stability.pdf]

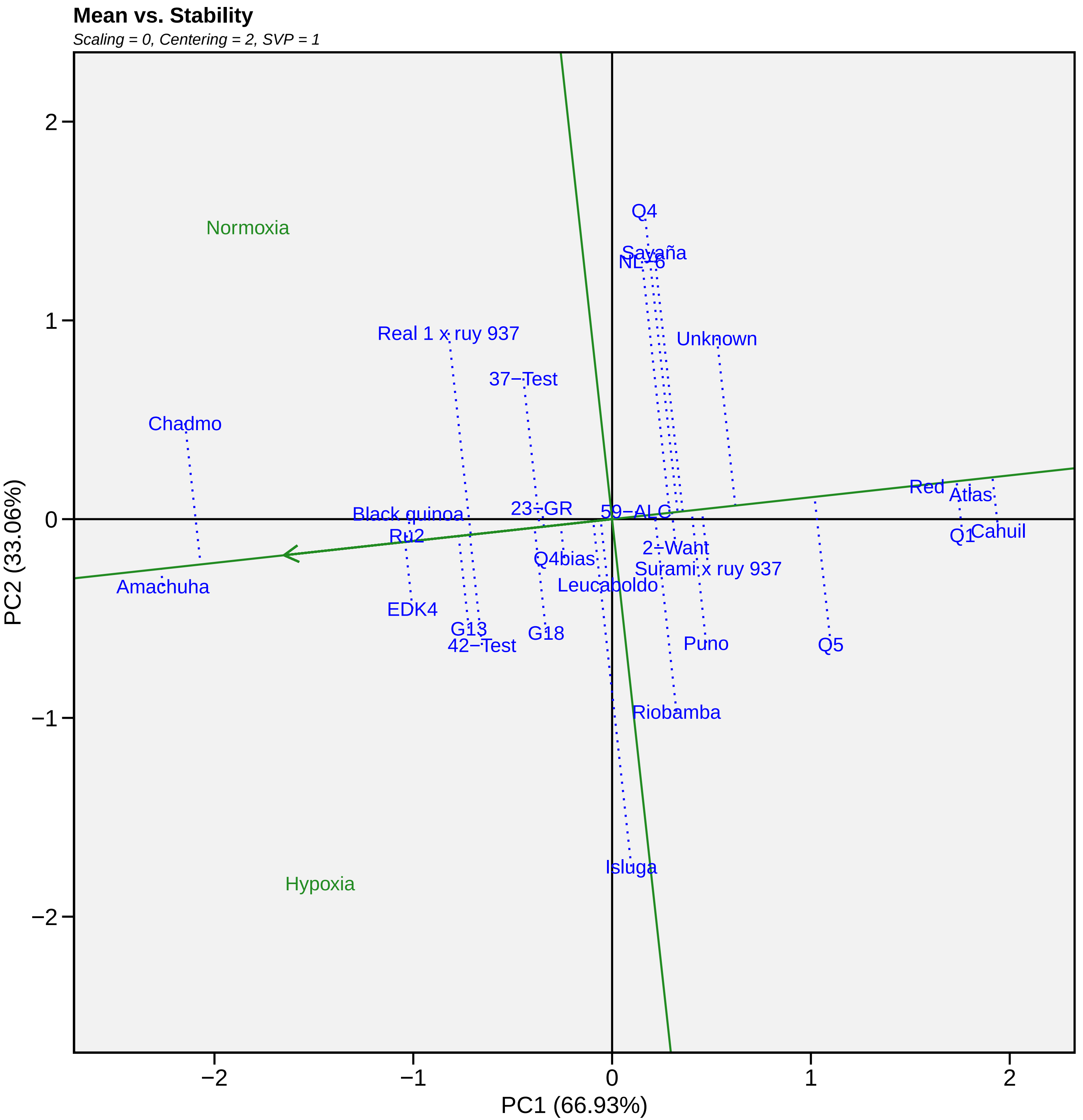

Mean vs. Stability
Scaling = 0, Centering = 2, SVP = 1
Normoxia
Hypoxia
Q4
Sayaña
NL−6
Real 1 x ruy 937
Unknown
37−Test
Chadmo
Red
Atlas
Black quinoa
23−GR
59−ALC
Ru2
Q1
Cahuil
2−Want
Q4bias
Surami x ruy 937
Amachuha
Leucaboldo
EDK4
G13
G18
42−Test
Puno
Q5
Riobamba
Isluga
PC1 (66.93%)
PC2 (33.06%)
