# Supplementary material for: Genotypic variation and root traits associated with hypoxia tolerance in quinoa (Chenopodium quinoa Willd.)
Source: PLoS One. 2026 Nov 3;21(11):e0361654. doi: 10.1371/journal.pone.0361654 (PMC13730327; doi:10.1371/journal.pone.0361654)

Which−won−where view of the GGE biplot

Scaling = 0, Centering = 2, SVP = 1

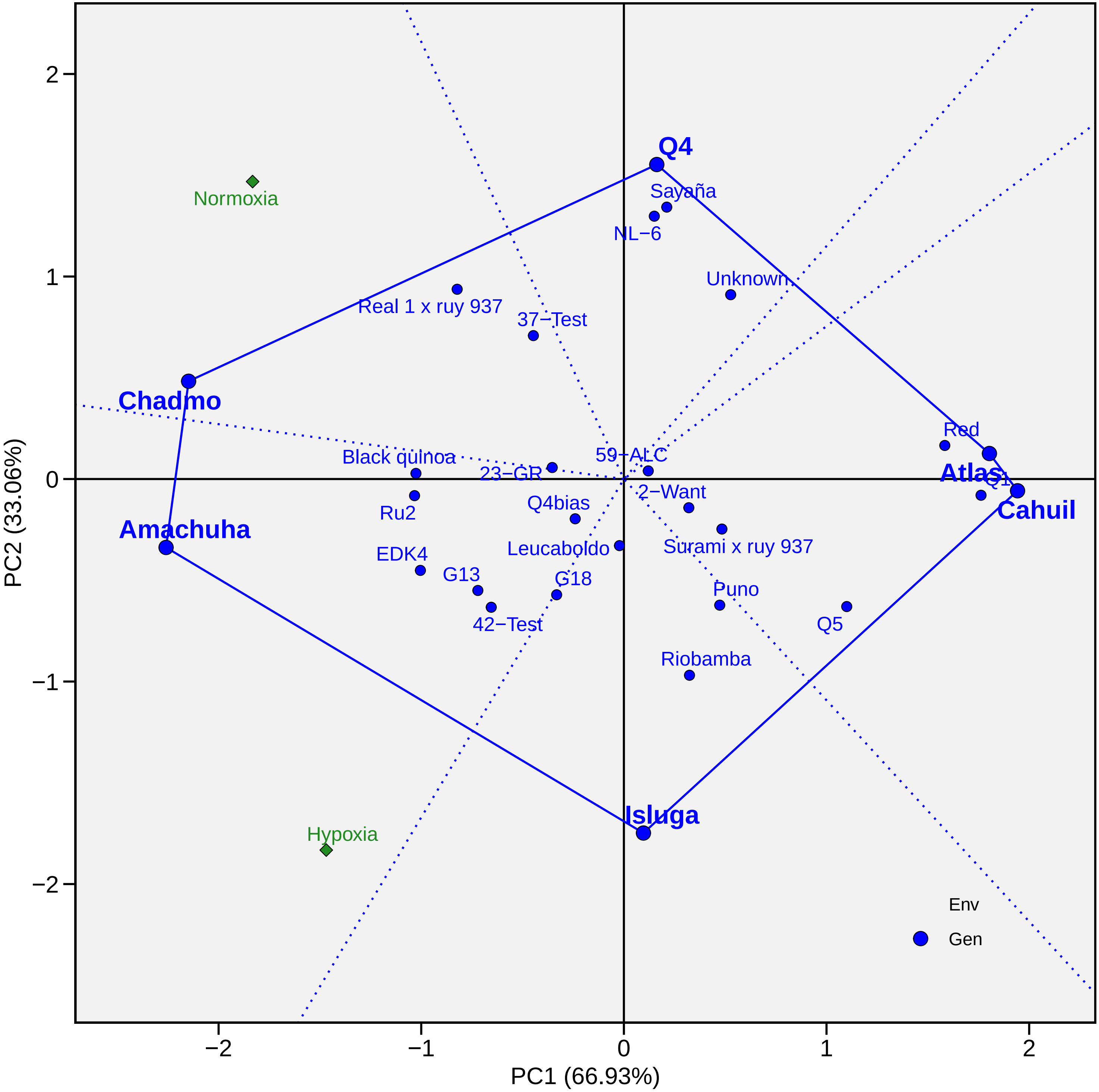

Supplement: Supplementary file 2 [file pone.0361654.s001.zip › S1_Analysis_Code/outputs/withdrawn_GGE/GGE_which_won_where.pdf]
