# Supplementary material for: Genotypic variation and root traits associated with hypoxia tolerance in quinoa (Chenopodium quinoa Willd.)
Source: PLoS One. 2026 Nov 3;21(11):e0361654. doi: 10.1371/journal.pone.0361654 (PMC13730327; doi:10.1371/journal.pone.0361654)
Supplement: Supplementary file 3 [file pone.0361654.s002.docx]

**S1 Table.** Root traits of 28 quinoa genotypes under normoxia and hypoxia and their hypoxia tolerance index (HTI). RL1, RSA1, and RD1: root length (cm), root surface area (cm²), and root diameter (mm) at the end of the 14-day hypoxia treatment (30 days after sowing, DAS); RL2, RSA2, and RD2: the same traits after 14 days of recovery (44 DAS). Values are means of three replicates; HTI = hypoxia mean / normoxia mean of each genotype.

| **Genotype** | **RL1 (cm)** | | | **RSA1 (cm²)** | | | **RD1 (mm)** | | | **RL2 (cm)** | | | **RSA2 (cm²)** | | | **RD2 (mm)** | | |
| --- | --- | --- | --- | --- | --- | --- | --- | --- | --- | --- | --- | --- | --- | --- | --- | --- | --- | --- |
|  | **Normoxia** | **Hypoxia** | **HTI** | **Normoxia** | **Hypoxia** | **HTI** | **Normoxia** | **Hypoxia** | **HTI** | **Normoxia** | **Hypoxia** | **HTI** | **Normoxia** | **Hypoxia** | **HTI** | **Normoxia** | **Hypoxia** | **HTI** |
| 2-Want | 701.68 | 421.92 | 0.60 | 35.09 | 30.09 | 0.86 | 0.160 | 0.227 | 1.42 | 926.76 | 776.06 | 0.84 | 44.71 | 38.04 | 0.85 | 0.154 | 0.161 | 1.05 |
| 23-GR | 687.39 | 509.14 | 0.74 | 48.30 | 41.56 | 0.86 | 0.223 | 0.263 | 1.18 | 1122.05 | 736.69 | 0.66 | 49.12 | 40.12 | 0.82 | 0.148 | 0.175 | 1.18 |
| 37-Test | 718.95 | 330.38 | 0.46 | 34.55 | 16.60 | 0.48 | 0.153 | 0.160 | 1.04 | 930.55 | 765.07 | 0.82 | 50.10 | 37.85 | 0.76 | 0.172 | 0.158 | 0.91 |
| 42-Test | 897.09 | 609.79 | 0.68 | 58.54 | 45.46 | 0.78 | 0.210 | 0.237 | 1.13 | 922.21 | 590.05 | 0.64 | 34.44 | 32.16 | 0.93 | 0.119 | 0.174 | 1.46 |
| 59-ALC | 831.28 | 510.95 | 0.61 | 36.35 | 35.24 | 0.97 | 0.147 | 0.230 | 1.57 | 968.76 | 771.03 | 0.80 | 40.05 | 32.40 | 0.81 | 0.132 | 0.135 | 1.03 |
| Amachuha | 466.68 | 172.25 | 0.37 | 23.51 | 13.73 | 0.58 | 0.163 | 0.253 | 1.55 | 1283.11 | 943.27 | 0.74 | 43.37 | 37.06 | 0.85 | 0.108 | 0.129 | 1.20 |
| Atlas | 540.02 | 236.03 | 0.44 | 27.32 | 14.90 | 0.55 | 0.160 | 0.200 | 1.25 | 1793.48 | 1118.04 | 0.62 | 59.09 | 36.53 | 0.62 | 0.105 | 0.104 | 0.99 |
| Black quinoa | 364.23 | 661.85 | 1.82 | 22.64 | 59.70 | 2.64 | 0.200 | 0.293 | 1.47 | 1306.87 | 1061.16 | 0.81 | 65.38 | 46.86 | 0.72 | 0.159 | 0.142 | 0.89 |
| Cahuil | 397.92 | 239.86 | 0.60 | 34.54 | 17.34 | 0.50 | 0.277 | 0.240 | 0.87 | 1397.35 | 836.89 | 0.60 | 56.87 | 34.09 | 0.60 | 0.130 | 0.131 | 1.01 |
| Chadmo | 739.68 | 439.21 | 0.59 | 39.63 | 27.71 | 0.70 | 0.170 | 0.203 | 1.20 | 882.44 | 1019.80 | 1.16 | 42.46 | 35.85 | 0.84 | 0.153 | 0.112 | 0.73 |
| EDK4 | 1074.12 | 801.41 | 0.75 | 52.52 | 46.95 | 0.89 | 0.160 | 0.193 | 1.21 | 1590.55 | 856.36 | 0.54 | 78.20 | 33.53 | 0.43 | 0.157 | 0.125 | 0.80 |
| G13 | 732.58 | 459.51 | 0.63 | 28.35 | 24.31 | 0.86 | 0.123 | 0.170 | 1.38 | 1139.14 | 804.19 | 0.71 | 45.29 | 34.89 | 0.77 | 0.126 | 0.142 | 1.13 |
| G18 | 517.81 | 487.58 | 0.94 | 26.71 | 25.31 | 0.95 | 0.167 | 0.167 | 1.00 | 1641.52 | 713.12 | 0.43 | 75.08 | 33.68 | 0.45 | 0.149 | 0.152 | 1.02 |
| Isluga | 443.29 | 332.95 | 0.75 | 22.08 | 18.29 | 0.83 | 0.160 | 0.177 | 1.10 | 1877.97 | 1245.67 | 0.66 | 77.32 | 48.35 | 0.63 | 0.131 | 0.127 | 0.97 |
| Leucaboldo | 986.44 | 253.58 | 0.26 | 52.41 | 19.87 | 0.38 | 0.173 | 0.257 | 1.48 | 1214.52 | 768.68 | 0.63 | 64.30 | 34.01 | 0.53 | 0.168 | 0.141 | 0.84 |
| NL-6 | 962.57 | 666.21 | 0.69 | 54.74 | 37.46 | 0.68 | 0.187 | 0.183 | 0.98 | 1745.79 | 1014.65 | 0.58 | 67.54 | 45.05 | 0.67 | 0.123 | 0.142 | 1.15 |
| Puno | 1001.60 | 750.75 | 0.75 | 61.65 | 51.80 | 0.84 | 0.210 | 0.223 | 1.06 | 1163.74 | 1014.85 | 0.87 | 53.99 | 48.89 | 0.91 | 0.148 | 0.154 | 1.05 |
| Q1 | 1591.66 | 317.27 | 0.20 | 67.72 | 24.34 | 0.36 | 0.137 | 0.247 | 1.80 | 1484.59 | 751.43 | 0.51 | 77.69 | 26.23 | 0.34 | 0.167 | 0.112 | 0.67 |
| Q4 | 1016.56 | 504.31 | 0.50 | 55.73 | 32.77 | 0.59 | 0.173 | 0.210 | 1.21 | 1570.18 | 976.36 | 0.62 | 69.71 | 35.54 | 0.51 | 0.141 | 0.128 | 0.91 |
| Q4bias | 550.88 | 340.12 | 0.62 | 27.74 | 16.61 | 0.60 | 0.163 | 0.153 | 0.94 | 1075.68 | 1196.46 | 1.11 | 46.92 | 51.20 | 1.09 | 0.145 | 0.140 | 0.96 |
| Q5 | 705.20 | 249.23 | 0.35 | 53.78 | 25.78 | 0.48 | 0.243 | 0.337 | 1.38 | 1048.57 | 676.44 | 0.65 | 43.79 | 32.00 | 0.73 | 0.134 | 0.152 | 1.13 |
| Real 1 x ruy 937 | 852.53 | 443.96 | 0.52 | 32.93 | 25.04 | 0.76 | 0.123 | 0.183 | 1.49 | 1460.85 | 634.77 | 0.43 | 62.51 | 26.29 | 0.42 | 0.137 | 0.134 | 0.98 |
| Red | 636.20 | 281.18 | 0.44 | 25.56 | 19.81 | 0.78 | 0.127 | 0.223 | 1.76 | 932.66 | 859.26 | 0.92 | 52.54 | 49.81 | 0.95 | 0.180 | 0.185 | 1.03 |
| Riobamba | 976.05 | 334.62 | 0.34 | 54.39 | 21.84 | 0.40 | 0.177 | 0.210 | 1.19 | 1867.86 | 1269.31 | 0.68 | 79.36 | 55.08 | 0.69 | 0.137 | 0.144 | 1.06 |
| Ru2 | 835.58 | 614.31 | 0.74 | 45.49 | 35.80 | 0.79 | 0.173 | 0.220 | 1.27 | 1001.95 | 632.67 | 0.63 | 47.84 | 24.78 | 0.52 | 0.153 | 0.125 | 0.82 |
| Sayaña | 990.28 | 400.71 | 0.40 | 36.56 | 19.28 | 0.53 | 0.120 | 0.153 | 1.28 | 914.21 | 480.92 | 0.53 | 34.01 | 20.07 | 0.59 | 0.119 | 0.135 | 1.13 |
| Surami x ruy 937 | 1083.81 | 1019.53 | 0.94 | 43.52 | 45.95 | 1.06 | 0.130 | 0.143 | 1.10 | 1255.74 | 1146.65 | 0.91 | 52.92 | 46.56 | 0.88 | 0.134 | 0.131 | 0.97 |
| Unknown | 828.80 | 218.16 | 0.26 | 49.49 | 20.62 | 0.42 | 0.193 | 0.300 | 1.55 | 1517.87 | 756.79 | 0.50 | 52.20 | 29.22 | 0.56 | 0.110 | 0.123 | 1.12 |
