# Supplementary material for: Genotypic variation and root traits associated with hypoxia tolerance in quinoa (Chenopodium quinoa Willd.)
Source: PLoS One. 2026 Nov 3;21(11):e0361654. doi: 10.1371/journal.pone.0361654 (PMC13730327; doi:10.1371/journal.pone.0361654)
Supplement: Supplementary file 4 [file pone.0361654.s003.docx]

**S2 Table.** Plant height (PH, cm) and number of leaves on the main stem (NoL) of 28 quinoa genotypes under normoxia and hypoxia and their hypoxia tolerance index (HTI). PH1 and NoL1: end of the 14-day hypoxia treatment (30 DAS); PH2 and NoL2: after 14 days of recovery (44 DAS). Values are means of three replicates; HTI = hypoxia mean / normoxia mean of each genotype.

| **Genotype** | **PH1 (cm)** | | | **PH2 (cm)** | | | **NoL1** | | | **NoL2** | | |
| --- | --- | --- | --- | --- | --- | --- | --- | --- | --- | --- | --- | --- |
|  | **Normoxia** | **Hypoxia** | **HTI** | **Normoxia** | **Hypoxia** | **HTI** | **Normoxia** | **Hypoxia** | **HTI** | **Normoxia** | **Hypoxia** | **HTI** |
| 2-Want | 17.00 | 12.67 | 0.75 | 31.67 | 26.00 | 0.82 | 14.00 | 10.67 | 0.76 | 18.67 | 18.00 | 0.96 |
| 23-GR | 28.33 | 19.00 | 0.67 | 45.33 | 18.33 | 0.40 | 14.33 | 18.33 | 1.28 | 21.67 | 15.00 | 0.69 |
| 37-Test | 33.00 | 21.00 | 0.64 | 62.33 | 48.67 | 0.78 | 20.67 | 15.00 | 0.73 | 28.00 | 19.67 | 0.70 |
| 42-Test | 21.33 | 16.33 | 0.77 | 47.67 | 31.67 | 0.66 | 17.33 | 15.00 | 0.87 | 24.67 | 21.00 | 0.85 |
| 59-ALC | 22.00 | 16.33 | 0.74 | 40.00 | 30.33 | 0.76 | 15.33 | 13.33 | 0.87 | 22.00 | 21.33 | 0.97 |
| Amachuha | 23.00 | 17.00 | 0.74 | 49.33 | 28.67 | 0.58 | 14.00 | 16.33 | 1.17 | 23.33 | 19.67 | 0.84 |
| Atlas | 23.67 | 16.00 | 0.68 | 46.67 | 32.67 | 0.70 | 17.00 | 13.67 | 0.80 | 25.67 | 23.33 | 0.91 |
| Black quinoa | 31.00 | 22.33 | 0.72 | 34.00 | 23.00 | 0.68 | 16.00 | 14.00 | 0.88 | 22.00 | 17.67 | 0.80 |
| Cahuil | 19.00 | 11.33 | 0.60 | 40.67 | 32.00 | 0.79 | 14.00 | 12.00 | 0.86 | 21.00 | 19.00 | 0.90 |
| Chadmo | 21.00 | 14.67 | 0.70 | 39.33 | 26.67 | 0.68 | 15.00 | 13.00 | 0.87 | 23.00 | 20.33 | 0.88 |
| EDK4 | 21.00 | 12.67 | 0.60 | 52.00 | 40.00 | 0.77 | 14.33 | 15.33 | 1.07 | 28.67 | 18.00 | 0.63 |
| G13 | 32.33 | 20.33 | 0.63 | 46.33 | 41.33 | 0.89 | 18.33 | 12.67 | 0.69 | 32.00 | 21.33 | 0.67 |
| G18 | 25.00 | 16.33 | 0.65 | 42.00 | 37.33 | 0.89 | 19.67 | 17.67 | 0.90 | 22.67 | 18.67 | 0.82 |
| Isluga | 21.33 | 15.00 | 0.70 | 52.00 | 32.33 | 0.62 | 14.33 | 14.67 | 1.02 | 26.67 | 20.33 | 0.76 |
| Leucaboldo | 20.33 | 8.00 | 0.39 | 45.67 | 38.67 | 0.85 | 14.67 | 11.33 | 0.77 | 24.67 | 20.67 | 0.84 |
| NL-6 | 25.33 | 17.00 | 0.67 | 39.33 | 41.33 | 1.05 | 16.33 | 16.00 | 0.98 | 27.00 | 22.67 | 0.84 |
| Puno | 22.33 | 18.67 | 0.84 | 57.67 | 39.67 | 0.69 | 17.00 | 16.00 | 0.94 | 24.67 | 24.33 | 0.99 |
| Q1 | 36.33 | 19.67 | 0.54 | 49.00 | 41.67 | 0.85 | 17.33 | 14.33 | 0.83 | 26.67 | 22.33 | 0.84 |
| Q4 | 32.00 | 24.00 | 0.75 | 39.33 | 31.33 | 0.80 | 15.00 | 18.33 | 1.22 | 27.67 | 21.33 | 0.77 |
| Q4bias | 24.00 | 20.33 | 0.85 | 58.67 | 44.67 | 0.76 | 16.67 | 15.00 | 0.90 | 26.00 | 18.67 | 0.72 |
| Q5 | 28.67 | 15.67 | 0.55 | 48.00 | 27.67 | 0.58 | 17.67 | 14.67 | 0.83 | 21.00 | 20.33 | 0.97 |
| Real 1 x ruy 937 | 25.00 | 23.00 | 0.92 | 39.33 | 31.67 | 0.81 | 14.67 | 13.33 | 0.91 | 26.00 | 20.33 | 0.78 |
| Red | 20.33 | 20.67 | 1.02 | 46.33 | 16.67 | 0.36 | 15.33 | 12.67 | 0.83 | 20.67 | 16.33 | 0.79 |
| Riobamba | 23.33 | 17.67 | 0.76 | 43.00 | 42.00 | 0.98 | 17.67 | 15.67 | 0.89 | 22.00 | 24.67 | 1.12 |
| Ru2 | 27.67 | 18.67 | 0.67 | 46.33 | 34.00 | 0.73 | 14.67 | 15.67 | 1.07 | 23.33 | 19.67 | 0.84 |
| Sayaña | 28.33 | 16.67 | 0.59 | 58.67 | 38.00 | 0.65 | 18.67 | 15.33 | 0.82 | 28.67 | 19.67 | 0.69 |
| Surami x ruy 937 | 31.00 | 22.67 | 0.73 | 59.00 | 49.00 | 0.83 | 23.00 | 13.67 | 0.59 | 35.67 | 20.67 | 0.58 |
| Unknown | 30.00 | 17.00 | 0.57 | 57.33 | 25.33 | 0.44 | 18.33 | 16.33 | 0.89 | 28.00 | 20.00 | 0.71 |
