# Supplementary material for: Genotypic variation and root traits associated with hypoxia tolerance in quinoa (Chenopodium quinoa Willd.)
Source: PLoS One. 2026 Nov 3;21(11):e0361654. doi: 10.1371/journal.pone.0361654 (PMC13730327; doi:10.1371/journal.pone.0361654)
Supplement: Supplementary file 5 [file pone.0361654.s004.docx]

**S3 Table.** Leaf area (LA, cm²) and SPAD value of 28 quinoa genotypes under normoxia and hypoxia and their hypoxia tolerance index (HTI). LA1 and SPAD1: end of the 14-day hypoxia treatment (30 DAS); LA2 and SPAD2: after 14 days of recovery (44 DAS). Values are means of three replicates; HTI = hypoxia mean / normoxia mean of each genotype.

| **Genotype** | **LA1 (cm²)** | | | **LA2 (cm²)** | | | **SPAD1** | | | **SPAD2** | | |
| --- | --- | --- | --- | --- | --- | --- | --- | --- | --- | --- | --- | --- |
|  | **Normoxia** | **Hypoxia** | **HTI** | **Normoxia** | **Hypoxia** | **HTI** | **Normoxia** | **Hypoxia** | **HTI** | **Normoxia** | **Hypoxia** | **HTI** |
| 2-Want | 88.57 | 35.00 | 0.40 | 76.93 | 96.74 | 1.26 | 46.43 | 40.60 | 0.87 | 47.43 | 50.50 | 1.06 |
| 23-GR | 79.12 | 67.25 | 0.85 | 199.74 | 47.82 | 0.24 | 49.17 | 45.80 | 0.93 | 51.30 | 44.47 | 0.87 |
| 37-Test | 125.71 | 42.07 | 0.33 | 233.73 | 152.09 | 0.65 | 47.97 | 47.43 | 0.99 | 54.07 | 48.63 | 0.90 |
| 42-Test | 118.27 | 90.74 | 0.77 | 182.46 | 135.57 | 0.74 | 44.87 | 44.33 | 0.99 | 47.50 | 44.10 | 0.93 |
| 59-ALC | 98.62 | 33.96 | 0.34 | 181.26 | 94.91 | 0.52 | 46.33 | 44.20 | 0.95 | 45.83 | 44.67 | 0.97 |
| Amachuha | 132.87 | 59.55 | 0.45 | 184.06 | 90.19 | 0.49 | 48.83 | 42.10 | 0.86 | 50.47 | 42.93 | 0.85 |
| Atlas | 82.00 | 56.86 | 0.69 | 208.60 | 139.50 | 0.67 | 48.70 | 45.50 | 0.93 | 44.03 | 48.37 | 1.10 |
| Black quinoa | 125.90 | 93.75 | 0.74 | 147.31 | 93.02 | 0.63 | 49.53 | 46.27 | 0.93 | 53.30 | 48.47 | 0.91 |
| Cahuil | 107.85 | 51.53 | 0.48 | 173.61 | 118.90 | 0.68 | 48.97 | 41.03 | 0.84 | 51.63 | 47.63 | 0.92 |
| Chadmo | 104.85 | 26.70 | 0.25 | 257.09 | 150.50 | 0.59 | 47.63 | 45.17 | 0.95 | 45.67 | 42.23 | 0.92 |
| EDK4 | 95.23 | 55.87 | 0.59 | 182.55 | 127.55 | 0.70 | 51.30 | 45.47 | 0.89 | 49.70 | 46.40 | 0.93 |
| G13 | 110.65 | 53.48 | 0.48 | 223.39 | 171.18 | 0.77 | 36.40 | 35.80 | 0.98 | 48.97 | 49.57 | 1.01 |
| G18 | 78.78 | 68.26 | 0.87 | 166.06 | 114.49 | 0.69 | 50.17 | 42.07 | 0.84 | 48.80 | 49.07 | 1.01 |
| Isluga | 110.08 | 75.66 | 0.69 | 185.30 | 116.33 | 0.63 | 51.30 | 45.17 | 0.88 | 50.33 | 40.23 | 0.80 |
| Leucaboldo | 95.18 | 28.77 | 0.30 | 173.55 | 127.82 | 0.74 | 51.73 | 44.10 | 0.85 | 49.50 | 49.93 | 1.01 |
| NL-6 | 155.55 | 76.03 | 0.49 | 194.95 | 142.60 | 0.73 | 51.93 | 45.80 | 0.88 | 51.83 | 42.07 | 0.81 |
| Puno | 84.44 | 54.43 | 0.64 | 199.21 | 170.16 | 0.85 | 48.30 | 49.37 | 1.02 | 42.13 | 46.80 | 1.11 |
| Q1 | 148.90 | 74.82 | 0.50 | 181.28 | 102.91 | 0.57 | 46.10 | 45.80 | 0.99 | 48.60 | 42.17 | 0.87 |
| Q4 | 145.70 | 81.67 | 0.56 | 157.81 | 94.16 | 0.60 | 49.67 | 45.23 | 0.91 | 46.80 | 41.97 | 0.90 |
| Q4bias | 79.19 | 52.23 | 0.66 | 181.20 | 164.84 | 0.91 | 53.37 | 41.87 | 0.78 | 46.00 | 50.60 | 1.10 |
| Q5 | 92.60 | 36.83 | 0.40 | 223.93 | 76.33 | 0.34 | 49.20 | 39.23 | 0.80 | 51.60 | 54.47 | 1.06 |
| Real 1 x ruy 937 | 126.00 | 79.40 | 0.63 | 166.17 | 77.47 | 0.47 | 47.90 | 46.27 | 0.97 | 48.60 | 48.13 | 0.99 |
| Red | 93.33 | 33.18 | 0.36 | 170.09 | 65.06 | 0.38 | 45.67 | 47.80 | 1.05 | 52.93 | 49.73 | 0.94 |
| Riobamba | 85.52 | 33.02 | 0.39 | 183.59 | 155.76 | 0.85 | 48.70 | 44.37 | 0.91 | 50.53 | 52.10 | 1.03 |
| Ru2 | 131.46 | 57.83 | 0.44 | 145.64 | 87.89 | 0.60 | 46.13 | 44.10 | 0.96 | 49.83 | 49.57 | 0.99 |
| Sayaña | 91.97 | 39.28 | 0.43 | 191.97 | 73.78 | 0.38 | 47.40 | 38.63 | 0.82 | 52.13 | 44.57 | 0.85 |
| Surami x ruy 937 | 97.86 | 81.60 | 0.83 | 199.32 | 167.23 | 0.84 | 46.47 | 49.40 | 1.06 | 51.70 | 47.30 | 0.91 |
| Unknown | 91.39 | 36.16 | 0.40 | 173.00 | 77.62 | 0.45 | 48.60 | 42.53 | 0.88 | 47.27 | 37.80 | 0.80 |
