# Supplementary material for: Genotypic variation and root traits associated with hypoxia tolerance in quinoa (Chenopodium quinoa Willd.)
Source: PLoS One. 2026 Nov 3;21(11):e0361654. doi: 10.1371/journal.pone.0361654 (PMC13730327; doi:10.1371/journal.pone.0361654)
Supplement: Supplementary file 6 [file pone.0361654.s005.docx]

**S4 Table.** Shoot dry weight (SDW, g plant⁻¹) and root dry weight (RDW, g plant⁻¹) of 28 quinoa genotypes under normoxia and hypoxia and their hypoxia tolerance index (HTI). SDW1 and RDW1: end of the 14-day hypoxia treatment (30 DAS); SDW2 and RDW2: after 14 days of recovery (44 DAS). Values are means of three replicates; HTI = hypoxia mean / normoxia mean of each genotype.

| **Genotype** | **SDW1 (g plant⁻¹)** | | | **RDW1 (g plant⁻¹)** | | | **SDW2 (g plant⁻¹)** | | | **RDW2 (g plant⁻¹)** | | |
| --- | --- | --- | --- | --- | --- | --- | --- | --- | --- | --- | --- | --- |
|  | **Normoxia** | **Hypoxia** | **HTI** | **Normoxia** | **Hypoxia** | **HTI** | **Normoxia** | **Hypoxia** | **HTI** | **Normoxia** | **Hypoxia** | **HTI** |
| 2-Want | 0.384 | 0.239 | 0.62 | 0.030 | 0.028 | 0.92 | 0.766 | 0.654 | 0.85 | 0.094 | 0.070 | 0.75 |
| 23-GR | 0.278 | 0.195 | 0.70 | 0.033 | 0.021 | 0.63 | 1.482 | 0.861 | 0.58 | 0.094 | 0.072 | 0.77 |
| 37-Test | 0.583 | 0.266 | 0.46 | 0.043 | 0.031 | 0.71 | 1.998 | 0.603 | 0.30 | 0.161 | 0.074 | 0.46 |
| 42-Test | 0.347 | 0.336 | 0.97 | 0.058 | 0.052 | 0.89 | 0.732 | 0.691 | 0.94 | 0.099 | 0.095 | 0.97 |
| 59-ALC | 0.349 | 0.182 | 0.52 | 0.049 | 0.028 | 0.56 | 0.931 | 0.558 | 0.60 | 0.069 | 0.074 | 1.07 |
| Amachuha | 0.525 | 0.369 | 0.70 | 0.056 | 0.022 | 0.39 | 1.524 | 0.532 | 0.35 | 0.052 | 0.071 | 1.38 |
| Atlas | 0.254 | 0.233 | 0.92 | 0.043 | 0.033 | 0.77 | 1.935 | 0.648 | 0.33 | 0.090 | 0.089 | 0.99 |
| Black quinoa | 0.487 | 0.364 | 0.75 | 0.031 | 0.034 | 1.11 | 0.847 | 0.455 | 0.54 | 0.128 | 0.075 | 0.58 |
| Cahuil | 0.632 | 0.243 | 0.38 | 0.055 | 0.030 | 0.54 | 1.230 | 0.641 | 0.52 | 0.125 | 0.072 | 0.57 |
| Chadmo | 0.407 | 0.179 | 0.44 | 0.064 | 0.022 | 0.34 | 1.872 | 0.892 | 0.48 | 0.134 | 0.086 | 0.64 |
| EDK4 | 0.364 | 0.327 | 0.90 | 0.035 | 0.028 | 0.80 | 1.283 | 0.726 | 0.57 | 0.187 | 0.070 | 0.38 |
| G13 | 0.444 | 0.361 | 0.81 | 0.061 | 0.028 | 0.46 | 1.895 | 1.429 | 0.75 | 0.209 | 0.085 | 0.41 |
| G18 | 0.398 | 0.352 | 0.88 | 0.039 | 0.028 | 0.74 | 1.116 | 0.604 | 0.54 | 0.160 | 0.073 | 0.46 |
| Isluga | 0.410 | 0.289 | 0.71 | 0.068 | 0.032 | 0.48 | 2.142 | 0.623 | 0.29 | 0.263 | 0.096 | 0.36 |
| Leucaboldo | 0.366 | 0.165 | 0.45 | 0.076 | 0.031 | 0.41 | 1.246 | 0.957 | 0.77 | 0.090 | 0.100 | 1.11 |
| NL-6 | 0.587 | 0.365 | 0.62 | 0.058 | 0.054 | 0.92 | 1.401 | 0.473 | 0.34 | 0.133 | 0.063 | 0.48 |
| Puno | 0.374 | 0.327 | 0.88 | 0.038 | 0.036 | 0.96 | 1.131 | 0.960 | 0.85 | 0.096 | 0.087 | 0.91 |
| Q1 | 0.894 | 0.297 | 0.33 | 0.086 | 0.038 | 0.44 | 1.315 | 0.445 | 0.34 | 0.110 | 0.083 | 0.75 |
| Q4 | 0.540 | 0.339 | 0.63 | 0.075 | 0.045 | 0.60 | 0.980 | 0.467 | 0.48 | 0.116 | 0.066 | 0.57 |
| Q4bias | 0.384 | 0.252 | 0.66 | 0.071 | 0.048 | 0.67 | 1.443 | 1.316 | 0.91 | 0.122 | 0.097 | 0.80 |
| Q5 | 0.319 | 0.151 | 0.48 | 0.059 | 0.024 | 0.40 | 1.334 | 0.672 | 0.50 | 0.113 | 0.080 | 0.71 |
| Real 1 x ruy 937 | 0.406 | 0.331 | 0.81 | 0.047 | 0.049 | 1.05 | 1.070 | 0.926 | 0.86 | 0.091 | 0.045 | 0.50 |
| Red | 0.373 | 0.166 | 0.44 | 0.051 | 0.028 | 0.55 | 1.543 | 0.617 | 0.40 | 0.052 | 0.068 | 1.30 |
| Riobamba | 0.564 | 0.235 | 0.42 | 0.056 | 0.028 | 0.50 | 1.617 | 0.763 | 0.47 | 0.075 | 0.087 | 1.16 |
| Ru2 | 0.465 | 0.331 | 0.71 | 0.077 | 0.046 | 0.59 | 1.747 | 0.680 | 0.39 | 0.116 | 0.067 | 0.58 |
| Sayaña | 0.533 | 0.181 | 0.34 | 0.047 | 0.026 | 0.54 | 1.494 | 0.486 | 0.33 | 0.125 | 0.058 | 0.47 |
| Surami x ruy 937 | 0.570 | 0.420 | 0.74 | 0.063 | 0.043 | 0.68 | 1.231 | 1.105 | 0.90 | 0.121 | 0.092 | 0.76 |
| Unknown | 0.427 | 0.189 | 0.44 | 0.075 | 0.025 | 0.34 | 1.209 | 0.750 | 0.62 | 0.096 | 0.084 | 0.87 |
