# Supplementary material for: Genotypic variation and root traits associated with hypoxia tolerance in quinoa (Chenopodium quinoa Willd.)
Source: PLoS One. 2026 Nov 3;21(11):e0361654. doi: 10.1371/journal.pone.0361654 (PMC13730327; doi:10.1371/journal.pone.0361654)
Supplement: Supplementary file 7 [file pone.0361654.s007.docx]

**Dear Editors,**

**Dear Reviewers**

Firstly, let us give our gratitude for your valuable recommendations

Following your recommendations and the reviewer’s comments, we have revised the manuscript accordingly (revisions are highlighted in track change).

We have also revised the title of the manuscript to better reflect its scope, focus, and main findings. The title has been changed from “Integrative Phenotyping, Multivariate Analyses, and Machine Learning Prediction for Quinoa (Chenopodium quinoa Willd.) under Hypoxia” to “Genotypic variation and root traits associated with hypoxia tolerance in Quinoa (Chenopodium quinoa Willd.)”.

Below are our responses to reviewer´s specific comments.

***Thanks so much again for your support !***

Sincerely yours,

**Nguyen Van Loc**

**Response to Reviewers — PONE-D-26-29301R1 (R2):**

**0. Change in Title of manuscripts and Correction of the deposited data file (not a reviewer comment)**

**Change the title 1:** Thank you for the opportunity to revise our manuscript. To better align the title with the actual scope and main findings of the study, we have revised the manuscript title from:

**“Integrative Phenotyping, Multivariate Analyses, and Machine Learning Prediction for Quinoa (*Chenopodium quinoa* Willd.) under Hypoxia”**

to: **“Genotypic variation and root Traits associated with hypoxia tolerance in Quinoa (*Chenopodium quinoa* Willd.)”**

The revised title more accurately reflects the primary focus of our study, particularly the evaluation of genotypic variation in hypoxia responses and the identification of root traits associated with hypoxia tolerance. We believe that the new title is more concise, biologically informative, and representative of the major findings and scientific contribution of the manuscript.

**-- Correction of data:** The S1 data file submitted at R1 contained, for most traits, we recheked and found some cells with a decimal-shift errors. So we have corrected it and then every tables and figures was regenerated from it.

**Changes made.**

- **Tables 1–6, Figs 2, 3, 4, 7, 8:** regenerated

**1. Academic Editor — literature precedent for the multi-factorial approach**

**Comment.** "…providing sufficient justification from the reputable, published scientific literature, regarding appropriateness of your experimental design and analytical approach… a sufficient number of examples be provided from other, reputable, published studies where multi-factorial analyses were applied successfully in conducting this type of analysis."

**Response.** No published application of GGE biplot or AMMI analysis to a single-factor, two-level controlled experiment was found. Gauch, Piepho and Annicchiarico (2008, Crop Science 48:866–889) and Forkman and Piepho (2014, Biometrics 70:639–647) define both methods for multi-environment yield trials; every crop application located uses three or more environments. The GGE/AMMI analysis is removed and replaced by PERMANOVA and MANOVA, for which two-level stress designs with correlated traits have precedent (Chaouachi et al. 2023, AoB PLANTS 15:plad022; Iftikhar-ud-Din and Hayat 2021, Sarhad J. Agric. 37:1250–1259; Asfaw et al. 2017, Front. Plant Sci. 8:733).

**Changes made.**

- **See item 2.**

**2. Reviewer 2, Comments 1–2 — GGE/AMMI, which-won-where and stability analyses**

**Comment.** "…the authors apply methods such as GGE biplots and AMMI analysis, which were primarily developed for multi-environment trials… This experiment clearly does not represent such a multi-environment trial." "…the main analysis should be the ANOVA/mixed-model analysis… an additional analysis could be included that accounts for the strong correlations among the measured phenotypic traits, for example, MANOVA or a similar multivariate mixed-model approach such as PCA… with only two environments… the multiplicative interaction is limited to a single interaction dimension… Figs. 5 and 6 being essentially identical."

**Response.** The GGE and AMMI analyses, Figs 5 and 6 and the text based on them are removed. With two environment levels the environment-centred GGE matrix has rank two, so PC1 + PC2 = 100% by construction. The multivariate analysis is now a PERMANOVA of the 19 traits of Table 2 (standardized, Euclidean distance, replicate + genotype × oxygen regime, 999 permutations): genotype 37.1%, oxygen regime 30.0%, interaction 18.8%, replicate 0.4%, residual 13.7% of the multivariate variation (all p = 0.001). A classical MANOVA on the same model gives Pillai's trace 0.991 for oxygen regime, 11.22 for genotype and 9.51 for the interaction (all p < 0.001). The hierarchical clustering (Fig 7) is retained as trait-profile clustering.

**Changes made.**

- **Abstract, para. 12:** delete "GGE biplot and cluster analyses identified Isluga … low genotype x environment interaction." Suggested replacement: "Permutational multivariate analysis of variance across 19 traits attributed 37.1% of overall phenotypic variation to genotype, 30.0% to oxygen regime, and 18.8% to their interaction (all p = 0.001), and hierarchical clustering on hypoxia-tolerance indices resolved three genotype groups with contrasting trait profiles. Under hypoxia, Amachuha and Isluga produced the highest individual yields, followed by EDK4 and Chadmo."
- **Introduction, para. 18:** "assess genotype adaptation and stability using the genotype main effect plus genotype by environment interaction (GGE) biplot and cluster analyses" → "characterize genotype × oxygen-regime effects across correlated traits using multivariate analysis of variance and hierarchical clustering".
- **Methods, paras. 35–41:** delete the "AMMI and GGE biplot analysis" subsection (equations 2–3). Add after "Hierarchical clustering and principal component analysis": "Multivariate analysis of variance. To account for correlations among the measured traits, a permutational multivariate analysis of variance (PERMANOVA; Anderson 2001) was fitted to the 19 traits in Table 2 with the vegan package (adonis2; Euclidean distance on standardized traits; terms added sequentially as replicate + genotype × environment; 999 permutations). A classical MANOVA (Pillai's trace) on the same model was computed for comparison."
- **Results, paras. 57–60:** delete "AMMI and GGE biplot analysis" and its three paragraphs. Insert after "Analysis for variance": "Multivariate analysis of variance. Across the 19 traits jointly, PERMANOVA attributed 37.1% of total phenotypic variation to genotype, 30.0% to oxygen regime, and 18.8% to the genotype × oxygen-regime interaction (all p = 0.001; replicate 0.4%, residual 13.7%; Table 4). The classical MANOVA confirmed all three effects (Pillai's trace: oxygen regime 0.991, genotype 11.22, interaction 9.51; all p < 0.001; Table 5)."
- **Results, para. 62:** end the clustering paragraph with "…indicates substantial variation in multi-trait response profiles among the evaluated germplasm" (delete "diverse adaptation patterns").
- **Discussion, para. 69:** delete the first five sentences ("The clustering pattern was consistent with the GGE biplot results" … "acceptable stability (Fig. 6)"). In the sentence on G18, end with "…is identified by both the yield comparison (Fig. 2) and the clustering analysis (Fig. 7) as a promising genetic resource".
- **Conclusion, para. 74:** delete "GGE biplot and cluster analyses identified Isluga … low genotype x environment interaction." Suggested replacement: "Genotype, oxygen regime and their interaction each explained a substantial share of multivariate phenotypic variation, and hierarchical clustering on hypoxia-tolerance indices distinguished three trait-profile groups; Amachuha and Isluga produced the highest individual yields under hypoxia."
- **Figures and tables:** remove Figs 5 and 6 and their captions (paras. 182–183); Fig 7 → Fig 5, Fig 8 → Fig 6. New Table 4 (PERMANOVA) and Table 5 (MANOVA): folder 05/Table4-5_PERMANOVA_MANOVA.
- **Terminology (also Reviewer 3, Comment 1):** "environment" → "oxygen regime" where the two treatments are meant; "genotype × environment interaction" → "genotype × oxygen-regime interaction"; delete "stability", "stable", "adaptation", "adapted", "ideal genotype" and "mega-environment" wherever they came from the GGE analysis.
- **S1 File:** R/PERMANOVA_MANOVA.R; the GGE scripts are kept under R/withdrawn/.

**3. Reviewer 2, Comments 3–4 and 6 — machine learning with 28 genotypes, cross-validation, population structure; ecotypes in Table 1**

**Comment.** "…the analyses using ML models given the very small number of genotypes (n = 28) needs to be treated with caution… If the same cross-validation procedure is used for both model/hyperparameter selection and estimation of predictive performance, the estimated prediction accuracy can be optimistically biased (Varma & Simon 2006)." "…the unknown genetic relationships among the genotypes and the apparent population structure represented by the highland and lowland ecotypes are major concerns… A simple test… would be to train the models using only the highland accessions and predict the lowland accessions, and vice versa… the goal of the intended prediction should be clearly defined." "In table 1, the genotypes should be assigned to lowland and highland ecotypes."

**Response.** Each genotype is assigned to the highland (13) or lowland (15) ecotype, and an Ecotype column is added to Table 1. The R1 random 80/20 split of observations placed every test genotype in the training set as well, so its accuracy reflected genotype recognition. The nine models were re-evaluated with three genotype-level designs — leave-one-genotype-out, leave-one-cluster-out (the three clusters of Fig 7) and the cross-ecotype test requested (train on highland, predict lowland, and the reverse) — with hyperparameters selected inside each training set by five-fold cross-validation grouped by genotype, and compared with a reference that uses no trait: the mean yield of the training observations under the same oxygen regime. No model reached that reference in any genotype-level design (best model R² 0.125 against 0.185 leave-one-genotype-out; 0.088 against 0.189 leave-one-cluster-out; 0.047 against 0.231 cross-ecotype), and within a regime the predictions did not rank genotypes. The prediction target — ranking genotypes not used in training for yield within a regime — is stated in Methods and is not achieved. The clusters of Fig 7 follow ecotype (Cluster I: 10 of 13 highland genotypes; Clusters II and III: 13 of 15 lowland genotypes; Fisher's exact test p = 0.0016).

**Changes made.**

- **Table 1:** add a column "Ecotype" (highland: 37-Test, 59-ALC, Amachuha, Cahuil, Chadmo, Isluga, Leucaboldo, Puno, Red, Riobamba, Ru2, Sayaña, Unknown; lowland: 2-Want, 23-GR, 42-Test, Atlas, Black quinoa, EDK4, G13, G18, NL-6, Q1, Q4, Q4bias, Q5, Real1 × Ruy937, Surami × Ruy937) and state the criterion of the classification in the table note.
- **Methods, para. 44:** replace "The dataset was randomly divided into training (80%) and testing (20%) subsets…" with: "The models were built to test whether the measured seedling traits can rank genotypes not used in training for individual yield within the same oxygen regime. Because each genotype contributed 6 observations (2 oxygen regimes × 3 replicates), models were evaluated at the genotype level in three ways: (i) leave-one-genotype-out — for each of the 28 genotypes in turn, all of its observations were withheld, the model was trained on the remaining 27 genotypes and yield was predicted for the withheld genotype; (ii) leave-one-cluster-out — each of the three genotype clusters of Fig. 7 was withheld in turn; (iii) cross-ecotype — models were trained on the 13 highland genotypes and tested on the 15 lowland genotypes, and vice versa. Hyperparameters were selected within each training set by five-fold cross-validation with folds grouped by genotype, so that model selection and performance estimation never shared data (Varma and Simon 2006). Each design was compared with a reference predictor that uses no trait, the mean yield of the training observations under the same oxygen regime, and with a random 80/20 split of individual observations." Delete "To ensure robustness… repeated k-fold cross-validation" (para. 45).
- **Results, para. 64:** replace the paragraph with: "Predictive accuracy for genotypes excluded from training was low for all nine models, and none exceeded the regime-mean reference (Fig. 8; Table 6). Under leave-one-genotype-out validation the best model, LASSO, reached R² = 0.125 (r = 0.36) against 0.185 for the reference; random forest reached 0.105. Withholding whole genotype clusters gave R² ≤ 0.09 (reference 0.19), and training on one ecotype and testing on the other gave pooled R² ≤ 0.05 (reference 0.23). Within a regime, observed and predicted yields of withheld genotypes showed no positive correlation. By contrast, a random split of individual observations, in which every test-set genotype also appeared in the training set, gave R² = 0.32 for random forest and 0.25 for KNN."
- **Table 6 (new):** the table below; file 05/Fig8_ML/Table_comparison_all_four.csv and Table_regime_mean_baseline.csv.
- **Fig 8:** observed versus predicted yield of withheld genotypes under leave-one-genotype-out validation, one panel per model (05/Fig8_ML/ML_LOGO_observed_vs_predicted); a single-panel random-forest version is provided.
- **Discussion, para. 69:** replace "Highland genotypes such as Isluga and Sayaña clustered with stress-tolerant materials, whereas Chilean lowland genotypes such as NL-6 and Cahuil formed distinct groups, consistent with the reported eco-geographical differentiation of quinoa populations [26,27,38,39]." with "The clusters also followed ecotype: Cluster I contained 10 of the 13 highland genotypes, and Clusters II and III 13 of the 15 lowland genotypes (Fisher's exact test, p = 0.002), consistent with the eco-geographical differentiation of quinoa populations [26,27,38,39]." (Cahuil is a highland genotype under the classification used.)
- **Abstract, Discussion para. 70, Conclusion:** delete the sentences on KNN, GBM and random forest accuracy (r = 0.77–0.86, R² = 0.56–0.71). Suggested sentence: "Under genotype-level validation (leave-one-genotype-out, leave-one-cluster-out and highland-versus-lowland cross-ecotype splits), no model predicted the individual yield of genotypes excluded from training better than the mean yield of the training genotypes under the same oxygen regime, so trait-based yield prediction does not transfer to unseen quinoa genotypes." References 40–45 become uncited if the paragraph is shortened.
- **Limitations, para. 72:** add that the ecotype assignment is not marker-based and that the machine-learning results are exploratory: no model predicted the yield of genotypes outside the training set, or across ecotypes, better than the regime mean.
- **S1 File:** python/ML_leave_cluster_out.py, ML_leave_one_genotype_out.py, ML_cross_ecotype.py, ML_baselines.py; data/genotype_ecotype.csv.

*Table 6 (proposed). Predictive accuracy for individual yield under four validation designs; nine models and the no-trait reference.*

| **Model** | **Leave-one-genotype-out R² (r)** | **Leave-one-cluster-out R²** | **Cross-ecotype R² (r)** | **Random row split R²** |
| --- | --- | --- | --- | --- |
| Regime mean of the training observations (no traits) | 0.185 (0.433) | 0.189 | 0.231 (0.481) | 0.156 |
| LASSO | 0.125 (0.363) | 0.088 | -0.003 (-0.032) | 0.159 |
| Random forest | 0.105 (0.358) | 0.063 | 0.036 (0.273) | 0.324 |
| Elastic Net | 0.072 (0.289) | 0.070 | 0.033 (0.220) | 0.062 |
| Gradient boosting | 0.062 (0.304) | 0.088 | -0.089 (0.094) | 0.175 |
| KNN | 0.021 (0.327) | -0.040 | -0.103 (0.199) | 0.250 |
| Ridge | 0.015 (0.256) | 0.030 | 0.031 (0.280) | 0.161 |
| Linear SVM | 0.008 (0.285) | -0.020 | 0.047 (0.311) | 0.119 |
| OLS | -0.186 (0.200) | -0.243 | -0.404 (0.258) | 0.082 |
| Multilayer perceptron | -1.329 (-0.032) | -1.191 | -1.143 (0.083) | -0.322 |

**4. Reviewer 2, Comment 12 — HTI reporting**

**Comment.** "Lines 212ff: The HTI is not consistently reported in this section. The usefulness of this metric is not clear as it corresponds in an inverse manner with the percent changes reported as well."

**Response.** The Results quoted, for each trait, a percentage change computed from the panel means, while Tables 3–6 tabulate the per-genotype ratio HTI; a mean of ratios differs from the ratio of means, so the two sets of numbers did not agree. The revised text reports one quantity, the mean of the per-genotype HTI, and gives a percentage change only as (1 − HTI) × 100.

**Changes made.**

- **Results, paras. 50–53:** replace the in-text values with the means of the per-genotype HTI from the regenerated tables (Table 3 panel summary, folder 05/Table3-6_HTI): RL1 0.61, RL2 0.70, RSA1 0.75, RSA2 0.70, RD1 1.28, RD2 1.01, PH1 0.69, PH2 0.73, NoL1 0.90, NoL2 0.82, LA1 0.53, LA2 0.64, SPAD1 0.92, SPAD2 0.95, SDW1 0.63, SDW2 0.56, RDW1 0.64, RDW2 0.74.
- **Discussion, para. 67:** "average HTI values of 0.57 and 0.58" → "0.61 and 0.70".
- **Abstract, para. 12:** re-read the percentage figures from the same table if they are kept.
- **New Table 3 (proposed):** panel means under normoxia and hypoxia and mean HTI per trait (05/Table3-6_HTI/HTI_panel_summary.csv); R1 Tables 3–6 can then move to Supporting Information (Comment 7).

**5. Reviewer 2, Comment 13 — "completely altered"**

**Comment.** "Line 230: 'completely altered' – what exactly does this mean? This is a qualitative but not a quantitative description of the differences."

**Response.** The statement is replaced by the rank correlation between the genotype rankings under the two regimes: Spearman rho = 0.25 (p = 0.21, n = 28), with a median shift of 6 rank positions. Amachuha ranks first under hypoxia (5.26 g plant⁻¹) and second under normoxia (6.20 g); Chadmo, first under normoxia (6.62 g), ranks fourth under hypoxia (4.55 g), behind Isluga (4.89 g) and EDK4 (4.57 g).

**Changes made.**

- **Results, para. 54:** replace "Although hypoxia reduced IY across genotypes, the ranking of genotypes was not completely altered. Chadmo consistently produced the highest IY under both conditions…" with "Hypoxia reduced IY in all genotypes and reordered them: genotype rankings under normoxia and hypoxia were weakly correlated (Spearman rho = 0.25, p = 0.21), with a median shift of 6 rank positions. Amachuha was the highest-yielding genotype under hypoxia (5.26 g plant⁻¹) and second under normoxia (6.20 g), whereas Chadmo, the highest under normoxia (6.62 g), ranked fourth under hypoxia (4.55 g), behind Isluga (4.89 g) and EDK4 (4.57 g)."
- **Discussion, para. 68:** "Chadmo, G18, G13, Amachuha, and Ru2 achieved the highest individual yields under hypoxia" → the five highest under hypoxia are Amachuha, Isluga, EDK4, Chadmo, 42-Test (Fig 2).
- **Files:** 05/Fig2_nang_suat_LSD/IY_genotype_ranks.csv, IY_rank_correlation.csv, IY_boxplot_letters.csv.

**6. Reviewer 3, Comment 1 — terminology**

**Comment.** "…the study remains fundamentally a factorial genotype-by-treatment experiment rather than a conventional multi-environment trial. Terms such as 'adaptation,' 'stability,' 'environment,' and 'ideal genotype' should therefore be used cautiously."

**Response.** These terms came from the GGE analysis, which is removed (item 2). The experiment is described as a genotype × oxygen-regime experiment.

**Changes made.**

- **Whole text:** "environment" → "oxygen regime" for the two treatments; "genotype × environment interaction" → "genotype × oxygen-regime interaction"; delete "stability", "stable", "adaptation", "adapted", "ideal genotype", "mega-environment" (see item 2).

**7. Reviewer 3, Comment 2 — machine-learning results as exploratory**

**Comment.** "…the machine-learning results should be presented primarily as exploratory phenotype-based prediction rather than as evidence of robust predictive models ready for breeding deployment."

**Response.** Under genotype-level validation no model predicted the yield of genotypes excluded from training better than the regime mean (item 3). The revised text makes no predictive claim for untested genotypes.

**Changes made.**

- **See item 3:** Methods para. 44, Results para. 64, Table 6, Fig 8, and the sentences suggested for the Abstract, Discussion and Conclusion; Limitations state that the results are exploratory.

**Decisions for the corresponding author**

- Numbering of the revised manuscript: R1 Tables 3–6 to Supporting Information (S1–S4 Tables), new Table 3 = panel means and mean HTI, Table 4 = PERMANOVA, Table 5 = MANOVA, Table 6 = validation table; Figs 5–6 removed, Fig 7 → 5, Fig 8 → 6.
- Table 1 note: the criterion of the highland/lowland classification.
- Fig 2: replicate points in one neutral colour (as regenerated) or genotype colours.
- Fig 3: the correlation matrix pools both regimes (168 observations); eight of twelve coefficients quoted in the R1 text agree within 0.10 with the regenerated values, SDW2–IY (0.14 → 0.40), RD1–SDW1 (−0.24 → −0.40), RD1–TDW1 (−0.23 → −0.41) and RD2–RSA2 (−0.39 → +0.19) do not.
- Fig 4: the regenerated PCA (56 genotype × regime means, 20 traits) gives PC1 = 48.55%, PC2 = 9.80%; the R1 values (45.38%, 8.85%) cannot be obtained from the deposited file.
- Genotype spelling: one spelling for each genotype in Table 1, text, figures and data (e.g. Real1 × Ruy937, Surami × Ruy937, EDK4, Ru2).
- Three data points to check against the record sheets: Isluga yields more under hypoxia (4.89 g) than under normoxia (3.48 g); Black quinoa root length under hypoxia at 30 DAS (662 cm) is nearly double its normoxia value (364 cm); the R1 Abstract values 1.38/0.74 g are shoot dry weight after recovery (SDW2), not total dry weight.

**Files in S1 File behind the numbers**

| **Item** | **Script** | **Output** |
| --- | --- | --- |
| Corrected dataset | data/build_v2_rep1-3_corrected.py | data/Hypoxia_and_Normoxia_Raw_data_Code_v2_rep1-3_corrected.csv; corrections_v2_changelog.csv |
| Screen of the deposited file | data/audit/data_integrity_check_raw_csv.py | flagged_10x_cells.csv; replicate_duplication_by_trait.csv |
| Table 2 | R/ANOVA_mean_squares.R | outputs/ANOVA/ |
| Tables 3–6, new Table 3 | R/HTI_summaries.R | outputs/HTI/; HTI_panel_summary.csv |
| PERMANOVA, MANOVA | R/PERMANOVA_MANOVA.R | outputs/PERMANOVA/ |
| Fig 2, rank correlation | R/IY_boxplot_LSD.R | outputs/IY_boxplot/ |
| Fig 3 | R/Correlation_matrix.R | outputs/Correlation/ |
| Fig 4 | R/PCA_biplot.R | outputs/PCA/ |
| Fig 7, cluster × ecotype | R/HCPC_clustering.R | outputs/HCPC/ |
| Machine learning, four designs | python/ML_random_split.py; ML_leave_cluster_out, ML_leave_one_genotype_out, ML_cross_ecotype | outputs/ML_random_split, ML_leave_cluster_out, ML_leave_one_genotype_out, ML_cross_ecotype/ |
| Reference predictor | python/ML_baselines.py | outputs/ML_baselines/ |
