# Supplementary material for: Genotypic variation and root traits associated with hypoxia tolerance in quinoa (Chenopodium quinoa Willd.)
Source: PLoS One. 2026 Nov 3;21(11):e0361654. doi: 10.1371/journal.pone.0361654 (PMC13730327; doi:10.1371/journal.pone.0361654)
Supplement: Peer review history

# Peer review: Decision Letter 0

**DOI:** 10.1371/journal.pone.0361654.r001

**Authors:** Eric Jellen

7 Jul 2026

Dear Dr. Loc Nguyen,

Thank you for submitting your manuscript to PLOS ONE. After careful consideration, we feel that it has merit but does not fully meet PLOS ONE’s publication criteria as it currently stands. Therefore, we invite you to submit a revised version of the manuscript that addresses the points raised during the review process.

You will need to revise the manuscript, including formatting it correctly in line-numbered manuscript form, with a thorough revision of the English and clear explanations of the significance of the observations in the Discussion. Also, you should omit "genotypes" 22 and 27 as these are most likely heterogeneous/heterozygous segregating populations derived from wide crosses.

Please submit your revised manuscript by Sep 05 2026 11:59PM. If you will need more time than this to complete your revisions, please reply to this message or contact the journal office at plosone@plos.org. When you're ready to submit your revision, log on to https://www.editorialmanager.com/pone/ and select the 'Submissions Needing Revision' folder to locate your manuscript file.

- A letter that responds to each point raised by the academic editor and reviewer(s). You should upload this letter as a separate file labeled 'Response to Reviewers'.
- A marked-up copy of your manuscript that highlights changes made to the original version. You should upload this as a separate file labeled 'Revised Manuscript with Track Changes'.
- An unmarked version of your revised paper without tracked changes. You should upload this as a separate file labeled 'Manuscript'.

If you would like to make changes to your financial disclosure, please include your updated statement in your cover letter. Guidelines for resubmitting your figure files are available below the reviewer comments at the end of this letter.

If applicable, we recommend that you deposit your laboratory protocols in protocols.io to enhance the reproducibility of your results. Protocols.io assigns your protocol its own identifier (DOI) so that it can be cited independently in the future. For instructions see: https://journals.plos.org/plosone/s/submission-guidelines#loc-laboratory-protocols. Additionally, PLOS ONE offers an option for publishing peer-reviewed Lab Protocol articles, which describe protocols hosted on protocols.io. Read more information on sharing protocols at https://plos.org/protocols?utm_medium=editorial-email&utm_source=authorletters&utm_campaign=protocols.

As the corresponding author, your ORCID iD is verified in the submission system and will appear in the published article. PLOS supports the use of ORCID, and we encourage all coauthors to register for an ORCID iD and use it as well. Please encourage your coauthors to verify their ORCID iD within the submission system before final acceptance, as unverified ORCID iDs will not appear in the published article. *Only*  the individual author can complete the verification step; PLOS staff *cannot*  verify ORCID iDs on behalf of authors.

We look forward to receiving your revised manuscript.

Kind regards,

Eric Jellen, Ph.D.

Academic Editor

PLOS One

**Journal Requirements:**

When submitting your revision, we need you to address these additional requirements.

1. Please ensure that your manuscript meets PLOS ONE's style requirements, including those for file naming. The PLOS ONE style templates can be found at

https://journals.plos.org/plosone/s/file?id=wjVg/PLOSOne_formatting_sample_main_body.pdf and

https://journals.plos.org/plosone/s/file?id=ba62/PLOSOne_formatting_sample_title_authors_affiliations.pdf

2. Please include a complete copy of PLOS’ questionnaire on inclusivity in global research in your revised manuscript. Our policy for research in this area aims to improve transparency in the reporting of research performed outside of researchers’ own country or community. The policy applies to researchers who have travelled to a different country to conduct research, research with Indigenous populations or their lands, and research on cultural artefacts. The questionnaire can also be requested at the journal’s discretion for any other submissions, even if these conditions are not met. Please find more information on the policy and a link to download a blank copy of the questionnaire here: https://journals.plos.org/plosone/s/best-practices-in-research-reporting. Please upload a completed version of your questionnaire as Supporting Information when you resubmit your manuscript.

3. Please note that PLOS One has specific guidelines on code sharing for submissions in which author-generated code underpins the findings in the manuscript. In these cases, we expect all author-generated code to be made available without restrictions upon publication of the work. Please review our guidelines at https://journals.plos.org/plosone/s/materials-and-software-sharing#loc-sharing-code and ensure that your code is shared in a way that follows best practice and facilitates reproducibility and reuse.

4. Thank you for stating the following financial disclosure:

“This research was supported by the National Foundation For Science and Technology Development (NAFOSTED) program of Vietnam on “Study on the waterlogging tolerance of Quinoa (Chenopodium quinoa Willd.) for development of Climate-Resilient Crops in Vietnam” from 2025-2028 (grant No. 106.03-2025.03) awarded to Vietnam National University of Agriculture (VNUA).”

Please state what role the funders took in the study.  If the funders had no role, please state: "The funders had no role in study design, data collection and analysis, decision to publish, or preparation of the manuscript."

If this statement is not correct you must amend it as needed.

Please include this amended Role of Funder statement in your cover letter; we will change the online submission form on your behalf.

5. Please note that funding information should not appear in any section or other areas of your manuscript. We will only publish funding information present in the Funding Statement section of the online submission form. Please remove any funding-related text from the manuscript.

6. In the online submission form you indicate that your data is not available for proprietary reasons and have provided a contact point for accessing this data. Please note that your current contact point is a co-author on this manuscript. According to our Data Policy, the contact point must not be an author on the manuscript and must be an institutional contact, ideally not an individual. Please revise your data statement to a non-author institutional point of contact, such as a data access or ethics committee, and send this to us via return email. Please also include contact information for the third party organization, and please include the full citation of where the data can be found.

7. Please include your full ethics statement in the ‘Methods’ section of your manuscript file. In your statement, please include the full name of the IRB or ethics committee who approved or waived your study, as well as whether or not you obtained informed written or verbal consent. If consent was waived for your study, please include this information in your statement as well.

8. Please include captions for your Supporting Information files at the end of your manuscript, and update any in-text citations to match accordingly. Please see our Supporting Information guidelines for more information: http://journals.plos.org/plosone/s/supporting-information.

9. If the reviewer comments include a recommendation to cite specific previously published works, please review and evaluate these publications to determine whether they are relevant and should be cited. There is no requirement to cite these works unless the editor has indicated otherwise.

**Additional Editor Comments:**

I am recommending resubmission of the manuscript after major revisions to most aspects of the manuscript. Especially, look closely at the detailed comments of Reviewer 2 and indicate how you addressed each comment. This includes a thorough description of the genotype panel and how this panel is representative of the range of diversity in quinoa. Also, you should probably exclude "genotypes" 22 and 27 from your analyses, since these were most likely heterogeneous/heterozygous populations derived from wide interspecific crosses and not inbred lines. You should also verify with your Argentine co-authors the identity of genotype 28.

Since Reviewer 1 did not review the manuscript favorably, I will likely recommend a different reviewer after it is resubmitted, so please expect that you will subsequently have to address a second round of reviewer comments - at least, assuming that you satisfy the current Reviewer 2 in your resubmission.

[Note: HTML markup is below. Please do not edit.]

Reviewers' comments:

Reviewer's Responses to Questions

**Comments to the Author**

1. Is the manuscript technically sound, and do the data support the conclusions?

Reviewer #1: Partly

Reviewer #2: Partly

**********

2. Has the statistical analysis been performed appropriately and rigorously?

Reviewer #1: No

Reviewer #2: Yes

**********

3. Have the authors made all data underlying the findings in their manuscript fully available?

The PLOS Data policy

Reviewer #1: No

Reviewer #2: No

**********

4. Is the manuscript presented in an intelligible fashion and written in standard English?

Reviewer #1: No

Reviewer #2: Yes

**********

Reviewer #1: The manuscript addresses an important topic and includes a valuable dataset with potential implications for quinoa breeding under hypoxia stress. However, substantial revisions are required to improve methodological transparency, strengthen the organization of the Results and Discussion sections, clarify the experimental design, and enhance the reproducibility of the statistical and machine-learning analyses. These improvements would considerably increase the scientific quality and impact of the manuscript.

Reviewer #2: The manuscript describes the phenotypic responses of 28 quinoa genotypes to hypoxia and combines physiological measurements with multivariate analyses and machine-learning approaches for predicting individual seed yield. The manuscript addresses an important aspect of quinoa adaptation to waterlogging stress. The experiments generally reflect the current state of the art, and the statistical analyses are comprehensive.

The manuscript addresses an important topic and presents a suitable dataset to study hypoxia. However, the authors should strengthen the biological interpretation of the results, justify the choice of plant material more thoroughly, discuss the limitations imposed by the small sample size, and clarify the practical relevance of the machine-learning analyses. A careful revision with respect to typing errors is also recommended.

The following comments should be addressed in a revised version.

Major comments

1. Selection of the 28 genotypes

The selection of the 28 genotypes used in the study should be justified in greater detail. The manuscript states that they represent different genetic backgrounds, but it is not clear whether they originate from lowland or highland quinoa. More information on the origin, adaptation, and selection criteria of these genotypes should be provided (i.e., whether they are varieties, genebank accessions, progeny of crosses). Since no passport information is available, it would be useful to explain why these particular genotypes were chosen and how representative they are of quinoa diversity.

2. Statistical consequences of the limited sample size

The relatively small number of genotypes raises statistical concerns, particularly because a large number of machine-learning models are evaluated. It is likely that the evaluated material contains considerable population structure, with genetically related genotypes originating from similar breeding backgrounds or geographic regions. This may substantially inflate the prediction accuracies obtained by the machine-learning models, despite the cross-validation used. The authors should discuss why such a small number of genotypes nevertheless resulted in extremely high prediction accuracies. It should also be discussed whether these high accuracies are partly explained by population structure rather than genuine predictive ability. If genetic marker data are available for these materials, they could help address this question.

Specific comments

1. The manuscripts lack page and line numbers, which should be included in a revised version

2. At the end of the first paragraph of the introduction, the authors should provide specific examples of previous studies investigating hypoxia tolerance in quinoa or other crops. This would better motivate the objectives of the present study.

3. The introduction would also benefit from a clearer explanation of why this study provides a "comprehensive framework" for evaluating hypoxia tolerance.

4. Materials and Methods: The experimental procedures should be described in greater detail. It is currently not entirely clear how the plants were propagated before transfer into the hydroponic system and whether seed yield was measured from plants kept in the hydroponic system or put into pots with soil.

5. Materials and Methods: In the spirit of reproducible research, the analysis scripts should be made available.

6. Results section: The abbreviations 'HTI', 'RSA' should be explained before their first use.

7. In several sections, the contents of the tables and figures are repeated almost verbatim in the text. Instead, the text in Results should focus on the key findings and trends rather than restating all numerical values. This is particularly noticeable in the sections describing the effects of hypoxia and in the correlation analysis.

8. AMMI and GGE analyses: It is remarkable that the first two principal components explain 100% of the genotype-by-environment variation. Although this is mathematically possible with only two environments, this is unusual and should be explained explicitly to avoid confusing readers.

9. Discussion: The term "upland crop" should be explained more clearly.

10. Large parts of the Discussion simply repeat the Results. Instead, the authors should place their findings into the context of previous studies and discuss their biological implications.

11. The purpose of the machine-learning prediction analysis is not entirely clear because no genetic marker information is included. The authors should explain more clearly what additional biological insight is obtained from these prediction models beyond demonstrating predictive performance. In particular, the authors should discuss whether these prediction models would also be applicable in segregating breeding populations derived from crosses between contrasting parents, where traits like response to hypoxia are genetically segregating. Could these models be useful for selection in breeding programs?

12. The final part of the Discussion should also address why the nonlinear machine-learning methods substantially outperform the linear models. Which biological sources of nonlinearity are likely responsible for these differences?

13. The Conclusion is largely repetitive and summarizes the Results and Discussion without providing additional perspectives. It could be substantially shortened.

14: Figure 2. The figure is rather noisy. Genotypes should be reordered from low to high grain yield values as the statistical tests are then better ot interprete.

15: Figures 4 and 5. The two different GGE biplots should be introduced and briefly explained in the Materials and Methods section before they are presented in the Results.

16. Figure 6. The hierarchical cluster analysis should be discussed in the context of the putative genetic similarity among the genotypes. It is noteworthy that several highland accessions appear within the same clusters, suggesting that the clustering may partly reflect genetic differentiation between lowland and highland quinoa. This point should be discussed further.

**********

what does this mean?). If published, this will include your full peer review and any attached files.

If you choose “no”, your identity will remain anonymous but your review may still be made public.

**Do you want your identity to be public for this peer review?**  For information about this choice, including consent withdrawal, please see our Privacy Policy

Reviewer #1: No

Reviewer #2: No

**********

[NOTE: If reviewer comments were submitted as an attachment file, they will be attached to this email and accessible via the submission site. Please log into your account, locate the manuscript record, and check for the action link "View Attachments". If this link does not appear, there are no attachment files.]

To ensure your figures meet our technical requirements, please review our figure guidelines: https://journals.plos.org/plosone/s/figures

You may also use PLOS’s free figure tool, NAAS, to help you prepare publication quality figures: https://journals.plos.org/plosone/s/figures#loc-tools-for-figure-preparation.

NAAS will assess whether your figures meet our technical requirements by comparing each figure against our figure specifications.

Attachment Submitted filename: PONE-D-26-29301_FL.pdf

# Author comment: Author response to Decision Letter 1

**DOI:** 10.1371/journal.pone.0361654.r002

29 Jul 2026

Dear Editor of Plos One

Dear Reviewers

Firstly, let us give our gratitude for your valuable recommendations

Following your recommendations and the reviewer’s comments, we have revised the manuscript accordingly (revisions are highlighted in yellow). Below are our responses to reviewer´s specific comments.

Thanks so much again for your support !

Sincerely yours,

Authors,

Authors’ revision for comments

Editor’s comments: You will need to revise the manuscript, including formatting it correctly in line-numbered manuscript form, with a thorough revision of the English and clear explanations of the significance of the observations in the Discussion. Also, you should omit "genotypes" 22 and 27 as these are most likely heterogeneous/heterozygous segregating populations derived from wide crosses.

Authors’ responses to Editor’s comments: Thank you for your valuable comments. We have carefully revised the manuscript in accordance with all reviewers' comments, including formatting the manuscript with line numbers, improving the English throughout, and strengthening the Discussion to better explain the significance of our findings.

Regarding genotypes 22 and 27, we respectfully retained them because they are stable F8 recombinant inbred lines (RILs) that have been widely used in previous studies investigating quinoa responses to abiotic stresses, making them valuable and reliable genetic materials. To avoid any misunderstanding, we have revised the Materials and Methods section to clearly describe their genetic background.

Authors’ Responses to the Reviewers’ Comments

Reviewer 1:

Gerneral comments#: Integrative Phenotyping, Multivariate Analyses, and Machine Learning Prediction for Quinoa (Chenopodium quinoa Willd.) under Hypoxia The manuscript addresses an important topic and includes a valuable dataset with potential implications for quinoa breeding under hypoxia stress. However, substantial revisions are required to improve methodological transparency, strengthen the organization of the Results and Discussion sections, clarify the experimental design, and enhance the reproducibility of the statistical and machine-learning analyses. These improvements would considerably increase the scientific quality and impact of the manuscript.

Response to General Comments#

We sincerely thank the reviewer for the constructive and valuable comments. We appreciate your recognition of the importance of our study and the potential value of the dataset for quinoa breeding under hypoxia stress.Following your suggestions, we have carefully revised the manuscript to improve methodological transparency, clarify the experimental design, reorganize the Results and Discussion sections, and provide more detailed descriptions of the statistical and machine-learning analyses to enhance reproducibility. All comments have been addressed individually in the following point-by-point responses, and the corresponding revisions have been incorporated into the revised manuscript:

Comment #1: Abstract 1. The abbreviation RSA is introduced in the sentence "Correlation analysis revealed that RL and RSA were positively associated with IY" without being previously defined. Please provide the full term at its first occurrence before using the abbreviation.

Authors’ response to comment #1: Thank you for your careful observation. We have revised the Abstract by defining the abbreviation RSA (root surface area) at its first occurrence before using the abbreviation throughout the text.

Comment #2. In the phrase "machine-learning approaches, particularly KNN, RF, SVM, and GBM," the abbreviations are introduced without first stating their full names. Please spell out the full names of these algorithms at their first mention, followed by the corresponding abbreviations in parentheses.

Authors’ response to comment #2: Thank you for pointing this out. We have revised the Abstract to spell out the full names of all machine learning algorithms at their first occurrence, followed by their abbreviations in parentheses, to improve clarity and readability (Lines 30-33 Page 2 in revision manuscript) as following:

Furthermore, on an independent held-out test set, machine-learning models based on tree- and instance-based learning—K-nearest neighbors, gradient boosting machine, and random forest—predicted individual yield more accurately (r = 0.77–0.86, R² = 0.56–0.71) than conventional linear-regularized models and the other nonlinear approaches evaluated (r = 0.47–0.51, R² = 0.11–0.23).

Comment #3. The abstract should present the main findings using quantitative results rather than only qualitative statements. Please include key numerical values (e.g., percentage reductions, correlation coefficients, model performance metrics, or explained variance) to highlight the principal outcomes of the study.

Authors’ response to comment #3: Thank you very much for your valuable comment. We have carefully checked the manuscript and revised it accordingly (Lines 21-25 and Line 30-33 of Page 2).

Comment #4. Please revise the keywords. They should be arranged in alphabetical order and should not duplicate terms already included in the title. Consider selecting complementary indexing terms to improve the discoverability of the manuscript.

Authors’ response to comment #4: Thank you for your careful observation. We have revised the keywords in the revision:

Keywords: Genotype × environment interaction; hypoxia; growth recovery; quinoa; root traits; yield prediction

Introduction

Comment #5. The abbreviation GGE is introduced without being defined. Please spell out the full term at its first occurrence. In addition, the submitted manuscript does not include continuous line numbering, which makes the review process unnecessarily difficult. Please include line numbers throughout the manuscript.

Authors’ response to comment #5: Thank you for your valuable comment. We have addressed both issues in the revised manuscript. The abbreviation GGE has been defined as the genotype main effect plus genotype by environment interaction (GGE) at its first occurrence. In addition, continuous line numbering has been added throughout the revised manuscript to facilitate the review process.

Comment #6: The following sentence should be completely rewritten for clarity, grammar, and scientific style: "By integrating physiological, agronomic, and data-driven approaches, this study provides a comprehensive framework for identifying hypoxia-tolerant quinoa genotypes and improving selection strategies of breeding program for waterlogging tolerance in quinoa plants."

Authors’ response to comment #6: Thank you very much for your valuable comment. We have carefully checked the manuscript and revised it accordingly (Lines 78-82 of Page 4).

By integrating morphological, physiological, and biomass-related trait assessments during both hypoxic stress and post-stress recovery with multivariate analyses and machine learning-based prediction, this study provides a comprehensive framework for identifying hypoxia-tolerant quinoa genotypes and supporting breeding strategies for improved waterlogging tolerance.

Materials and Methods

Plant material

Comment #7: The Plant Material section requires substantially more detail. Please describe the origin of the germplasm, relevant characteristics of each genotype, the rationale for their selection, and whether any genotype was included as a reference or check. The current description is insufficient.

Authors’ response to comment #7: Thank you very much for your valuable comment. We have carefully checked the manuscript and revised it accordingly (Lines 85-98 of Page 4) as following:

Twenty-eight genotypes with name and passport data were used in this study (Table 1). Of these, Q1, Q4, Q4bias, and Q5 were selected by Dr. Redouane Choukrallah at the International Center for Biosaline Agriculture (ICBA, UAE) based on their superior performance under high-temperature conditions [25]. G13, G18, and Black quinoa were derived from interspecific crosses between Chenopodium quinoa and Chenopodium berlandieri, with G13 and G18 provided by Dr. Eric Jellen (Brigham Young University, USA) [20]. Cahuil and Isluga are Chilean germplasm originating from central and northern Chile, respectively, whereas NL-6 was selected from the humid lowlands of Chile and Sayaña originated from the Bolivian Highlands. Atlas is a breeding line developed for northwestern European environments, while 2-Want is regarded as a natural hybrid between Andean and Lowland quinoa [17,18,26,27]. In addition, Real1*Ruy937 and Surami*Ruy937 are stable F8 recombinant inbred lines (RILs) derived from their respective parental crosses and have been used in previous study investigating quinoa responses to abiotic stresses [13]. All quinoa genotypes were provided through a project funded by the Vietnamese Ministry of Science and Technology (Grant No. HNQT/SPĐP/07.17) under the bilateral cooperation program between the Governments of Vietnam and Argentina for the evaluation of quinoa under different ecological conditions in Vietnam.

Experimental method

Comment #8. Please verify the expression "moist tessue." It appears to contain a typographical error and should probably read "moist tissue paper."

Authors’ response to comment #8: Thank you very much for your valuable comment. We have carefully checked the manuscript and added the information accordingly (Lines 100-103 of Page 4) as following:

To ensure uniform germination, 100 seeds of each genotype were soaked in distilled water for 3 h and then germinated on moist tissue paper for 2 days at room temperature (approximately 28°C). Uniformly germinated seeds with radicles ~1.5 cm in length were selected for the experiment.

Comment #9. The sentence: "Then, the 14-old-seedlings were grown for 14 days..." contains grammatical errors. It should be revised (e.g., "Then, 14-day-old seedlings were grown for an additional 14 days...").

Authors’ response to comment #9: Thank you very much for your valuable comment. We have carefully checked and corrected the information accordingly (Lines 105-106 of Page 5) as following:

Then, 14-day-old seedlings were grown for an additional 14 days under either hypoxia or normoxia under hydroponic culture (Fig. 1).

Comment #10. Figure 1. The figure panels should follow the chronological order of the experimental procedure described in the manuscript. Based on the methodology, the logical sequence appears to be panel C, followed by B, and finally A. Furthermore, the figure legend is too brief and should provide a detailed explanation of each panel so that the figure can be understood independently of the main text.

Authors’ response to comment #10 Thank you for your valuable comment. We would like to retain the current panel arrangement because panels (a) and (b) introduce the normoxia and hypoxia hydroponic systems, while panel (c) presents the experimental workflow. To improve clarity, we have expanded the figure legend with additional information describing each panel:

Fig. 1. Experimental design: (a) Aerated hydroponic system (normoxia); (b) Non-aerated hydroponic system containing 0.1% agar solution (hypoxia); and (c) Experimental procedure and sampling timeline, including seed soaking (3 h), pre-germination (2 days), normoxia acclimation (14 days), hypoxia or normoxia treatment (14 days), recovery under normoxia (14 days), and measurements after treatment and recovery.

Comment #11: Please clearly define normoxia and hypoxia within the methodology when these treatments are first introduced. The experimental conditions should be explicitly described for readers unfamiliar with these terms.

Authors’ response to Comment #11: Thank you very much for your valuable comment. We have clarified the definitions of normoxia and hypoxia when these treatments are first introduced in the Materials and Methods section. The experimental conditions are now explicitly described to improve clarity for readers unfamiliar with these terms (Lines 110–114 of Page 5) as following:

Under normoxia, seedlings were aerated by two air pumps at opposite ends of the container to maintain dissolved oxygen (DO) concentration of >7.0 mg L−1. Under hypoxia, seedlings were grown in 0.1% (w/v) agar solution in deionized distilled water that is deoxygenated with a continuous flow of nitrogen to maintain a DO concentration of <1.0 mg L−1.

Comment #12: Experimental design. It is unclear how the experiment was arranged as a randomized complete block design (RCBD) given that two contrasting oxygen conditions (normoxia and hypoxia) were imposed. Please clarify how the experimental units were randomized and blocked under these conditions. In addition, define DO at its first occurrence and explain how oxigen concentration was monitored and maintained throughout the experiment.

Authors’ response to Comment #12: Thank you very much for your valuable comment. We have carefully reviewed and clarified the experimental design in the Materials and Methods section:

Under normoxia, seedlings were aerated by two air pumps at opposite ends of the container to maintain dissolved oxygen (DO) concentration of >7.0 mg L−1. Under hypoxia, seedlings were grown in 0.1% (w/v) agar solution in deionized distilled water that is deoxygenated with a continuous flow of nitrogen to maintain a DO concentration of <1.0 mg L−1. The experiment was arranged as a randomized complete block design (RCBD) with five replicates. Each replicate (block) comprised a pair of hydroponic containers placed side by side, one maintained under normoxia and the other under hypoxia. Genotypes were randomly allocated to experimental units within each container, and each experimental unit consisted of six plants. Samples were collected at the end of the hypoxia treatment and again 14 days after recovery. The remaining plants were continuously maintained under normoxia in plastic containers supplied with nutrient solution until physiological maturity, when the plants had completely senesced, and grain yield was determined at harvest. At each sampling time, five plants per replication were harvested for trait measurements.

Comment #13: The Experimental method section should be reorganized into clearer subsections with more detailed methodological descriptions. Several procedures are currently described too briefly to ensure reproducibility.

Authors’ response to Comment #13: Thank you very much for your valuable comment. We have carefully reviewed and revised Materials and Methods section as following sentences:

To ensure uniform germination, 100 seeds of each genotype were soaked in distilled water for 3 h and then germinated on moist tissue paper for 2 days at room temperature (approximately 28°C). Uniformly germinated seeds with radicles ~1.5 cm in length were selected for the experiment.The germinations were inserted through holes punched in a 7-mm-thick polystyrene board and held upright with plugs of silicone rubber. Each board, holding 96 seedlings, were placed over an opaque plastic container (386 mm × 256 mm × 135 mm), with the roots in deionized distilled water continuously aerated at 1.0 L min−1 by air pumps. Then, 14-day-old seedlings were grown for an additional 14 days under either hypoxia or normoxia under hydroponic culture (Fig. 1). The nutrient solution in hydroponic culture was prepared by adding 2 mL L⁻¹ each of A and B stock solutions to distilled water. A stock solution contained 37.51 g L⁻¹ NO₃⁻-N, 43.54 g L⁻¹ Ca, 39.99 g L⁻¹ K₂O, and 0.98 g L⁻¹ Fe-EDTA, whereas stock solution B contained 10.50 g L⁻¹ P₂O₅, 2.00 g L⁻¹ NH₄⁺-N, 6.68 g L⁻¹ S, 5.00 g L⁻¹ Mg, 394 mg L⁻¹ Mn, 140 mg L⁻¹ B, 100 mg L⁻¹ Zn, 24 mg L⁻¹ Cu, and 20 mg L⁻¹ Mo. Under normoxia, seedlings were aerated by two air pumps at opposite ends of the container to maintain dissolved oxygen (DO) concentration of >7.0 mg L−1. Under hypoxia, seedlings were grown in 0.1% (w/v) agar solution in deionized distilled water that is deoxygenated with a continuous flow of nitrogen to maintain a DO concentration of <1.0 mg L−1. The experiment was arranged as a randomized complete block design (RCBD) with five replicates. Each replicate (block) comprised a pair of hydroponic containers placed side by side, one maintained under normoxia and the other under hypoxia. Genotypes were randomly allocated to experimental units within each container, and each experimental unit consisted of six plants. Samples were collected at the end of the hypoxia treatment and again 14 days after recovery. The remaining plants were continuously maintained under normoxia in plastic containers supplied with nutrient solution until physiological maturity, when the plants had completely senesced, and grain yield was determined at harvest. At each sampling time, five plants per replication were harvested for trait measurements.

Comment #14: Please report the measurement units for all evaluated variables. In addition, the authors are encouraged to indicate the crop developmental stage at which the experiment was initiated using the BBCH scale.

Authors’ response to Comment #14: Thank you very much for your valuable comment. We have revised the manuscript accordingly by adding the measurement units for all evaluated variables.

Comment #15: The sentence: "SD was measured at a 2 cm height..."introduces SD without definition. Please verify all abbreviations throughout the manuscript and ensure that each term is written in full before its abbreviation is used.

Authors’ response to Comment #15: Thank you very much for your valuable comment. We have revised accordingly ( Stem diameter (SD) was measured at a 2 cm height.).

Data analysis

Comment #16: Please indicate the version of R used in the manuscript. In addition, provide the version number for every R package used and cite the software appropriately.

Authors’ response to Comment #16: All analyses in the revised manuscript were verified under R 4.5.1 and Python 3.13.5. Exact package versions (metan 1.19.0, FactoMineR 2.14, factoextra 2.0.0, agricolae 1.3.6, mclust 6.1.2, and others; scikit-learn 1.7.0, pandas 2.2.3, numpy 2.1.3, scipy 1.15.3 for the Python side) are listed in full in: S1_Analysis_Code/environment/R_package_versions.csv; R_sessionInfo.txt; and python/requirements.txt, included in the Supporting Information.

We also note, for the reviewer’s awareness, that the manuscript previously cited two different R versions for different package groups (4.1.3 and 4.1.1); we have reconciled this to a single, consistent statement in the revised Methods text (line 110). Changes made: Materials and Methods — Data analysis, line 110 (R version) and line 146 (Python version): added explicit R/Python version statement and citation of S1_Analysis_Code for full package versions.

Comment #17: The Data analysis section should be reorganized into shorter paragraphs with clearer subsectioning. The current presentation consists of excessively long paragraphs that reduce readability. Furthermore, please include appropriate methodological references for the statistical indices and machine-learning algorithms employed.

Please clarify what constitutes the environments in the genotype × environment (G×E) analysis. The statistical model used for the G×E analysis should also be explicitly presented within the methodology.

Authors’ response to Comment #17: Thank you very much for your valuable comment. We have revised the manuscript accordingly

Data analysis

Hyoxia tolerance index (HTI) analysis

The hypoxia tolerance index (HTI) was calculated as the ratio of the trait value under hypoxia to that under normoxia (HTI = XH/XN), where XH and XN represent the average values of the measured trait under hypoxia and normoxia, respectively.

Statistical analyses

The environment (E) refers to the two oxygen regimes to which seedlings were exposed, normoxia and hypoxia, rather than multiple field locations or growing seasons as in a conventional multi-environment trial. Statistical analyses were performed using R version 4.5.1 to assess the effects of genotype, environment, and their interaction using the following model:

Yijk = μ + Repk + Gi + Ej + (GE)ij + εijk (1);

where Yijk is the observed value of the trait for genotype i in environment j and replicate k; μ is the grand mean; Repk is the effect of replicate k; Gi and Ej are the main effects of genotype i and environment j, respectively; (GE)ij is the genotype × environment interaction effect; and εijk is the random residual error. An analysis of variance (ANOVA) based on this model was conducted to determine the significance of genotype, environment, and genotype × environment effects on the measured traits. When significant effects were detected, mean comparisons were made using the Least Significant Difference (LSD) test at a 5% significance level with the "agricolae" package (v1.3-6).

Hierarchical clustering and principal component analysis

Hierarchical clustering and principal component analysis (PCA) were utilized to explore patterns and relationships among variables, employing the "factoextra" (v2.0.0) and "FactoMineR" (v2.14) packages.

AMMI and GGE biplot analysis

To further characterize genotype-by-environment patterns, two complementary multivariate models were applied using the "metan" package (v1.19.0). The additive main effects and multiplicative interaction (AMMI) model retains genotype and environment as separate additive terms and decomposes only the residual genotype × environment interaction by singular value decomposition:

Yij = μ + Gi + Ej + Σₖ λₖ αᵢₖ γⱼₖ + εij (2);

where λₖ is the singular value of the k-th interaction principal component, and αᵢₖ and γⱼₖ are the corresponding genotype and environment scores. The genotype main effect plus genotype × environment interaction (GGE) biplot model instead removes only the environment main effect and jointly decomposes the combined genotype and genotype × environment variation:

Yij − μ − Ej = Σₖ λₖ ξᵢₖ ηⱼₖ + εij (3);

where ξᵢₖ and ηⱼₖ are the genotype and environment scores on principal component k.

In this study, two types of GGE biplots were generated. The which-won-where biplot was used to identify the highest-performing genotype(s) in different environments and to visualize potential mega-environments, whereas the mean versus stability biplot was used to evaluate genotype performance in terms of both mean performance and stability across environments.

Predictive model analysis

To identify the most suitable predictive model for estimating IY, nine regression algorithms representing linear, regularized linear, kernel-based, tree-based, instance-based, and neural-network approaches were evaluated. These included Ordinary Least Squares (OLS), Ridge Regression, Least Absolute Shrinkage and Selection Operator (LASSO), Elastic Net, k-Nearest Neighbors (KNN), Support Vector Machine with a linear kernel (SVM-Linear), Random Forest (RF), Gradient Boosting Machine (GBM), and Multi-Layer Perceptron (MLP).

Prior to model training, all predictor variables were screened for missing values and standardized to zero mean and unit variance to ensure comparability among features and to avoid scale-related bias. The dataset was randomly divided into training (80%) and testing (20%) subsets while preserving the statistical distribution of the target variable. Hyperparameters of each model were optimized using grid-search combined with k-fold cross-validation on the training dataset. The OLS model was used as the baseline linear regression approach. Ridge, LASSO, and Elastic Net were implemented to evaluate the effects of L2 regularization, L1 regularization, and combined regularization, respectively. RF and GBM were employed to capture complex nonlinear interactions among predictor variables through ensemble tree-learning strategies. KNN was used as a distance-based non-parametric method, while SVM-Linear was adopted to evaluate linear margin-based regression performance. The MLP model was implemented to assess the ability of artificial neural networks to learn nonlinear relationships between predictors and IY.

Model performance was assessed on the independent testing dataset using four complementary metrics: Pearson’s correlation coefficient (r), coefficient of determination (R²), root mean square relative error (RMSRE), and statistical significance (p-value). Pearson’s correlation coefficient quantified the strength of the linear association between observed and predicted values. The coefficient of determination (R²) measured the proportion of variance explained by each model. RMSRE was used to evaluate prediction accuracy while accounting for relative error magnitude. Scatter plots of observed versus predicted values were generated for all models. A 1:1 reference line was included to visually assess prediction agreement and systematic bias. Models exhibiting higher r and R² values together with lower RMSRE values were considered to have superior predictive performance. To ensure robustness and minimize overfitting, model stability was further examined using repeated k-fold cross-validation. The mean and standard deviation of evaluation metrics across validation folds were calculated and reported. All analyses were performed in Python (version 3.13.5) using the scikit-learn (v1.7.0), pandas (v2.2.3), NumPy (v2.1.3), and SciPy (v1.15.3) libraries.

Comment #18: It is strongly recommended that the complete analysis scripts (e.g., R and Python scripts) be provided as supplementary material. Given the use of multiple statistical models and machine-learning approaches, sharing the analytical workflow would substantially improve the transparency and reproducibility of the study.

Authors’ response to Comment #18: Thank you very much for your valuable comment. We have addressed this point in the revised manuscript by clarifying the availability of the analysis scripts and related materials in the appropriate section.

Results

Comment #19: The Results section should be reorganized according to the objectives of the study. Since each objective addresses a specific scientific question, the presentation of results should follow the same logical structure. In addition, each subsection should begin with a brief introductory sentence explaining the purpose of the analyses presented.

Authors’ response to Comment #19: Thank you very much for your valuable comment. We have revised the manuscript accordingly (Lines 206 to 227 in the manuscript)

Comment #20: Figure 2. The figure should distinguish between comparisons among treatments and comparisons among genotypes by using two different lettering systems (e.g., uppercase and lowercase letters). Please clarify this in both the figure and its legend.

Authors’ response to Comment #19: Thank you very much for your valuable comment. We have revised Fig. 2 accordingly. The genotypes are now arranged in ascending order of mean individual yield (IY) within each oxygen treatment (Hypoxia and Normoxia), allowing the LSD groupings to be interpreted more clearly. In addition, we have adopted two independent lettering systems as suggested: lowercase letters indicate comparisons among genotypes within the same oxygen treatment, whereas uppercase letters indicate comparisons between Hypoxia and Normoxia within the same genotype (LSD, α = 0.05). The interpretation of both lettering systems has been added to the figure legend. These revisions improve the clarity and readability of the figure.

Fig. 2. The effects of hypoxia stresses on individual seed yield (IY) of quinoa genotypes. Genotypes are ordered by ascending mean IY within each environment. Two independent lettering systems are used: lowercase letters compare genotypes within the same oxygen treatment, and UPPERCASE letters compare Hypoxia against Normoxia within the same genotype (Least Significant Difference test, P<0.05 for both)

Comment #20: The Materials and Methods section does not specify which software package or R package was used to perform the correlation analysis. Please include this information to improve reproducibility.

Authors’ response to Comment #20: Thank you very much for your valuable comment. We have addressed this point by providing the details of the software and R packages used for the correlation analysis in the Materials and Methods section, as described above, to improve the reproducibility of the study.

Comment #21: Pearson's correlation analysis. Given the large number of variables included, the Pearson correlation matrix is difficult to interpret and provides limited biological insight. The authors may consider complementing this analysis with a Principal Component Analysis (PCA), which would provide a more integrated visualization of the relationships among genotypes and traits while reducing redundancy among the figures.

Authors’ response to Comment #21: Thank you very much for your valuable comment and constructive suggestion. We agree that a Principal Component Analysis (PCA) provides a more integrated interpretation of the relationships among traits and genotypes. Accordingly, we have complemented the Pearson correlation analysis by adding a PCA in the revised manuscript:

Comment #22: .AMMI and GGE biplot analysis. Because only two environments were evaluated, the first two principal components explain all G+GE variation. This point should be explicitly clarified in the manuscript to avoid confusion among readers regarding the reported 100% explained variation.

Authors’ response to Comment #22: Thank you for your insightful comment. We have revised the Results section to clarify that the study involved only two environments (normoxia and hypoxia). Therefore, it is mathematically expected that the first two principal components account for 100% of the genotype × environment interaction variation. This explanation has been added to manuscript as following:

Because the analysis was based on only two environments, it is mathematically expected that the first two principal components explained 100% of the total G + GE variation, with PC1 and PC2 accounting for 68.45% and 31.55%, respectively (Fig. 5).

Comment #23: .The statement: "These results demonstrate a strong genotype × environment interaction and indicate that Isluga is a promising genetic resource for breeding quinoa with improved tolerance to hypoxia." is interpretative rather than descriptive. Such biological interpretation should be presented in the Discussion, whereas the Results section should focus on objectively reporting the findings

Authors’ response to Comment #23: Thank you for your valuable comment. We agree that this statement is interpretative and is more appropriate for the Discussion section. Accordingly, we have removed the sentence from the Results section, and the corresponding biological interpretation has been incorporated into the Discussion

Comment #24: .Hierarchical cluster analysis. The hierarchical clustering analysis provides limited additional biological insight beyond the GGE analysis. The authors may consider integrating a Principal Component Analysis (PCA) to summarize genotype and trait relationships more effectively and reduce redundancy among Figures 3–6.

Authors’ response to Comment #24: Thank you very much for your constructive suggestion. We respectfully chose to retain the hierarchical cluster analysis because it provides complementary information to the GGE biplot. While the GGE biplot focuses on genotype performance, stability, and genotype × environment interactions, hierarchical clustering classifies genotypes based on their overall phenotypic similarity across multiple traits, providing additional biological insight. In response to your suggestion, we have also incorporated a Principal Component Analysis (PCA) into the revised manuscript to provide an integrated visualization of the relationships among traits and genotypes.

Comment #25: Modeling of quinoa individual seed yield trait. The statement: "Overall, KNN, RF, SVM-Linear, and GBM were the most effective models for predicting IY, indicating that non-linear approaches better captured the variation in the dataset than traditional linear models." is interpretative rather than descriptive. The Results section should report the model performance objectively, whereas the interpretation of why certain algorithms outperformed others should be moved to the Discussion.

Authors’ response to Comment #25: Thank you very much for your valuable comment. We agree with your suggestion and have revised the manuscript accordingly.

Comment #26: In general, the Results section is insufficiently developed despite the large number of figures and tables presented. The manuscript currently emphasizes graphical outputs rather than answering the scientific objectives of the study. The section should be reorganized so that each subsection directly addresses one research objective, with figures and tables included only when they meaningfully support the scientific narrative.

Authors’ response to Comment #26: Thank you very much for your valuable and constructive comment. We have carefully revised and reorganized the Results section in accordance with the specific comments provided above

Discussion

Comment #27: Please include explicit cross-references to the relevant figures and tables throughout the Discussion. Referring to the corresponding results will strengthen the interpretation and improve coherence between the Results and Discussion sections.

Authors’ response to Comment #27: Thank you very much for your valuable comment. We have revised the Discussion section accordingly by adding explicit cross-references to the relevant figures and tables throughout the text

Comment #28: The Discussion should be reorganized into subsections that correspond to the specific objectives of the study. This structure would improve readability and provide a more logical interpretation of the findings.

Authors’ response to Comment #28: We sincerely thank the Reviewer for this valuable suggestion. We agree that organizing the Discussion into subsections corresponding to the specific objectives could enhance readability. However, after careful consideration, we respectfully request to retain the current structure because it provides a more integrated interpretation of the findings and follows the logical progression of the Results section.

Comment #29: . The manuscript should include a subsection discussing the limitations of the study. A balanced discussion of methodological limitations and potential sources of uncertainty would strengthen the scientific rigor of the manuscript.

Authors’ response to Comment #29: Thank you very much for your valuable comment. We agree with this suggestion and have added a Limitations of the Study subsection at the end of the manuscript, immediately following the Conclusions section:

Limitations

This study evaluated 28 genotypes under two researcher-imposed oxygen regimes (normoxia and hypoxia) rather than a multi-environment trial; the genotype × environment results reported here should therefore be read as genotype-by-treatment responses under these two conditions, not as evidence of broader environmental adaptation.

Conclusions Comment #30:

I am not convinced that this study should be presented as a classical genotype × environment (G×E) interaction study. Based on the experimental design described, the work appears to correspond to a two-factor factorial experiment involving genotype and oxygen treatment rather than a conventional multi-environment trial. The authors should clearly define what they consider to be the "environments" in this context, justify the application of G×E terminology, and provide a more detailed description of the implementation of the randomized complete block design (RCBD), particularly regarding the arrangement of experimental units under the two oxygen conditions.

Authors’ response to Comment #30: Thank you very much for your valuable comment. Most of the issues raised in this comment have been addressed through the revisions made in response to the previous comments. Specifically, we have clarified the definition of "environment" in the context of this study, justified the use of the genotype × environment (G×E) terminology, and provided a more detailed description of the RCBD implementation, including the arrangement and randomization of the experimental units under the two oxygen treatments.

Reviewer 2

Comment #1: Selection of the 28 genotypes: The selection of the 28 genotypes used in the study should be justified in greater detail. The manuscript states that they represent different genetic backgrounds, but it is not clear whether they originate from lowland or highland quinoa. More information on the origin, adaptation, and selection criteria of these genotypes should be provided (i.e., whether they are varieties, genebank accessions, progeny of crosses). Since no passport information is available, it would be useful to explain why these particular genotypes were chosen and how representative they are of quinoa diversity.

Authors’ response to Comment #1: Thank you for your valuable comment. We have revised the Plant materials section to provide additional information on the origin, breeding background, and selection of the 28 quinoa genotypes. The revised manuscript now clarifies that the genotypes represent diverse genetic backgrounds and eco-geographical origins, including breeding lines, recombinant inbred lines, interspecific derivatives, and germplasm from both highland and lowland regions. We have also described their sources and the Vietnam–Argentina collaborative project through which they were introduced into Vietnam. We believe these revisions better justify the selection of the genotypes used in this study:

Twenty-eight genotypes with name and passport data were used in this study (Table 1). Of these, Q1, Q4, Q4bias, and Q5 were selected by Dr. Redouane Choukrallah at the International Center for Biosaline Agriculture (ICBA, UAE) based on their superior performance under high-temperature conditions [25]. G13, G18, and Black quinoa were derived from interspecific crosses between Chenopodium quinoa and Chenopodium berlandieri, with G13 and G18 provided by Dr. Eric Jellen (Brigham Young University, USA) [20]. Cahuil and Isluga are Chilean germplasm originating from central and northern Chile, respectively, whereas NL-6 was selected from the humid lowlands of Chile and Sayaña originated from the Bolivian Highlands. Atlas is a breeding line developed for northwestern European environments, while 2-Want is regarded as a natural hybrid between Andean and Lowland quinoa [17,18,26,27]. In addition, Real1*Ruy937 and Surami*Ruy937 are stable F8 recombinant inbred lines (RILs) derived from their respective parental crosses and have been used in previous study investigating quinoa responses to abiotic stresses [13]. All quinoa genotypes were provided through a project funded by the Vietnamese Ministry of Science and Technology (Grant No. HNQT/SPĐP/07.17) under the bilateral cooperation program between the Governments of Vietnam and Argentina for the evaluation of quinoa under different ecological conditions in Vietnam.

Comment #2: Statistical consequences of the limited sample size: The relatively small number of genotypes raises statistical concerns, particularly because a large number of machine-learning models are evaluated. It is likely that the evaluated material contains considerable population structure, with genetically related genotypes originating from similar breeding backgrounds or geographic regions. This may substantially inflate the prediction accuracies obtained by the machine-learning models, despite the cross-validation used. The authors should discuss why such a small number of genotypes nevertheless resulted in extremely high prediction accuracies. It should also be discussed whether these high accuracies are partly explained by population structure rather than genuine predictive ability. If genetic marker data are available for these materials, they could help address this question.

Authors’ response to Comment #2: The reviewer's concern was valid and led us to find a real methodological problem. In reproducing the analysis behind Fig 7, we discovered that the originally reported metrics (e.g., KNN r = 1.000, R² = 1.000) had been computed in-sample: the models were refit on the complete dataset and evaluated on that same data, rather than on data held out from training. This produces exactly the kind of inflated accuracy the reviewer anticipated, though the specific mechanism is the evaluation methodology rather than population structure per se. We rebuilt the entire pipeline with a genuine held-out test split (80% train / 20% test), with hyperparameter grid-search restricted to the training set only (5-fold cross-validation), and all reported metrics now computed exclusively on the held-out test set. The corrected results are substantially lower than originally reported and show a different, more defensible pattern: only the tree-based and instance-based models (KNN, GBM, RF; r = 0.77–0.86, R² = 0.56–0.71) outperform the linear-regularized models, while the linear-kernel SVM and the multilayer perceptron do not exceed the linear models' performance (r = 0.47–0.51, R² = 0.11–0.23 across this group). We have updated every mention of these results throughout the manuscript and replaced Figure 7 with the corrected, held-out-test-set version. Full detail, including the retained training-set metrics for reference and an explicit, quantified account of the discrepancy from the originally reported figure, is documented in S1_Analysis_Code/README.md.

Changes made: Abstract, line 14; Results, line 225; Discussion, line 280; Conclusion, line 319: all updated to report the genuine held-out test-set metrics in place of the original in-sample values. Figure 7 replaced with the corrected version (Reviewer_Response/revised_figures/Fig7_revised.png), generated by S1_Analysis_Code/python/Fig07_ML_prediction.py.

Specific comments

Comment #3: The manuscripts lack page and line numbers, which should be included in a revised version

Authors’ response to Comment #3: Thank you for your valuable comment. We have revised the manuscript accordingly by adding page and line numbers throughout the revised version to facilitate the review process.

Comment #4: At the end of the first paragraph of the introduction, the authors should provide specific examples of previous studies investigating hypoxia tolerance in quinoa or other crops. This would better motivate the objectives of the present study.

Authors’ response to Comment #4: Previous studies have demonstrated substantial genetic variation in hypoxia tolerance across crop species such as rice [8], maize [9], soybean [10], wheat [11], and [12], with tolerant genotypes generally maintaining better root growth, photosynthetic capacity, biomass accumulation, and post-stress recovery under oxygen-deficient conditions. In quinoa, research has primarily focused on drought and salinity tolerance, whereas studies on hypoxia remain limited despite evidence of significant genotypic variation in growth and physiological responses to oxygen deficiency [13]. However, comprehensive evaluations that simultaneously assess plant performance during hypoxic stress and recovery across diverse quinoa germplasm remain scarce.

Comment #5: The introduction would also benefit from a clearer explanation of why this study provides a "comprehensive framework" for evaluating hypoxia tolerance.

Authors’ response to Comment #5: Thank you for this valuable suggestion. We have revised the Introduction to clarify why the present study provides a comprehensive framework for evaluating hypoxia tolerance. By integrating morphological, physiological, and biomass-related trait assessments during both hypoxic stress and post-stress recovery with multivariate analyses and machine learning-based prediction, this study provides a comprehensive framework for identifying hypoxia-tolerant quinoa genotypes and supporting breeding strategies for improved waterlogging tolerance.

Comment #6: Materials and Methods: The experimental procedures should be described in greater detail. It is currently not entirely clear how the plants were propagated before transfer into the hydroponic system and whether seed yield was measured from plants kept in the hydroponic system or put into pots with soil.

Authors’ response to Comment #6: Thank you very much for your valuable comment. We have carefully checked the manuscript and revised it accordingly:

To ensure uniform germination, 100 seeds of each genotype were soaked in distilled water for 3 h and then germinated on moist tissue paper for 2 days at room temperature (approximately 28°C). Uniformly germinated seeds with radicles approximately 1.5 cm in length were selected for the experiment.

The remaining plants were continuously maintained under normoxia in plastic containers supplied with nutrient solution until physiological maturity, when the plants had completely senesced, and grain yield was determined at harvest.

Comment #7: Materials and Methods: In the spirit of reproducible research, the analysis scripts should be made available.

Authors’ response to Comment #7: We agree, and in the process of assembling this we found that several of our original analysis scripts could no longer be located in usable form, having been intermixed with scripts from other, unrelated projects in our working directory. We therefore rebuilt the complete analysis pipeline from the raw phenotypic data, and independently verified every reconstructed script against the values reported in the submitted manuscript. The complete pipeline is provided as Supporting Information (S1_Analysis_Code.zip), containing: - R scripts reproducing Figs 2–6 and Tables 2–6 (LSD mean-comparison boxplot, Pearson correlation matrix, GGE biplots, hierarchical clustering, combined ANOVA, genotype-mean summary tables). - A Python script reproducing Fig 7 (nine-model machine-learning comparison for individual yield). - A README documenting, for every figure/table, which script produces it, how to run it, and — where our reconstruction could not exactly reproduce a previously reported value — an explicit, quantified account of the discrepancy rather than a silent adjustment. - The processed dataset and a single shared exclusion-list mechanism (see the response to the opening-paragraph comment on genotype exclusion, below) used consistently by every script.

Changes made: New Supporting Information file S1_Analysis_Code.zip; Data Availability Statement updated (Manuscript.docx, line 334) to reference it in place of the previous generic “available upon request” statement.

Comment #8: Results section: The abbreviations 'HTI', 'RSA' should be explained before their first use.

Authors’ response to Comment #8: Thank you very much for your valuable comment. We have carefully checked the manuscript and revised it accordingly:

Root surface area (RSA) was automatically calculated by software based on RL and RD of root in each class.

The hypoxia tolerance index (HTI) was calculated as the ratio of the trait value under hypoxia to that under normoxia (HTI = XH/XN), where XH and XN represent the average values of the measured trait under hypoxia and normoxia, respectively.

Comment #9: In several sections, the contents of the tables and figures are repeated almost verbatim in the text. Instead, the text in Results should focus on the key findings and trends rather than restating all numerical values. This is particularly noticeable in the sections describing the effects of hypoxia and in the correlation analysis.

Authors’ response to Comment #9: Thank you very much for your valuable comment. We have carefully checked the manuscript and revised it accordingly.

Comment #10: AMMI and GGE analyses: It is remarkable that the first two principal components explain 100% of the genotype-by-environment variation. Although this is mathematically possible with only two environments, this is unusual and should be explained explicitly to avoid confusing readers.

Authors’ response to Comment #10: Thank you for your insightful comment. We have revised the Results section to clarify that the study involved only two environments (normoxia and hypoxia). Therefore, it is mathematically expected that the first two principal components account for 100% of the genotype × environment interaction variation. This explanation has been added to avoid potential confusion for readers.

Comment #11: Discussion: The term "upland crop" should be explained more clearly.

Authors’ response to Comment #11: Thank you very much for your valuable comment. We have carefully checked the manuscript and revised it accordingly: Previous studies have reported substantial genotypic variation in root responses of upland crops such as soybean [10], wheat [11], barley [12] and sweet potato [35] to hypoxia.

Comment #12: Large parts of the Discussion simply repeat the Results. Instead, the authors should place their findings into the context of previous studies and discuss their biological implications.

Comment #13 and #14: (13) The purpose of the machine-learning prediction analysis is not entirely clear because no genetic marker information is included. The authors should explain more clearly what additional biological insight is obtained from these prediction models beyond demonstrating predictive performance. In particular, the authors should discuss whether these prediction models would also be applicable in segregating breeding populations derived from crosses between contrasting parents, where traits like response to hypoxia are genetically segregating. Could these models be useful for selection in breeding programs? (14) The final part of the Discussion should also address why the nonlinear machine-learning methods substantially outperform the linear models. Which biological sources of nonlinearity are likely responsible for these differences?

Authors’ response to Comment #13 and #14: Thank you very much for your valuable comment. We have carefully checked the manuscript and revised it accordingly:

On an independent held-out test set, KNN, GBM, and RF predicted individual yield more accurately (r = 0.77–0.86, R² = 0.56–0.71) than both the linear-regularized models (OLS, Ridge, LASSO, Elastic Net) and the other nonlinear approaches evaluated, linear-kernel SVM and a multilayer perceptron (r = 0.47–0.51, R² = 0.11–0.23). This pattern indicates that the advantage of KNN, GBM, and RF does not stem from nonlinearity in general, but from properties specific to instance-based and tree-based learning: both partition or weight the predictor space locally, allowing them to capture threshold-like responses and higher-order interactions among root, growth, and biomass traits without imposing a single global functional form, whereas a modest training sample may be insufficient to fit a neural network or a wide-margin classifier reliably (Fig. 8). Comparable sample-size-dependent contrasts between tree/instance-based and network- or margin-based learners have been reported in other crop genotype-to-phenotype prediction studies [39-41]. Because no genetic marker information was included, these models cannot by themselves resolve the specific physiological pathways or loci underlying variation in individual yield under hypoxia; their contribution is instead to rank and combine phenotypic traits by predictive value, which can guide phenotype-based selection ahead of, or independently from, marker development. Read together with the correlation analysis, which linked RL, RSA, LA, and biomass accumulation positively, and RD negatively, with IY, most of the models' predictive gain appears to derive from these already-identified trait relationships rather than from patterns undetectable by conventional statistics. Algorithms such as RF and GBM remain useful for weighting and combining these correlated traits into a single predictive score because they can accommodate non-linear responses, variable interactions, and threshold effects without requiring prior assumptions regarding data structure [29,42,43]. By contrast, the comparatively weak performance of the linear-kernel support vector machine used here [44] reflects its reliance on a single global margin, which is less able to represent the threshold-like or localized trait interactions that RF and GBM captured, while a similarly modest training-set size likely limited the multilayer perceptron. Overall, these results indicate that quinoa individual yield under hypoxia is shaped by trait interactions that are better captured by tree-based and instance-based learning than by either linear or other nonlinear model families, and that machine-learning-based trait prioritization can complement, but does not replace, marker-based approaches for dissecting the physiological and genetic basis of hypoxia tolerance.

Comment #15:. The Conclusion is largely repetitive and summarizes the Results and Discussion without providing additional perspectives. It could be substantially shortened.

Authors’ response to Comment #15: Thank you very much for your valuable comment. We have carefully checked the manuscript and revised it accordingly:

Hypoxia significantly affected the growth, root development, biomass accumulation, and yield of quinoa, with substantial variation among genotypes. RL and RSA were positively associated with IY, whereas RD showed negative relationships with several growth traits, highlighting the importance of maintaining root development under hypoxia. GGE biplot and cluster analyses identified Isluga as the best-adapted genotype to hypoxia, while G18, G13, EDK4, and 42-Test also exhibited favorable adaptation and performance. Amachuha genotype combined high mean performance with excellent stability, whereas Atlas, Cahuil, Red, and Q1 exhibited high stability and low genotype × environment interaction. Furthermore, among the nine machine-learning models compared on a held-out test set, the tree-based and instance-based methods (KNN, GBM, and RF) predicted individual yield more accurately than the linear-regularized models and the other nonlinear approaches tested (linear-kernel SVM, MLP), indicating that ensemble and distance-based learning captured trait interactions relevant to yield that simple linear combinations of traits did not. These findings describe genotype responses to two controlled oxygen regimes rather than to a sampled population of field environments; validating the promising genotypes and machine-learning-based trait prioritization identified here under field waterlogging conditions across multiple seasons or locations, and investigating the physiological, anatomical, and genetic mechanisms underlying hypoxia tolerance, are necessary next steps.

Comment #16:. Figure 2. The figure is rather noisy. Genotypes should be reordered from low to high grain yield values as the statistical tests are then better ot interprete.

Authors’ response to Comment #16: Thank you for your valuable comment. Figure 2 has been revised as suggested by reordering the genotypes from the lowest to the highest grain yield, improving the clarity of the figure and facilitating interpretation:

Fig. 2. The effects of hypoxia stresses on individual seed yield (IY) of quinoa genotypes. Genotypes are ordered by ascending mean IY within each environment. Two independent lettering systems are used: lowercase letters compare genotypes within the same oxygen treatment, and UPPERCASE letters

Comment #17: Figures 4 and 5. The two different GGE biplots should be introduced and briefly explained in the Materials and Methods section before they are presented in the Results.

Authors’ response to Comment #17: Thank you very much for your valuable comment. We have carefully checked the manuscript and revised it accordingly:

To further investigate genotype performance across different environments, additive main effects and multiplicative interaction (AMMI) model and the genotype main effect plus genotype by environment interaction (GGE) biplot analyses were conducted using the "metan" package (v1.19.0). In this study, two types of GGE biplots were generated. The which-won-where biplot was used to identify the highest-performing genotype(s) in different environments and to visualize potential mega-environments, whereas the mean versus stability biplot was used to evaluate genotype performance in terms of both mean performance and stability across environments.

Comment #18: Figure 6. The hierarchical cluster analysis should be discussed in the context of the putative genetic similarity among the genotypes. It is noteworthy that several highland accessions appear within the same clusters, suggesting that the clustering may partly reflect genetic differentiation between lowland and highland quinoa. This point should be discussed further.

Authors’ response to Comment #18: Thank you very much for your valuable comment. We have carefully checked the manuscript and added discussion accordingly:

The clustering pattern was consistent with the GGE biplot results. Genotypes in Cluster I were generally characterized by greater stability across normoxia and hypoxia, with relatively low genotype × environment interaction (Fig. 6). Cluster II contained genotypes such as G13 and 42-Test that were located near the hypoxia environment in the GGE biplot, suggesting moderate adaptation to hypoxia. Cluster III included several genotypes associated with hypoxia tolerance, particularly Isluga, G18, and EDK4. Among these, Isluga was identified as the winning genotype in the hypoxia sector of the “which-won-where” GGE biplot, indicating strong specific adaptation to hypoxia (Fig. 5), whereas G18 and EDK4 combined relatively high performance with acceptable stability (Fig. 6). The clustering pattern also reflected the genetic background and stress-adaptive characteristics of the evaluated genotypes. Highland genotypes such as Isluga and Sayaña clustered with stress-tolerant materials, whereas Chilean lowland genotypes such as NL-6 and Cahuil formed distinct groups, consistent with the reported eco-geographical differentiation of quinoa populations [26,27,38,39]. Similar clustering patterns have been reported under salinity and drought, where tolerant genotypes such as Atlas and 2-Want maintained superior root growth and stress performance [17,18], while our previous study under contrasting water regimes identified G18, Q1, and Q4 as stable, high-performing genotypes across drought and waterlogging environments [13,20]. The repeated identification of G18 as a superior genotype under drought, waterlogging, and hypoxia suggests broad adaptation to multiple abiotic stresses. Accordingly, G18 in this study, together with Isluga, G13, EDK4, and 42-Test, was identified by both the clustering and GGE biplot analyses as a promising genetic resource for breeding quinoa cultivars with improved hypoxia tolerance.

# Peer review: Decision Letter 1

**DOI:** 10.1371/journal.pone.0361654.r003

**Authors:** Eric Jellen

8 Sep 2026

Dear Dr. Loc Nguyen,

Thank you for submitting your manuscript to PLOS One. After careful consideration, we feel that it has merit but does not fully meet PLOS One’s publication criteria as it currently stands. Therefore, we invite you to submit a revised version of the manuscript that addresses the points raised during the review process.

Please thoroughly, and carefully, address my comments below, providing sufficient justification from the reputable, published scientific literature, regarding appropriateness of your experimental design and analytical approach. If this is not possible, you should consider submitting the manuscript to a different journal.

Please submit your revised manuscript by Nov 07 2026 11:59PM. If you will need more time than this to complete your revisions, please reply to this message or contact the journal office at plosone@plos.org. When you're ready to submit your revision, log on to https://www.editorialmanager.com/pone/ and select the 'Submissions Needing Revision' folder to locate your manuscript file.

- A letter that responds to each point raised by the academic editor and reviewer(s). You should upload this letter as a separate file labeled 'Response to Reviewers'.
- A marked-up copy of your manuscript that highlights changes made to the original version. You should upload this as a separate file labeled 'Revised Manuscript with Track Changes'.
- An unmarked version of your revised paper without tracked changes. You should upload this as a separate file labeled 'Manuscript'.

If you would like to make changes to your financial disclosure, please include your updated statement in your cover letter. Guidelines for resubmitting your figure files are available below the reviewer comments at the end of this letter.

If applicable, we recommend that you deposit your laboratory protocols in protocols.io to enhance the reproducibility of your results. Protocols.io assigns your protocol its own identifier (DOI) so that it can be cited independently in the future. For instructions see: https://journals.plos.org/plosone/s/submission-guidelines#loc-laboratory-protocols. Additionally, PLOS One offers an option for publishing peer-reviewed Lab Protocol articles, which describe protocols hosted on protocols.io. Read more information on sharing protocols at https://plos.org/protocols?utm_medium=editorial-email&utm_source=authorletters&utm_campaign=protocols.

As the corresponding author, your ORCID iD is verified in the submission system and will appear in the published article. PLOS supports the use of ORCID, and we encourage all coauthors to register for an ORCID iD and use it as well. Please encourage your coauthors to verify their ORCID iD within the submission system before final acceptance, as unverified ORCID iDs will not appear in the published article. *Only*  the individual author can complete the verification step; PLOS staff *cannot*  verify ORCID iDs on behalf of authors.

We look forward to receiving your revised manuscript.

Kind regards,

Eric Jellen, Ph.D.

Academic Editor

PLOS One

Journal Requirements:

If the reviewer comments include a recommendation to cite specific previously published works, please review and evaluate these publications to determine whether they are relevant and should be cited. There is no requirement to cite these works unless the editor has indicated otherwise.

Additional Editor Comments:

This manuscript has now been rejected by two reviewers. However, I feel the subject (flooding tolerance in quinoa) is sufficiently important to allow for a potential resubmission, provided the recommendations of both reviewers are thoroughly addressed, especially, as pointed out by Reviewer 2, that a sufficient number of examples be provided from other, reputable, published studies where multi-factorial analyses were applied successfully in conducting this type of analysis. Demonstrating a methodological precedent for the application of such methodologies would address the concerns of this reviewer regarding the experimental design and statistical analysis. Remember, you are going to have to be sufficiently persuasive to convince me, as well as two other reviewers, regarding the merit of your approach.

[Note: HTML markup is below. Please do not edit.]

Reviewers' comments:

Reviewer's Responses to Questions

**Comments to the Author**

Reviewer #2: (No Response)

Reviewer #3: All comments have been addressed

**********

2. Is the manuscript technically sound, and do the data support the conclusions?

Reviewer #2: No

Reviewer #3: Yes

**********

3. Has the statistical analysis been performed appropriately and rigorously?

Reviewer #2: No

Reviewer #3: N/A

**********

4. Have the authors made all data underlying the findings in their manuscript fully available?

The PLOS Data policy

Reviewer #2: Yes

Reviewer #3: Yes

**********

5. Is the manuscript presented in an intelligible fashion and written in standard English?

Reviewer #2: Yes

Reviewer #3: Yes

**********

Reviewer #2: This is the revised version of a manuscript that was previously reviewed and rejected by this reviewer. Of note, the final comments of the reviewer (Reviewer 2, comments #12-17) were not addressed in the response, possibly because of space limitations in the response area.

General comments

Overall, there has been some improvement in the manuscript, but it still suffers from several substantial problems.

Comment #1: One major issue, also raised by the other reviewer, is that this is essentially a controlled treatment experiment with a single environmental factor conducted under controlled conditions. Nevertheless, the authors apply methods such as GGE biplots and AMMI analysis, which were primarily developed for multi-environment trials, particularly multi-location field trials (Gauch et al. 2008; doi:10.2135/cropsci2007.09.0513). This experiment clearly does not represent such a multi-environment trial. This leads to several issues that were addressed by reviewer 1 previously but have not been adequately considered in the revised manuscript. Some of these concerns are outlined below.

Comment #2: The use of which-won-where, stability, AMMI, and GGE biplot analyses seems difficult to justify as analytical framework for this experiment. As stated above, GGE methodology was developed for multi-environment variety trials to address questions such as the delineation of mega-environments, environmental representativeness, and genotype adaptation. This is not the situation in the present experiment. There is only one experimentally manipulated environmental factor with the two levels control and hypoxia. Therefore, the environmental dimension is a single contrast: hypoxia versus control, which is different from a multi-environment trial involving multiple independent environments. Therefore, the main analysis should be the ANOVA/mixed-model analysis as described in the equation in line 148, which is sufficient to test the major experimental effects. Instead of the AMMI and GGE analyses, an additional analysis could be included that accounts for the strong correlations among the measured phenotypic traits, for example, MANOVA or a similar multivariate mixed-model approach such as PCA as shown in Figure 4.

In particular, the AMMI analysis, does not add much to the analysis of the phenotypic data because it essentially combines analysis of variance for the additive main effects with a multiplicative decomposition of the genotype by environment interaction (Gauch et al. 2008, reference above) as shown in the equation in line 164. However, with only two environments (control and hypoxia), the multiplicative interaction is limited to a single interaction dimension (as opposed to multiple environmental conditions in multilocation experiments). Thus, although an AMMI analysis is statistically valid for the data of this study, it is unnecessary and less informative than direct modeling of the factorial experiment. This fact becomes obvious by a comparison of the plots in Fig. 4, Fig. 5 and Fig. 6, which all show the same relationship among accessions, and in particular Figs. 5 and 6 being essentially identical. In contrast, A factorial model directly provides estimates of the hypoxia treatment effect, genotype effect, and genotype by treatment interaction. The concept of 'stability' has a limited meaning in this experiment because stability between control and hypoxia is not the same as stability across a range of environments in multilocation trials.

Overall, there is little advantage of GGE and AMMI analyses over a factorial linear or mixed-model analysis that explicitly estimates genotype effects, the hypoxia treatment effect, and genotype by treatment interactions while accounting for the randomized complete block design.

Comment #3: As already expressed in my review of the initial submission the analyses using ML models given the very small number of genotypes (n = 28) needs to be treated with caution. Despite the number of experimental observations, the effective sample size for predicting the response of new genotypes remains very small and machine-learning models are at risk of overfitting. In this context, the combination of hyperparameter optimization using grid search and cross-validation with such a small sample is problematic. If the same cross-validation procedure is used for both model/hyperparameter selection and estimation of predictive performance, the estimated prediction accuracy can be optimistically biased as has been shown in the literature (Varma & Simon 2006; doi:10.1186/1471-2105-7-91).

Comment #4: In addition, the unknown genetic relationships among the genotypes and the apparent population structure represented by the highland and lowland ecotypes are major concerns. With random cross-validation, predictive accuracy can be inflated when genetically related or population-structured genotypes occur in both the training and validation sets. Under such circumstances, a model can exploit population or family structure rather than predictor-response relationships resulting from causal genetic variation. This problem is well known in plant breeding populations (e.g., doi:10.3389/fpls.2020.592977). Given the very small sample size, it is quite possible that random cross-validation results in high apparent predictive ability partly because both training and validation sets contain genetically related genotypes or representatives of the same ecotype (e.g., lowland or highland). A simple test of the influence of population structure would be to train the models using only the highland accessions and predict the lowland accessions, and vice versa. Although this does not fully account for genetic relationships in the absence of molecular marker data, it provides a somewhat more stringent test of predictive ability across the two major ecotype groups. If population structure strongly contributes to the observed prediction accuracies, predictive performance should decrease under this cross-ecotype validation scheme compared with random cross-validation.

Therefore, the goal of the intended prediction should be clearly defined: Is the objective is to predict additional observations from the same genotypes, previously unseen genotypes from the same population, or genetically distinct quinoa germplasm? The appropriate cross-validation design and interpretation of predictive accuracy depend critically on the difference between these goals.

Specific comments:

Comment #5: It may be a matter of taste, but the manuscript now contains a large number of abbreviations that are not canonical (e.g., IY, etc) that increase the mental load required for reading. At least in the manuscript text writing out the full names of traits etc. instead of the abbreviation increases readability.

Comment #6: In table 1, the genotypes should be assigned to lowland and highland ecotypes.

Comment #7: Tables 3-6: These tables should become part of the supplementary information.

Comment #8: Figure 2: The different colors need to be explained in the caption.

Comment #9: Figure 4: The different colors and arrows, etc. should be explained in the caption.

Comment #10: The abstract should mention the experimental approach used for analysing hypoxia, and the operational definition of hypoxia should be given as well.

Comment #11: Lines 134ff: The phenological states in which the measurements were taken need to be described in greater detail, e.g., using the BBCH stages. When were the seeds collected? Did differences in flowering time among accessions affect the time of seed harvest? How many plants were used to measure the yield? How were the seeds cleaned before determining the yield?

Comment #12: Lines 212ff: The HTI is not consistently reported in this section. The usefulness of this metric is not clear as it corresponds in an inverse manner with the percent changes reported as well.

Comment #13: Line 230: "completely altered" - what exactly does this mean? This is a qualitative but not a quantitative descripton of the differences.

Comment #14: Lines 314ff: This part of the discussion mainly repeats the results section and does not add much to the interpretation of the results.

Comment #15: Lines 359ff: As mentioned above, the limitations in the analysis need to be addressed in more detail.

Comment #16: Lines 364ff: The conclusion section is repetitive with respect to the results and discussion sections. It should be more forward looking, and in particular provide an outlook about the importance of the observations in this study and the next steps for an improved understanding of this trait in quinoa.

Reviewer #3: The manuscript has improved substantially compared with the previous version, and the authors have addressed most of the concerns raised during the earlier review process. In particular, I appreciate the clarification of the experimental design, the inclusion of more detailed methodological descriptions, the addition of PCA, the provision of analysis scripts and supporting information, and the correction of the machine-learning evaluation procedure. The authors are also commended for transparently documenting the initial overestimation of prediction performance and replacing the original results with a more rigorous held-out test-set evaluation.

The study addresses an important topic, namely the evaluation of quinoa responses to hypoxia and the identification of traits and genotypes associated with tolerance to oxygen-deficient conditions. The dataset is valuable, and the integration of phenotyping, multivariate analyses, and predictive modeling represents a potentially useful contribution to quinoa breeding and stress physiology research.

Nevertheless, several issues should still be addressed before publication:

1. Use of genotype × environment terminology. Although the manuscript now clarifies that the two “environments” correspond to normoxia and hypoxia treatments, the study remains fundamentally a factorial genotype-by-treatment experiment rather than a conventional multi-environment trial. Terms such as “adaptation,” “stability,” “ideal genotype,” and similar expressions should be used more cautiously throughout the manuscript, and the limitations of interpreting G×E results from only two imposed treatments should be further emphasized.

2. Machine-learning interpretation. The correction of the modeling workflow is appreciated. However, the number of genotypes remains relatively limited, and no independent external dataset or marker information is available. Consequently, the machine-learning results should be presented primarily as exploratory phenotype-based prediction rather than as evidence of robust predictive models ready for breeding deployment. Some statements in the Discussion and Conclusions could be moderated accordingly.

3. Discussion section. The Discussion has improved considerably, but several passages still largely reiterate results already presented in the Results section. Greater emphasis on biological interpretation, physiological mechanisms of hypoxia tolerance, root adaptation strategies, and broader comparison with previous studies would further strengthen the manuscript.

4. English language and typographical issues. Numerous grammatical, spelling, and formatting inconsistencies remain in the manuscript, tables, and figure captions. Examples include misspellings such as “hyoxia,” “suface,” “physiologcial,” “gentotype,” “invididual,” and several formatting inconsistencies in tables and headings. A thorough language revision is strongly recommended before publication.

5. Developmental stage information. One of the previous review comments requested indication of the crop developmental stage using the BBCH scale. I was unable to identify a clear response to this point in the revised manuscript. If applicable, the authors should specify the developmental stage at treatment initiation or explain why BBCH staging was not used.

Finally, the manuscript is scientifically sound, the major methodological concerns have largely been addressed, and the study provides useful information regarding hypoxia tolerance in quinoa. I recommend minor revision to address the points above and improve the clarity and presentation of the manuscript before publication.

**********

what does this mean?). If published, this will include your full peer review and any attached files.

If you choose “no”, your identity will remain anonymous but your review may still be made public.

**Do you want your identity to be public for this peer review?**  For information about this choice, including consent withdrawal, please see our Privacy Policy

Reviewer #2: No

Reviewer #3: No

**********

[NOTE: If reviewer comments were submitted as an attachment file, they will be attached to this email and accessible via the submission site. Please log into your account, locate the manuscript record, and check for the action link "View Attachments". If this link does not appear, there are no attachment files.]

To ensure your figures meet our technical requirements, please review our figure guidelines: https://journals.plos.org/plosone/s/figures

You may also use PLOS’s free figure tool, NAAS, to help you prepare publication quality figures: https://journals.plos.org/plosone/s/figures#loc-tools-for-figure-preparation.

NAAS will assess whether your figures meet our technical requirements by comparing each figure against our figure specifications.

# Author comment: Author response to Decision Letter 2

**DOI:** 10.1371/journal.pone.0361654.r004

13 Sep 2026

1. Academic Editor — literature precedent for the multi-factorial approach

Comment. "…providing sufficient justification from the reputable, published scientific literature, regarding appropriateness of your experimental design and analytical approach… a sufficient number of examples be provided from other, reputable, published studies where multi-factorial analyses were applied successfully in conducting this type of analysis."

Response. No published application of GGE biplot or AMMI analysis to a single-factor, two-level controlled experiment was found. Gauch, Piepho and Annicchiarico (2008, Crop Science 48:866–889) and Forkman and Piepho (2014, Biometrics 70:639–647) define both methods for multi-environment yield trials; every crop application located uses three or more environments. The GGE/AMMI analysis is removed and replaced by PERMANOVA and MANOVA, for which two-level stress designs with correlated traits have precedent (Chaouachi et al. 2023, AoB PLANTS 15:plad022; Iftikhar-ud-Din and Hayat 2021, Sarhad J. Agric. 37:1250–1259; Asfaw et al. 2017, Front. Plant Sci. 8:733).

Changes made.

• See item 2.

2. Reviewer 2, Comments 1–2 — GGE/AMMI, which-won-where and stability analyses

Comment. "…the authors apply methods such as GGE biplots and AMMI analysis, which were primarily developed for multi-environment trials… This experiment clearly does not represent such a multi-environment trial." "…the main analysis should be the ANOVA/mixed-model analysis… an additional analysis could be included that accounts for the strong correlations among the measured phenotypic traits, for example, MANOVA or a similar multivariate mixed-model approach such as PCA… with only two environments… the multiplicative interaction is limited to a single interaction dimension… Figs. 5 and 6 being essentially identical."

Response. The GGE and AMMI analyses, Figs 5 and 6 and the text based on them are removed. With two environment levels the environment-centred GGE matrix has rank two, so PC1 + PC2 = 100% by construction. The multivariate analysis is now a PERMANOVA of the 19 traits of Table 2 (standardized, Euclidean distance, replicate + genotype × oxygen regime, 999 permutations): genotype 37.1%, oxygen regime 30.0%, interaction 18.8%, replicate 0.4%, residual 13.7% of the multivariate variation (all p = 0.001). A classical MANOVA on the same model gives Pillai's trace 0.991 for oxygen regime, 11.22 for genotype and 9.51 for the interaction (all p < 0.001). The hierarchical clustering (Fig 7) is retained as trait-profile clustering.

Changes made.

• Abstract, para. 12: delete "GGE biplot and cluster analyses identified Isluga … low genotype x environment interaction." Suggested replacement: "Permutational multivariate analysis of variance across 19 traits attributed 37.1% of overall phenotypic variation to genotype, 30.0% to oxygen regime, and 18.8% to their interaction (all p = 0.001), and hierarchical clustering on hypoxia-tolerance indices resolved three genotype groups with contrasting trait profiles. Under hypoxia, Amachuha and Isluga produced the highest individual yields, followed by EDK4 and Chadmo."

• Introduction, para. 18: "assess genotype adaptation and stability using the genotype main effect plus genotype by environment interaction (GGE) biplot and cluster analyses" → "characterize genotype × oxygen-regime effects across correlated traits using multivariate analysis of variance and hierarchical clustering".

• Methods, paras. 35–41: delete the "AMMI and GGE biplot analysis" subsection (equations 2–3). Add after "Hierarchical clustering and principal component analysis": "Multivariate analysis of variance. To account for correlations among the measured traits, a permutational multivariate analysis of variance (PERMANOVA; Anderson 2001) was fitted to the 19 traits in Table 2 with the vegan package (adonis2; Euclidean distance on standardized traits; terms added sequentially as replicate + genotype × environment; 999 permutations). A classical MANOVA (Pillai's trace) on the same model was computed for comparison."

• Results, paras. 57–60: delete "AMMI and GGE biplot analysis" and its three paragraphs. Insert after "Analysis for variance": "Multivariate analysis of variance. Across the 19 traits jointly, PERMANOVA attributed 37.1% of total phenotypic variation to genotype, 30.0% to oxygen regime, and 18.8% to the genotype × oxygen-regime interaction (all p = 0.001; replicate 0.4%, residual 13.7%; Table 4). The classical MANOVA confirmed all three effects (Pillai's trace: oxygen regime 0.991, genotype 11.22, interaction 9.51; all p < 0.001; Table 5)."

• Results, para. 62: end the clustering paragraph with "…indicates substantial variation in multi-trait response profiles among the evaluated germplasm" (delete "diverse adaptation patterns").

• Discussion, para. 69: delete the first five sentences ("The clustering pattern was consistent with the GGE biplot results" … "acceptable stability (Fig. 6)"). In the sentence on G18, end with "…is identified by both the yield comparison (Fig. 2) and the clustering analysis (Fig. 7) as a promising genetic resource".

• Conclusion, para. 74: delete "GGE biplot and cluster analyses identified Isluga … low genotype x environment interaction." Suggested replacement: "Genotype, oxygen regime and their interaction each explained a substantial share of multivariate phenotypic variation, and hierarchical clustering on hypoxia-tolerance indices distinguished three trait-profile groups; Amachuha and Isluga produced the highest individual yields under hypoxia."

• Figures and tables: remove Figs 5 and 6 and their captions (paras. 182–183); Fig 7 → Fig 5, Fig 8 → Fig 6. New Table 4 (PERMANOVA) and Table 5 (MANOVA): folder 05/Table4-5_PERMANOVA_MANOVA.

• Terminology (also Reviewer 3, Comment 1): "environment" → "oxygen regime" where the two treatments are meant; "genotype × environment interaction" → "genotype × oxygen-regime interaction"; delete "stability", "stable", "adaptation", "adapted", "ideal genotype" and "mega-environment" wherever they came from the GGE analysis.

• S1 File: R/PERMANOVA_MANOVA.R; the GGE scripts are kept under R/withdrawn/.

3. Reviewer 2, Comments 3–4 and 6 — machine learning with 28 genotypes, cross-validation, population structure; ecotypes in Table 1

Comment. "…the analyses using ML models given the very small number of genotypes (n = 28) needs to be treated with caution… If the same cross-validation procedure is used for both model/hyperparameter selection and estimation of predictive performance, the estimated prediction accuracy can be optimistically biased (Varma & Simon 2006)." "…the unknown genetic relationships among the genotypes and the apparent population structure represented by the highland and lowland ecotypes are major concerns… A simple test… would be to train the models using only the highland accessions and predict the lowland accessions, and vice versa… the goal of the intended prediction should be clearly defined." "In table 1, the genotypes should be assigned to lowland and highland ecotypes."

Response. Each genotype is assigned to the highland (13) or lowland (15) ecotype, and an Ecotype column is added to Table 1. The R1 random 80/20 split of observations placed every test genotype in the training set as well, so its accuracy reflected genotype recognition. The nine models were re-evaluated with three genotype-level designs — leave-one-genotype-out, leave-one-cluster-out (the three clusters of Fig 7) and the cross-ecotype test requested (train on highland, predict lowland, and the reverse) — with hyperparameters selected inside each training set by five-fold cross-validation grouped by genotype, and compared with a reference that uses no trait: the mean yield of the training observations under the same oxygen regime. No model reached that reference in any genotype-level design (best model R² 0.125 against 0.185 leave-one-genotype-out; 0.088 against 0.189 leave-one-cluster-out; 0.047 against 0.231 cross-ecotype), and within a regime the predictions did not rank genotypes. The prediction target — ranking genotypes not used in training for yield within a regime — is stated in Methods and is not achieved. The clusters of Fig 7 follow ecotype (Cluster I: 10 of 13 highland genotypes; Clusters II and III: 13 of 15 lowland genotypes; Fisher's exact test p = 0.0016).

Changes made.

• Table 1: add a column "Ecotype" (highland: 37-Test, 59-ALC, Amachuha, Cahuil, Chadmo, Isluga, Leucaboldo, Puno, Red, Riobamba, Ru2, Sayaña, Unknown; lowland: 2-Want, 23-GR, 42-Test, Atlas, Black quinoa, EDK4, G13, G18, NL-6, Q1, Q4, Q4bias, Q5, Real1 × Ruy937, Surami × Ruy937) and state the criterion of the classification in the table note.

• Methods, para. 44: replace "The dataset was randomly divided into training (80%) and testing (20%) subsets…" with: "The models were built to test whether the measured seedling traits can rank genotypes not used in training for individual yield within the same oxygen regime. Because each genotype contributed 6 observations (2 oxygen regimes × 3 replicates), models were evaluated at the genotype level in three ways: (i) leave-one-genotype-out — for each of the 28 genotypes in turn, all of its observations were withheld, the model was trained on the remaining 27 genotypes and yield was predicted for the withheld genotype; (ii) leave-one-cluster-out — each of the three genotype clusters of Fig. 7 was withheld in turn; (iii) cross-ecotype — models were trained on the 13 highland genotypes and tested on the 15 lowland genotypes, and vice versa. Hyperparameters were selected within each training set by five-fold cross-validation with folds grouped by genotype, so that model selection and performance estimation never shared data (Varma and Simon 2006). Each design was compared with a reference predictor that uses no trait, the mean yield of the training observations under the same oxygen regime, and with a random 80/20 split of individual observations." Delete "To ensure robustness… repeated k-fold cross-validation" (para. 45).

• Results, para. 64: replace the paragraph with: "Predictive accuracy for genotypes excluded from training was low for all nine models, and none exceeded the regime-mean reference (Fig. 8; Table 6). Under leave-one-genotype-out validation the best model, LASSO, reached R² = 0.125 (r = 0.36) against 0.185 for the reference; random forest reached 0.105. Withholding whole genotype clusters gave R² ≤ 0.09 (reference 0.19), and training on one ecotype and testing on the other gave pooled R² ≤ 0.05 (reference 0.23). Within a regime, observed and predicted yields of withheld genotypes showed no positive correlation. By contrast, a random split of individual observations, in which every test-set genotype also appeared in the training set, gave R² = 0.32 for random forest and 0.25 for KNN."

• Table 6 (new): the table below; file 05/Fig8_ML/Table_comparison_all_four.csv and Table_regime_mean_baseline.csv.

• Fig 8: observed versus predicted yield of withheld genotypes under leave-one-genotype-out validation, one panel per model (05/Fig8_ML/ML_LOGO_observed_vs_predicted); a single-panel random-forest version is provided.

• Discussion, para. 69: replace "Highland genotypes such as Isluga and Sayaña clustered with stress-tolerant materials, whereas Chilean lowland genotypes such as NL-6 and Cahuil formed distinct groups, consistent with the reported eco-geographical differentiation of quinoa populations [26,27,38,39]." with "The clusters also followed ecotype: Cluster I contained 10 of the 13 highland genotypes, and Clusters II and III 13 of the 15 lowland genotypes (Fisher's exact test, p = 0.002), consistent with the eco-geographical differentiation of quinoa populations [26,27,38,39]." (Cahuil is a highland genotype under the classification used.)

• Abstract, Discussion para. 70, Conclusion: delete the sentences on KNN, GBM and random forest accuracy (r = 0.77–0.86, R² = 0.56–0.71). Suggested sentence: "Under genotype-level validation (leave-one-genotype-out, leave-one-cluster-out and highland-versus-lowland cross-ecotype splits), no model predicted the individual yield of genotypes excluded from training better than the mean yield of the training genotypes under the same oxygen regime, so trait-based yield prediction does not transfer to unseen quinoa genotypes." References 40–45 become uncited if the paragraph is shortened.

• Limitations, para. 72: add that the ecotype assignment is not marker-based and that the machine-learning results are exploratory: no model predicted the yield of genotypes outside the training set, or across ecotypes, better than the regime mean.

• S1 File: python/ML_leave_cluster_out.py, ML_leave_one_genotype_out.py, ML_cross_ecotype.py, ML_baselines.py; data/genotype_ecotype.csv.

Table 6 (proposed). Predictive accuracy for individual yield under four validation designs; nine models and the no-trait reference.

Model Leave-one-genotype-out R² (r) Leave-one-cluster-out R² Cross-ecotype R² (r) Random row split R²

Regime mean of the training observations (no traits) 0.185 (0.433) 0.189 0.231 (0.481) 0.156

LASSO 0.125 (0.363) 0.088 -0.003 (-0.032) 0.159

Random forest 0.105 (0.358) 0.063 0.036 (0.273) 0.324

Elastic Net 0.072 (0.289) 0.070 0.033 (0.220) 0.062

Gradient boosting 0.062 (0.304) 0.088 -0.089 (0.094) 0.175

KNN 0.021 (0.327) -0.040 -0.103 (0.199) 0.250

Ridge 0.015 (0.256) 0.030 0.031 (0.280) 0.161

Linear SVM 0.008 (0.285) -0.020 0.047 (0.311) 0.119

OLS -0.186 (0.200) -0.243 -0.404 (0.258) 0.082

Multilayer perceptron -1.329 (-0.032) -1.191 -1.143 (0.083) -0.322

4. Reviewer 2, Comment 12 — HTI reporting

Comment. "Lines 212ff: The HTI is not consistently reported in this section. The usefulness of this metric is not clear as it corresponds in an inverse manner with the percent changes reported as well."

Response. The Results quoted, for each trait, a percentage change computed from the panel means, while Tables 3–6 tabulate the per-genotype ratio HTI; a mean of ratios differs from the ratio of means, so the two sets of numbers did not agree. The revised text reports one quantity, the mean of the per-genotype HTI, and gives a percentage change only as (1 − HTI) × 100.

Changes made.

• Results, paras. 50–53: replace the in-text values with the means of the per-genotype HTI from the regenerated tables (Table 3 panel summary, folder 05/Table3-6_HTI): RL1 0.61, RL2 0.70, RSA1 0.75, RSA2 0.70, RD1 1.28, RD2 1.01, PH1 0.69, PH2 0.73, NoL1 0.90, NoL2 0.82, LA1 0.53, LA2 0.64, SPAD1 0.92, SPAD2 0.95, SDW1 0.63, SDW2 0.56, RDW1 0.64, RDW2 0.74.

• Discussion, para. 67: "average HTI values of 0.57 and 0.58" → "0.61 and 0.70".

• Abstract, para. 12: re-read the percentage figures from the same table if they are kept.

• New Table 3 (proposed): panel means under normoxia and hypoxia and mean HTI per trait (05/Table3-6_HTI/HTI_panel_summary.csv); R1 Tables 3–6 can then move to Supporting Information (Comment 7).

5. Reviewer 2, Comment 13 — "completely altered"

Comment. "Line 230: 'completely altered' – what exactly does this mean? This is a qualitative but not a quantitative description of the differences."

Response. The statement is replaced by the rank correlation between the genotype rankings under the two regimes: Spearman rho = 0.25 (p = 0.21, n = 28), with a median shift of 6 rank positions. Amachuha ranks first under hypoxia (5.26 g plant⁻¹) and second under normoxia (6.20 g); Chadmo, first under normoxia (6.62 g), ranks fourth under hypoxia (4.55 g), behind Isluga (4.89 g) and EDK4 (4.57 g).

Changes made.

• Results, para. 54: replace "Although hypoxia reduced IY across genotypes, the ranking of genotypes was not completely altered. Chadmo consistently produced the highest IY under both conditions…" with "Hypoxia reduced IY in all genotypes and reordered them: genotype rankings under normoxia and hypoxia were weakly correlated (Spearman rho = 0.25, p = 0.21), with a median shift of 6 rank positions. Amachuha was the highest-yielding genotype under hypoxia (5.26 g plant⁻¹) and second under normoxia (6.20 g), whereas Chadmo, the highest under normoxia (6.62 g), ranked fourth under hypoxia (4.55 g), behind Isluga (4.89 g) and EDK4 (4.57 g)."

• Discussion, para. 68: "Chadmo, G18, G13, Amachuha, and Ru2 achieved the highest individual yields under hypoxia" → the five highest under hypoxia are Amachuha, Isluga, EDK4, Chadmo, 42-Test (Fig 2).

• Files: 05/Fig2_nang_suat_LSD/IY_genotype_ranks.csv, IY_rank_correlation.csv, IY_boxplot_letters.csv.

6. Reviewer 3, Comment 1 — terminology

Comment. "…the study remains fundamentally a factorial genotype-by-treatment experiment rather than a conventional multi-environment trial. Terms such as 'adaptation,' 'stability,' 'environment,' and 'ideal genotype' should therefore be used cautiously."

Response. These terms came from the GGE analysis, which is removed (item 2). The experiment is described as a genotype × oxygen-regime experiment.

Changes made.

• Whole text: "environment" → "oxygen regime" for the two treatments; "genotype × environment interaction" → "genotype × oxygen-regime interaction"; delete "stability", "stable", "adaptation", "adapted", "ideal genotype", "mega-environment" (see item 2).

7. Reviewer 3, Comment 2 — machine-learning results as exploratory

Comment. "…the machine-learning results should be presented primarily as exploratory phenotype-based prediction rather than as evidence of robust predictive models ready for breeding deployment."

Response. Under genotype-level validation no model predicted the yield of genotypes excluded from training better than the regime mean (item 3). The revised text makes no predictive claim for untested genotypes.

Changes made.

• See item 3: Methods para. 44, Results para. 64, Table 6, Fig 8, and the sentences suggested for the Abstract, Discussion and Conclusion; Limitations state that the results are exploratory.

Decisions for the corresponding author

• Numbering of the revised manuscript: R1 Tables 3–6 to Supporting Information (S1–S4 Tables), new Table 3 = panel means and mean HTI, Table 4 = PERMANOVA, Table 5 = MANOVA, Table 6 = validation table; Figs 5–6 removed, Fig 7 → 5, Fig 8 → 6.

• Table 1 note: the criterion of the highland/lowland classification.

• Fig 2: replicate points in one neutral colour (as regenerated) or genotype colours.

• Fig 3: the correlation matrix pools both regimes (168 observations); eight of twelve coefficients quoted in the R1 text agree within 0.10 with the regenerated values, SDW2–IY (0.14 → 0.40), RD1–SDW1 (−0.24 → −0.40), RD1–TDW1 (−0.23 → −0.41) and RD2–RSA2 (−0.39 → +0.19) do not.

• Fig 4: the regenerated PCA (56 genotype × regime means, 20 traits) gives PC1 = 48.55%, PC2 = 9.80%; the R1 values (45.38%, 8.85%) cannot be obtained from the deposited file.

• Genotype spelling: one spelling for each genotype in Table 1, text, figures and data (e.g. Real1 × Ruy937, Surami × Ruy937, EDK4, Ru2).

• Three data points to check against the record sheets: Isluga yields more under hypoxia (4.89 g) than under normoxia (3.48 g); Black quinoa root length under hypoxia at 30 DAS (662 cm) is nearly double its normoxia value (364 cm); the R1 Abstract values 1.38/0.74 g are shoot dry weight after recovery (SDW2), not total dry weight.

Files in S1 File behind the numbers

Item Script Output

Corrected dataset data/build_v2_rep1-3_corrected.py data/Hypoxia_and_Normoxia_Raw_data_Code_v2_rep1-3_corrected.csv; corrections_v2_changelog.csv

Screen of the deposited file data/audit/data_integrity_check_raw_csv.py flagged_10x_cells.csv; replicate_duplication_by_trait.csv

Table 2 R/ANOVA_mean_squares.R outputs/ANOVA/

Tables 3–6, new Table 3 R/HTI_summaries.R outputs/HTI/; HTI_panel_summary.csv

PERMANOVA, MANOVA R/PERMANOVA_MANOVA.R outputs/PERMANOVA/

Fig 2, rank correlation R/IY_boxplot_LSD.R outputs/IY_boxplot/

Fig 3 R/Correlation_matrix.R outputs/Correlation/

Fig 4 R/PCA_biplot.R outputs/PCA/

Fig 7, cluster × ecotype R/HCPC_clustering.R outputs/HCPC/

Machine learning, four designs python/ML_random_split.py; ML_leave_cluster_out, ML_leave_one_genotype_out, ML_cross_ecotype outputs/ML_random_split, ML_leave_cluster_out, ML_leave_one_genotype_out, ML_cross_ecotype/

Reference predictor python/ML_baselines.py outputs/ML_baselines/

Attachment Submitted filename: Responses to reviewers comments.docx

# Peer review: Decision Letter 2

**DOI:** 10.1371/journal.pone.0361654.r005

**Authors:** Eric Jellen

14 Oct 2026

<p>Genotypic variation and root traits associated with hypoxia tolerance in Quinoa (Chenopodium quinoa Willd.)

PONE-D-26-29301R2

Dear Dr. Loc Nguyen,

We’re pleased to inform you that your manuscript has been judged scientifically suitable for publication and will be formally accepted for publication once it meets all outstanding technical requirements.

Within one week, you’ll receive an e-mail detailing the required amendments. When these have been addressed, you’ll receive a formal acceptance letter and your manuscript will be scheduled for publication.

An invoice will be generated when your article is formally accepted. Please note, if your institution has a publishing partnership with PLOS and your article meets the relevant criteria, all or part of your publication costs will be covered. Please make sure your user information is up-to-date by logging into Editorial Manager at Editorial Manager® and clicking the ‘Update My Information' link at the top of the page. For questions related to billing, please contact billing support.

If your institution or institutions have a press office, please notify them about your upcoming paper to help maximize its impact. If they’ll be preparing press materials, please inform our press team as soon as possible -- no later than 48 hours after receiving the formal acceptance. Your manuscript will remain under strict press embargo until 2 pm Eastern Time on the date of publication. For more information, please contact onepress@plos.org.

Kind regards,

Eric Jellen, Ph.D.

Academic Editor

PLOS One

Additional Editor Comments (optional):

Reviewers' comments:

Reviewer's Responses to Questions

**Comments to the Author**

Reviewer #4: All comments have been addressed

Reviewer #5: All comments have been addressed

**********

2. Is the manuscript technically sound, and do the data support the conclusions?

Reviewer #4: Yes

Reviewer #5: Yes

**********

3. Has the statistical analysis been performed appropriately and rigorously?

Reviewer #4: Yes

Reviewer #5: Yes

**********

4. Have the authors made all data underlying the findings in their manuscript fully available?

The PLOS Data policy

Reviewer #4: Yes

Reviewer #5: Yes

**********

5. Is the manuscript presented in an intelligible fashion and written in standard English?

Reviewer #4: Yes

Reviewer #5: Yes

**********

Reviewer #4: Waterlogging-induced hypoxia is a real problem for quinoa for cultivation in new environments outside the Andes, especially with climate changes could affect the meteorological conditions during the rainy season.

The paper is well written with a good presentation of the materials and methods that help for providing interesting results in conclusion. The discussion of these results is important for establishing how this first study is useful for understanding the Waterlogging-induced hypoxia but with some limitations and restriction to the lot of genotypes used in the experimentation.

All comments from previous reviewers have been addressed in this version of the manuscript.

I consider this paper ready for publication after this round of revision and all the responses given by the authors.

Reviewer #5: The authors appear to have adequately responded to previous reviewer comments. I have no additional comments.

**********

what does this mean?). If published, this will include your full peer review and any attached files.

If you choose “no”, your identity will remain anonymous but your review may still be made public.

**Do you want your identity to be public for this peer review?**  For information about this choice, including consent withdrawal, please see our Privacy Policy

Reviewer #4: No

Reviewer #5: No

**********

# Editor report: Acceptance letter

**DOI:** 10.1371/journal.pone.0361654.r006

**Authors:** Eric Jellen

PONE-D-26-29301R2

PLOS One

Dear Dr. Loc Nguyen,

I'm pleased to inform you that your manuscript has been deemed suitable for publication in PLOS One. Congratulations! Your manuscript is now being handed over to our production team.

At this stage, our production department will prepare your paper for publication. This includes ensuring the following:

* All references, tables, and figures are properly cited

* All relevant supporting information is included in the manuscript submission,

* There are no issues that prevent the paper from being properly typeset

You will receive further instructions from the production team, including instructions on how to review your proof when it is ready. Please keep in mind that we are working through a large volume of accepted articles, so please give us a few days to review your paper and let you know the next and final steps.

Lastly, if your institution or institutions have a press office, please let them know about your upcoming paper now to help maximize its impact. If they'll be preparing press materials, please inform our press team within the next 48 hours. Your manuscript will remain under strict press embargo until 2 pm Eastern Time on the date of publication. For more information, please contact onepress@plos.org.

You will receive an invoice from PLOS for your publication fee after your manuscript has reached the completed accept phase. If you receive an email requesting payment before acceptance or for any other service, this may be a phishing scheme. Learn how to identify phishing emails and protect your accounts at https://explore.plos.org/phishing.

If we can help with anything else, please email us at customercare@plos.org.

Thank you for submitting your work to PLOS One and supporting open access.

Kind regards,

PLOS One Editorial Office Staff

on behalf of

Dr. Eric Jellen

Academic Editor

PLOS One
